# The EpiDiverse Plant Epigenome-Wide Association Studies (EWAS) Pipeline

**DOI:** 10.3390/epigenomes5020012

**Published:** 2021-05-04

**Authors:** Sultan Nilay Can, Adam Nunn, Dario Galanti, David Langenberger, Claude Becker, Katharina Volmer, Katrin Heer, Lars Opgenoorth, Noe Fernandez-Pozo, Stefan A. Rensing

**Affiliations:** 1 Plant Cell Biology, Department of Biology, University of Marburg, 35043 Marburg, Germany; nilaycan@biologie.uni-marburg.de (S.N.C.); noe.fernandezpozo@biologie.uni-marburg.de (N.F.-P.); 2 ecSeq Bioinformatics GmbH, 04103 Leipzig, Germany; adam.nunn@ecseq.com (A.N.); david.langenberger@ecseq.com (D.L.); 3 Bioinformatics Group, Department of Computer Science, University of Leipzig, 04107 Leipzig, Germany; 4 Plant Evolutionary Ecology, Institute of Evolution and Ecology, University of Tübingen, Auf der Morgenstelle 5, 72076 Tübingen, Germany; dario.galanti@uni-tuebingen.de; 5 Gregor Mendel Institute of Molecular Plant Biology (GMI), Austrian Academy of Sciences, Vienna BioCenter (VBC), Dr. Bohr-Gasse 3, 1030 Vienna, Austria; claude.becker@gmi.oeaw.ac.at; 6 Genetics, Faculty of Biology, Ludwig-Maximilians-University München, 82152 Martinsried, Germany; 7 Department of Forest Genetic Resources, Nordwestdeutsche Forstliche Versuchsanstalt (NW-FVA), 37079 Göttingen, Germany; Katharina.Volmer@nfp.niedersachsen.de; 8 Conservation Biology, Department of Biology, University of Marburg, 35043 Marburg, Germany; katrin.heer@uni-marburg.de; 9 Plant Ecology and Geobotany, Department of Biology, University of Marburg, 35043 Marburg, Germany; 10 Centre for Biological Signaling Studies (BIOSS), University of Freiburg, 79104 Freiburg, Germany; Lars.Opgenoorth@Staff.Uni-Marburg.DE; 11 SYNMIKRO Center for Synthetic Microbiology, University of Marburg, 35043 Marburg, Germany

**Keywords:** EWAS, GWAS, plant epigenetics, DNA methylation, non-model species, pipeline

## Abstract

Bisulfite sequencing is a widely used technique for determining DNA methylation and its relationship with epigenetics, genetics, and environmental parameters. Various techniques were implemented for epigenome-wide association studies (EWAS) to reveal meaningful associations; however, there are only very few plant studies available to date. Here, we developed the EpiDiverse EWAS pipeline and tested it using two plant datasets, from *P. abies* (Norway spruce) and *Q. lobata* (valley oak). Hence, we present an EWAS implementation tested for non-model plant species and describe its use.

## 1. Introduction

Epigenetics describes DNA or chromatin modifications that might change transcriptional activity without altering the DNA sequence and might be propagated somatically or through the germline. Epigenetic modifications such as DNA methylation and histone modifications (acetylation, phosphorylation, ubiquitylation, sumoylation) may affect the chromatin structure and, thereby, the access to genetic information [1]. Of these epigenetic modifications, methylation currently is the most intensively studied in plants as it can be easily assessed. DNA methylation is an epigenetic modification consisting of the addition of a methyl group (CH_3_) to the fifth carbon of the cytosine (C). Epigenetic mechanisms can alter phenotypic traits [2]. It was shown that DNA methylation may play a crucial role in gene expression regulation, e.g., of plant defense response under various environmental stresses [3]. DNA methylation may lead to heritable epigenetic information and transgenerational epigenetics describes the lack of resetting mechanisms of epigenetic states between generations. Epialleles are responsible for this heritable phenotypic variation and plants seem to have this type of inheritance in contrast to mammals [4]. There are very few known examples of natural epialleles, suggesting that epiallelic variation is very rare in nature, compared to allelic variation [5]. One of the first discovered natural plant phenotypes not based on a change in the DNA sequence was *Linaria vulgaris* (toadflax) [6]. This study revealed that mutant alleles showed high DNA methylation but low gene expression and a clear coincidence between the revertant phenotype and the degree of DNA methylation at the Lcyc locus [6]. Another example of epimutation alleles was first described by Barbara McClintock in maize by focusing on the effect of suppressor–mutator (Spm) transposons on gene expression [7]. Moreover, Cnr mutants in tomato showed colorless, non-ripening fruits and this was found to be caused by a mutant allele at the LeSPL–CNR locus [8]. The mutant phenotype was found to be associated with increased DNA methylation at the promoter region; the upstream promoter of LeSPL–CNR coincides with a TE that is heavily methylated in both wildtype and Cnr mutant. Finally, many epimutable alleles have been defined in Arabidopsis, and they all seem to involve TEs or other repetitive sequences [5]. DNA methylation also leads to transposon mobility, potentially affecting both short- and long-term adaptation to environmental conditions [9,10,11]. An example of epigenetic adaptation to temperature is vernalization observed in *Arabidopsis thaliana* ecotypes, which is regulated by flowering locus (FLC), where cold stress triggers H3K27me3 and H3K9me deposition in the FLC chromatin [12].

DNA methylation may occur in different nucleotide contexts. In animals, only Cs in CG contexts are methylated, whereas in plants [13], DNA methylation can be symmetrical (CG and CHG contexts) or asymmetrical (CHH, where H represents A, T, or C) [14,15,16,17]. The different contexts have different maintenance mechanisms [18]. Whole-genome bisulfite sequencing (WGBS) and reduced representation bisulfite sequencing (RRBS) are widely used methods to determine DNA methylation at a single-base resolution [19,20]. One goal in plant epigenetics is to detect positions or regions that are differentially methylated due to treatment or different environments, and multiple samples are required since differentially methylated positions (DMPs) are called using statistical approaches [21]. Differentially methylated regions (DMRs) are genomic regions where multiple adjacent positions reveal differential methylation [22]. 

Interest in understanding the genetic architecture of complex traits led to association studies to relate genetics and epigenetics with phenotypic traits. Testing genetic variation across genomes of individuals to reveal genotype–phenotype associations is made possible by genome-wide association studies (GWAS), which have frequently been used for human disease studies [23,24] and enabled the detection of many genetic variants significantly associated with complex human diseases. Results obtained from GWAS have been clinically reliable and help to develop new treatments for multiple diseases from diabetes to schizophrenia [25,26]. In plants, GWAS is a powerful tool for understanding complex traits, useful to discover the genetics related to important traits in agriculture and to accelerate breeding programs [27]. It has been applied to many crop species, e.g., maize (*Zea mays*), wheat (*Triticum aestivum*), rice (*Oryza sativa*), soybean (*Glycine max),* sorghum (*Sorghum bicolor*), barley (*Hordeum vulgare*), cotton (*Gossypium hirsutum*), and the model species Arabidopsis [28,29,30,31]. Moreover, GWAS is used to reveal genomic regions related to physiological, agronomic, and fitness traits such as plant height, stress tolerance, flowering time, kernel number, and grain yield [28,29,30,32], and identified genes connected with geographical deviation and adaptation in rice domestication [33]. Additionally, GWAS have also been used with genetic engineering, e.g., transgenic drought tolerance in maize was developed after the discovery of ZmVPP1 [34], and this led to an increment of studies using genome editing on target genes [35]. However, many diseases and disorders in humans including cancer show an epigenetic association [28,29,30]. Due to that, epigenome-wide association studies (EWAS) as a counterpart of GWAS have also been used in human studies [36]. EWAS is a powerful method to reveal epigenetic variation associated with biological traits [22,37]. Transgenerational epigenetic marks can be transmitted to descendants through mitosis (in case of vegetative propagation) or meiosis (sexual reproduction) [38]. The methylation variation of the same gene between different plants is called epialleles and can lead to different phenotypes that are heritable. Mutants of *Linaria vulgaris* are an example of transgenerational epigenetic inheritance [6]. Mechanisms involved in transgenerational inheritance of epigenetic marks are not fully understood but data showed that DNA methylation easily passes through generations and many studies focus on this mark [39]. Histone modification can also affect gene function and phenotype; however, it has been largely ignored in EWAS due to technological limitations and sample availability. Germline cells in plants are inherited from somatic cells and therefore can contribute to the heritability of epigenetic marks. Plants can sense environmental changes during their vegetative growth, and it may lead to epigenetic changes in cell lines that generate a germline [9]. Studies showed that stress-induced transgenerational reactions depend on DNA methylation in Arabidopsis [40,41]. Therefore, heritable epialleles may affect plant evolution, phenotypic traits, and fitness. Since many of the plants go through asexual propagation, meiotic epigenetic resetting does not occur, and information is carried to the next generation more effectively than in sexual reproduction [42]. Epigenetic changes are dynamic, making it difficult to discriminate significant relationship between phenotype and epigenetic mechanisms—a major challenge of EWAS [43] and common issues both for GWAS and EWAS are dealing with missing and big data [44]. Thus far, there has been very scarce use of EWAS for plants (for example, a PubMed search for “ewas plant” returned seven hits 19 February 2021, while “ewas human” returned 131). Published examples include DNA methylation variation in *Quercus lobata* (valley oak) associated with climatic gradients [45], and EWAS has been successfully applied to identify the epigenetic change that causes the metastable somaclonal variant in *E. guineensis* (oil palm) [46]. Another study with stone pine (*Pinus pinea)* showed that there was a remarkable level of phenotypic plasticity. Vegetatively propagated *P. pinea* trees showed a high degree of DNA methylation under different environmental conditions [47]. Several EWAS tools have been developed, yet most of them are hardcoded for human studies such as *GLINT* [48] or not able to deal with missing data such as EWAS: epigenome-wide association study software v2.0 [49]. However, there is one tool not hard coded that also accounts for genetic data, is compatible with all species, and allows missing data imputation, namely, the *GEM R package* [50], hence chosen for this study.

**Figure 1:**
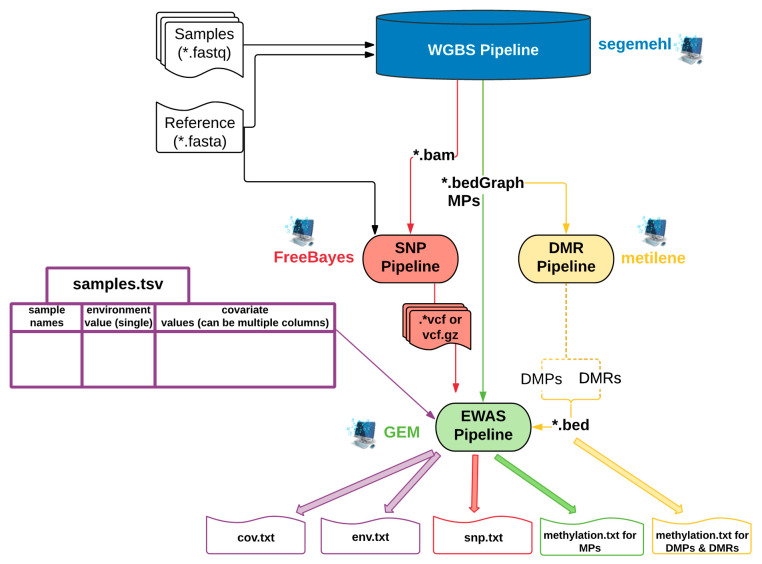
The EpiDiverse EWAS pipeline workflow and its interaction with the WGBS, SNP, and DMR EpiDiverse pipelines. Utilized packages or software were specified next to pipeline names. The EpiDiverse epigenome-wide association studies (EWAS) pipeline requires a tab-separated sample.tsv file (shown with purple frame) to specify climatic data and covariate(s) for group determination (can be sampling site, geographical location, or treatment group) and methylation data. As methylation input types, it accepts methylation calls (green arrow) and differentially methylated positions/differentially methylated regions (DMPs/DMRs) (yellow arrow), which can be provided by the whole-genome bisulfite sequencing (WGBS) and the DMR pipelines, respectively. The EWAS pipeline allows running three different models to find epigenetic markers associated with the environment (E), genetic variation (G), or the combination of both (GxE). G and GxE models need single nucleotide polymorphism (SNP) information (red arrow), which can be directly calculated by the SNP pipeline using bisulfite sequencing data, or, as for all other inputs, it can be provided by users. See Figures S1–S4 for more detail. * indicates multiple files with the same extension in a specified directory.

Here, we present the EpiDiverse EWAS pipeline, developed in the context of the EpiDiverse ITN network (https://epidiverse.eu/, accessed on 1 March 2021), which studies the effects of epigenetics in natural variation, stress responses, and acclimations of plants [51]. We aimed at realizing parts of the research agenda of EpiDiverse outlined in Richards et al. (2017) [51]. In EpiDiverse, a set of bioinformatics pipelines was developed to facilitate epigenetic analyses based on DNA methylation, especially for non-model plants. These pipelines are modular and scalable and can easily connect their inputs and outputs (Figure 1), providing a suite of useful tools for whole-genome bisulfite sequencing (WGBS) methylation calling, single nucleotide polymorphism detection (SNP), differentially methylated position, and region (DMP and DMR) detection and EWAS (https://github.com/EpiDiverse, accessed on 1 March 2021). The software included in the WGBS and DMR pipelines was selected from the best performing tools in benchmarking studies [52,53]. Here, we describe and test the performance of the EpiDiverse EWAS pipeline using four different input types from two non-model plant datasets and test the effect of missing data.

## 2. Results and Discussion

### 2.1. EpiDiverse EWAS Pipeline Workflow

The EpiDiverse EWAS pipeline is based on functions implemented in the GEM R package [50] and extends them by multiple features that allow the use of methylation calls and differential methylation data, with optional analysis of methylation quantitative trait loci (methQTLs) for diploid organisms from variant call data in which methQTL is an epigenetic marker that coincides with a quantitative trait locus (QTL). Additionally, missing data filtering with the GEM R package was modified, and estimation is conducted with beta distribution because there is evidence that the existing method biases the calculation of FDR values. This issue is made apparent when the methylation data are subdivided, e.g., by chromosome/scaffold: since the global methylation values are calculated from the remaining positions for the sample, the *p*-values themselves vary wildly for the same positions, depending on how many other positions are present during the analysis.

Additional graphs are generated (sequence dot plots, Manhattan plots) to help the user to understand results better and observe more visual outputs. The EpiDiverse EWAS pipeline performs epigenome-wide association studies, employing three models implemented in the GEM R package. We preferred GEM over other EWAS tools because, for example, *GLINT* [48] is hard coded for use in Illumina human methylation arrays, and *EWAS: epigenome-wide association study software v2.0* [49] is not able to estimate missing data.

The EWAS pipeline is part of the EpiDiverse toolkit, which provides tools for mapping WGBS data and methylation calling (WGBS pipeline), calculation of differential methylation (DMR pipeline), and estimation of genetic variants from bisulfite sequencing (SNP pipeline) (Figure 1). Bisulfite sequencing raw data can be processed by the WGBS pipeline to produce methylation calls, variant files in the SNP pipeline, and DMPs and DMRs in the DMR pipeline. The EWAS pipeline can use as input any combination of results from the other EpiDiverse pipelines (Figure 1), or users can provide their input files. 

The pipeline was built using Nextflow [54], a workflow tool for running tasks across multicompute infrastructures in a portable manner. It comes with docker containers to facilitate the installation process. The dependencies for the pipeline can be managed by Conda (https://docs.conda.io/en/latest/miniconda.html, accessed on 1 March 2021), Singularity [55], and/or Docker [56].

#### 2.1.1. Input Types for the EWAS Pipeline

Input can be derived from other EpiDiverse pipelines (WGBS, SNP, DMR) or user-provided and is combined with a user-provided, tab-separated sample sheet file to submit EWAS analysis (Figure 1). This sample sheet file has sample identifiers in the first column, environment values in the second column, and single/multiple covariates after the environmental values. This file is required to use sample names as a key and derive covariates (Figure S4). To account for genetic interaction, the EWAS pipeline needs an SNP genotype matrix encoded by 1, 2, 3 for major homozygote (AA), heterozygote (AB), and minor homozygote (BB) variants in vcf, bcf, or zipped vcf.gz format. The SNP pipeline can be used to extract genetic variation files in vcf.gz format to derive this input. 

**Figure 2:**
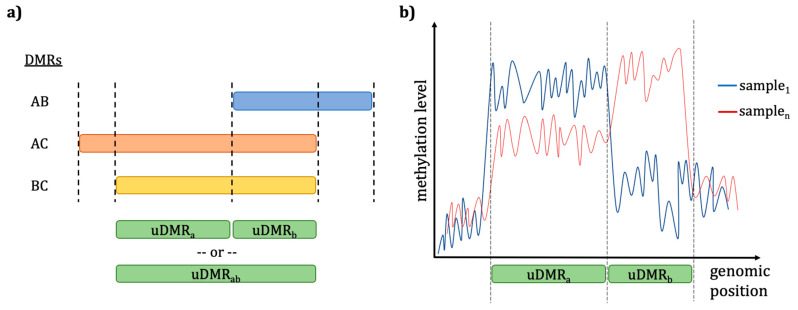
Average-over-region method with the DMRs input type. Overview to show (**a**) when differentially methylated regions (DMRs) arise from multiple pairwise comparisons between groups (e.g., AB, AC, BC) they are intersected to form distinct, nonoverlapping union DMRs (uDMR) according to a minimum fraction of supporting comparisons provided by the user (e.g., X = 0.5 in this sample). These uDMRs can be merged or taken as independent for further analysis. The resulting uDMRs are intersected with the methylated positions in (**b**) to derive average methylation levels in each sample for each region, which can then be carried forward as unique identifiers for EWAS. When only a single set of DMRs are provided to the pipeline, the regions are simply taken as is for the averaging process. This averaging process is repeated for all samples.

Cytosine methylation calls in all contexts (CG, CHG, and CHH) in bedGraph format as separated files (Figure S1) are used as methylated position (MP) input. Since each file represents a methylome per sample, these single bedGraph files are united with the bedtools unionbedg function [57] to generate a single methylation file with all samples as columns (Figure S2). In some cases, some positions are not covered by all sample methylomes, generating missing data (shown as NA), which may arise due to regions of low coverage sequencing. The pipeline can also be fed with differential methylation data in DMP/DMR bed format such as provided by the EpiDiverse DMR pipeline (Figure S3). The union of DMPs is simply intersected with the MPs with bedtools intersect to obtain individual methylation values per sample [57]. DMR input can be processed in two different ways—either (i) the MPs included in the region are analyzed or (ii) average methylation is calculated for all positions in that region for all samples, and regions are provided as identifiers (Figure 2). If no specific input parameter is indicated, the pipeline will automatically start the run with suitable models using the provided inputs (Table 1). 

The advantage of using MPs alone is that no prior assumption about pairwise comparisons or sample grouping has to be applied and that the full data are used. If there is good reason to believe that DMRs capture the hypervariable regions where DNA methylation differences are occurring, then it is an advantage to include them. This reduces the number of multiple tests of MPs/DMPs and can be based on meaningful a priori knowledge. On the other hand, when using DMPs and/or DMRs, the data size is reduced, resulting in lower running time for the EWAS pipeline itself. To decide which samples should be compared in a pairwise fashion to create DMPs or DMPs, assumptions need to be made that might bias the results and might not capture all the relevant information. 

#### 2.1.2. Available Models 

**Figure 3:**
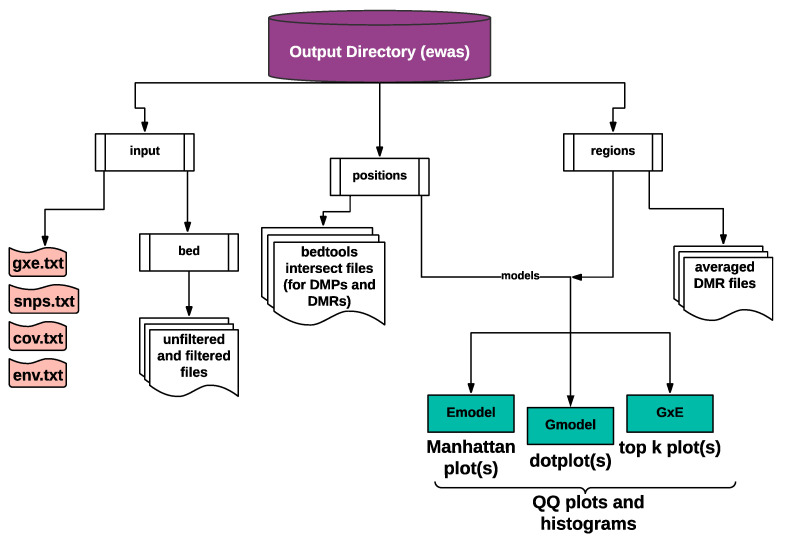
EpiDiverse EWAS pipeline output directory structure. EpiDiverse EWAS pipeline generates input directory as default and positions directory with methylation calls, DMPs, and regions directory DMRs. Input directory covers gxe.txt, snps.txt, cov.txt, and env.txt files and bed directory with merged bedGraph files as unfiltered and filtered and missing data estimated. Both positions and regions directories have three subdirectories for outputs and graphs with Emodel, Gmodel, and GxE names. Q–Q plots and histograms are produced with all models.

The models E, G, and GxE of the GEM tool suite are available in the EpiDiverse EWAS pipeline (Figure 1 and Figure 3). The Emodel is performed for detecting methylation markers associated with environmental parameters using linear regression *lm *(*M* ~ *E* + *cvrt*), with an *i*th vector from the methylation matrix (M), a jth vector from the environment matrix (E), and covariate(s) (cvrt) matrix, which is required for the grouping of samples, is used as a matrix-based iterative correlation (Figure S4) [50]. The output of Emodel is a list of potential epigenetic markers significantly correlated with a specific environmental factor. 

The Gmodel is used for detecting methylation markers associated with genotype data using a linear regression *lm (M~G + cvrt),* with an ith vector of methylation matrix (M), a jth vector of SNP genotype matrix (G), and covariate matrix. Gmodel creates a methQTL genome-wide map by performing a matrix-based iterative correlation between SNP and methylation matrices. The output of the Gmodel is a list of marker–SNP pairs, in which the SNP is coupled with the C of interest.

The GxE is used to reveal an association between genetic variation and environmental factors that may affect heritable or nonheritable DNA methylation levels, using again a linear regression *lm (M~G x E + cvrt),* in which environmental values are combined with the covariate file. The output of the GxE is a list of marker–SNP–env triplets in which the environment parameter is a factor divided into genotype groups (AA, AB, and BB) to explain the significant C of interest. This model’s output hypothesizes that the relationship between methylation and environment can be better understood by a division of genotype groups. As discussed in the “Removal of genetic variants that might be interpreted as significant epigenetic marks” Section the genetic variance may influence epigenetic variance via loss or gain of a methylation site and this can be addressed while intersecting different model outputs. 

#### 2.1.3. NA Filtering and Imputation with Methylation and SNP Datasets

Regardless of which markers are used with the EpiDiverse EWAS pipeline, a common problem has to be solved related to missing data. The pipeline unites methylation calls and DMP and DMR outputs from independently sequenced samples with the bedtools unionbedg function to derive the main methylation matrix. This unifying process leads to some positions having missing data (NAs). This problem may occur due to stochastic coverage variability or due to reference bias when a deletion affects a portion of the samples. Both cases can result in some loci being covered in some samples and not in others, causing incomplete datasets where NAs have to be excluded or estimated.

GEM replaces missing methylation values by calculating the global methylation for the rest of the sample and does not discard any positions with a high amount of missing data. As outlined above, the generic GEM method biases the calculation of FDR values (when the methylation data is divided by chromosome/scaffold, while the global methylation is calculated from the existing positions per sample, *p*-values vary for the same position; see “NA filtering and imputation with methylation and SNP datasets” for more details). Simply removing all positions where even one sample has missing data can be an alternative solution but can also reduce the total size of the dataset significantly in large cohorts. Instead, we implemented a strategy similar to that implemented in metilene [39], in which missing methylation values are imputed based on a beta distribution using the values of the remaining samples at the same position [58]. The reasoning is that if we are unable to provide real data to the model, then the next best thing is to provide estimated data that have the minimum possible impact on the model. Any significant associations that arise on markers with estimated data should therefore be driven by the samples for which we do have real data. Missing data imputation is also carried out for the SNP data using BEAGLE [40], based on a proportion of samples with missing data according to a user-defined threshold. 

#### 2.1.4. Text and Graphical Outputs 

Every model generates an unfiltered, filtered, and NA-imputed methylation file for each context (Figure 3). The Emodel output lists the model statistics for each epigenetic marker (rows) in the format “ID|beta|stats|pvalue|FDR,” where ID is for chromosome/scaffold names, beta is a beta coefficient in a linear model, stats is the t-statistics for the marker of interest, pvalue is the probabilistic score of an individual marker, and FDR are false discovery rate corrected *p*-values (*q*-values; Figure S5). The output of the Gmodel is a list of marker–SNP pairs, in which the SNP is the appropriate couple to explain the marker of interest. The only different column from the Emodel output is additional “snp” column next to the ID column; “ID|snp|beta|stats|pvalue|FDR” (Figure S6). The output of the GxE is a list of marker–SNP–env triplets where the environment is a factor divided into genotype groups (AA, AB, and BB) to explain the significant marker of interest, and output is otherwise the same as for Gmodel. 

The EWAS pipeline provides multiple output plots such as *p*-value Q–Q plots and histograms for all models (Figures S7 and S8), Manhattan plots for Emodel (Figure S9), sequence dot plots for Gmodel (Figure S10), and genotype interaction plots for the GxE (Figure S11). Each visualization is implemented using the ggplot2 package in R (https://github.com/tidyverse/ggplot2, accessed on 1 September 2019). 

### 2.2. Evaluation of the EpiDiverse EWAS Pipeline

Two published datasets of non-model plant species, namely, valley oak (*Quercus lobata*) with 58 samples [45] and Norway spruce (*Picea abies*) with 28 samples (derived from [59] and unpublished data), were used to test the reproducibility of the results and the performance of the EpiDiverse EWAS pipeline.

*Q. lobata* is a long-lived California endemic tree species with a ~730 Mbp genome [60]. Gugger et al. (2016) used RRBS to analyze whether climate is associated with variation in DNA methylation levels in 58 naturally occurring trees collected across climate gradients [45]. 

*P. abies* (Norway Spruce) is also a long-lived (conifer) tree species with a 20 Gbps draft genome [61]. Heer et al. (2018) analyzed eight *P. abies* trees in a targeted bisulfite sequencing approach, employing the SeqCapEpi Kit (NimbleGen). They sampled four trees (ortets) located in Bavaria, Germany, at ~1200 m above sea level (a.s.l.) and four clones that originated from those trees (ramets) planted at ~500 m a.s.l. [59]. In the present study, these data were extended with additional clones to test missing data management, replicate the results of the previous study, and determine the effects of input types. Those additional clones originated either from Germany or Sweden and were planted between 1970 and 1973 at several locations in Germany.

#### 2.2.1. Analysis of *Q. lobata* Dataset

In the original publication, it was suggested that single-methylation variants (SMVs), which are MPs are involved in response to the local environment and the acclimation to a climate in a long-lived tree species, valley oak [45]. The authors found 43 significant SMVs associated using several climatic variables. In total 38, 1, and 1 SMVs in CG, CHG, and CHH context were found to be associated with maximum temperature (tmax). A single CG–SMV associated with the minimum temperature (tmin) and single CHG and CHH SMVs associated with growing season growing degree days above 5 °C (GSDD5) were found to be significant. CG–SMVs showed stronger associations with climatic variables than other types of SMVs and SNPs. 

We used this dataset to test whether these findings could be replicated using the EpiDiverse EWAS pipeline (Table 1). When running Emodel, a total of 33 out of 38 tmax related CG–SMVs are shared, and the EWAS pipeline found 47 SMVs in total for this context (Table 2). Likewise, the single tmin-related CG–SMV and the tmax-related one (CHG, CHH), are shared. Results are also similar for GSDD5 in CG context and CWD in CG and CHH context. Hence, there is good agreement between the two analyses with the EWAS pipeline results containing the majority of the published results, while detecting a few more significant positions, in particular in the CHH context for tmin.

Open reading frames (ORFs) harboring significant MPs related to spatial and climatic variables were blasted against the NCBI nonredundant protein database and the closest hits were analyzed (Table S1). Significant MPs uniquely found by the EpiDiverse EWAS pipeline seem to be connected to relevant studies in the literature both for climatic and spatial variables (cf. Supplementary Section Blastx analysis with the *Q. lobata* dataset).

Some of the differences found between the two methods might be explained by differences in the estimation of missing data. Loci with more than 10% missing data were discarded from the previous analysis [45], and missing data were estimated by the EpiDiverse EWAS pipeline [58].

The previous study [45] used a multivariate method called RDA with a kinship matrix from methylation data. RDA is a forced classification method analogous to linear regression for cases that have multiple dependent variables (e.g., SMVs) and multiple independent variables (e.g., climate and spatial variables). RDA may not always be feasible with few variables, and this is especially true when there is a large proportion of unconstrained variation, i.e., the variation in the response matrix that is nonredundant with the variation in the explanatory matrix. Another thing that one cannot always be sure about which data to use to obtain the kinship matrix. In summary, the two methods perform similarly, with the EpiDiverse EWAS pipeline detecting a few more significantly correlated positions.

#### 2.2.2. Analysis of *P. abies* Dataset

Heer et al. (2018) hypothesized that the methylation percentage between clones from the two environments at a global level was similar and proposed that the methylation patterns remained largely stable during the life history of the trees [59].

We tested whether the sampling locations differ in terms of climatic variables. Due to a violation of normality using the Shapiro–Wilk test with 0.05 *p*-value, we carried out a nonparametric Wilcoxon test to compare locations between Goeppingen, Harsefeld, Neuhaus for clones apart from ramets, Bavarian forest national park for ortets, and Übersee for ramets in Germany. Precipitation (prcp) is significantly different between all sites (Figure S12 upper, left), whereas maximum (tmax) (Figure S12 upper, right), and minimum temperatures (lower) (Figure S12c) were found to be different only between some.

**Figure 4:**
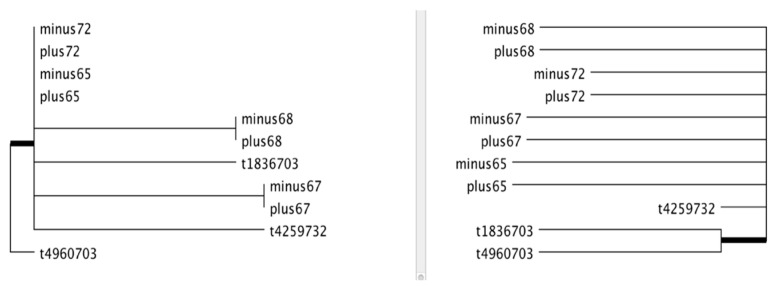
Coalescence analysis between the SNP and methylation data for the CG context. SNP (left) and averaged methylation call values (right) cluster comparison for the CG context. This comparison yielded a 72% topological score indicating a relatively high fraction of clades/branches present in both trees (cf. 3. Methods for details). The thick branches represent deviating topologies. Minus refers to ramets and plus to ortets. Cf. Figures S13–S18 for additional analyses.

Independently from the EpiDiverse EWAS pipeline, coalescence analysis between the SNP (genetic) and the methylation data (epigenetic) was performed to determine whether the samples are congruent and also to narrow down pairwise comparisons for DMP and DMR calling. Coalescence analysis with CG context showed a highly dissimilar pattern between the SNP and averaged methylation data per sample for the clones apart from ramets (Figure 4, please see Figures S13–S18 for other contexts and non-averaged methylation dendrograms). Since the similarity trees are based on genetic and epigenetic variance were dissimilar, three clustering approaches were employed to call DMPs/DMRs based on (i) trees’ locations, (ii) SNP clustering, and (iii) methylation call clustering (Figures S13–S18). In the previous study [59] ramet vs. ortet analysis was performed and revealed potentially interesting results that same ID ortets and ramets clustered together. Hence, this comparison was repeated here as one of the pairwise comparisons. An independent run outside of the EpiDiverse EWAS pipeline was conducted with an unsupervised method, kWIP [62], which found ramet and ortet pairs clustering (Figure S19). This outcome also confirms the same clustering with the previous study [59].

##### DMP/DMR Analysis Considerations Using Different Callers

No significant DMPs were found with *q* < 0.1 filterings by the EpiDiverse DMR pipeline using the default DMR caller metilene [63]. Hence, DMPs between “ramet vs. ortet” were compared with the output of other DMR callers, methylkit [64] and defiant (implemented in the EpiDiverse toolkit) [65], which yielded results with *q* < 0.1 (See Figure S20 and Supplemental Section “DMP/DMR analysis using different callers” for details). If one desires to use DMPs and/or DMRs as input instead of methylated positions (MPs), the alternative solution can be pairwise clustering to reveal differential positions and/or regions and a user has to define which groups to compare. It should be kept in mind that an unsupervised clustering may not always yield proper and distinct groups to achieve DMPs and/or DMRs.

##### Filtering Missing Data after Uniting Individual Methylomes

Bedtools unionbed function for unifying process creates some missing positions due to, e.g., varying coverage, resulting in some markers being covered in some samples and not in others, cf. Section 2.1.3 for more details (Table S2a,b).

Therefore, we filtered methylated positions’ data so that only those positions present across all samples remained. This led to only 7%, 7%, and 5.5% of data remaining in CG, CHG, and CHH contexts, respectively (Table S2c,d,e). To quantify the effect of missing data, we performed an iterative filtering analysis of 0.1 increments with filter_NA parameter using covariates based on the geographic location of trees, methylation, and SNP data (Figures S21–S32). Covariates only with the location of trees and combinations with it yielded the highest number of intersections between results. SNP and methylation-based covariates showed no significant outputs.

##### The Intersection of Positions with All Inputs and Models for the CG Context

In order not to bias the data via NA correction, a zero-tolerance missing data threshold was used in all subsequent *P. abies* analyses.

**Figure 5:**
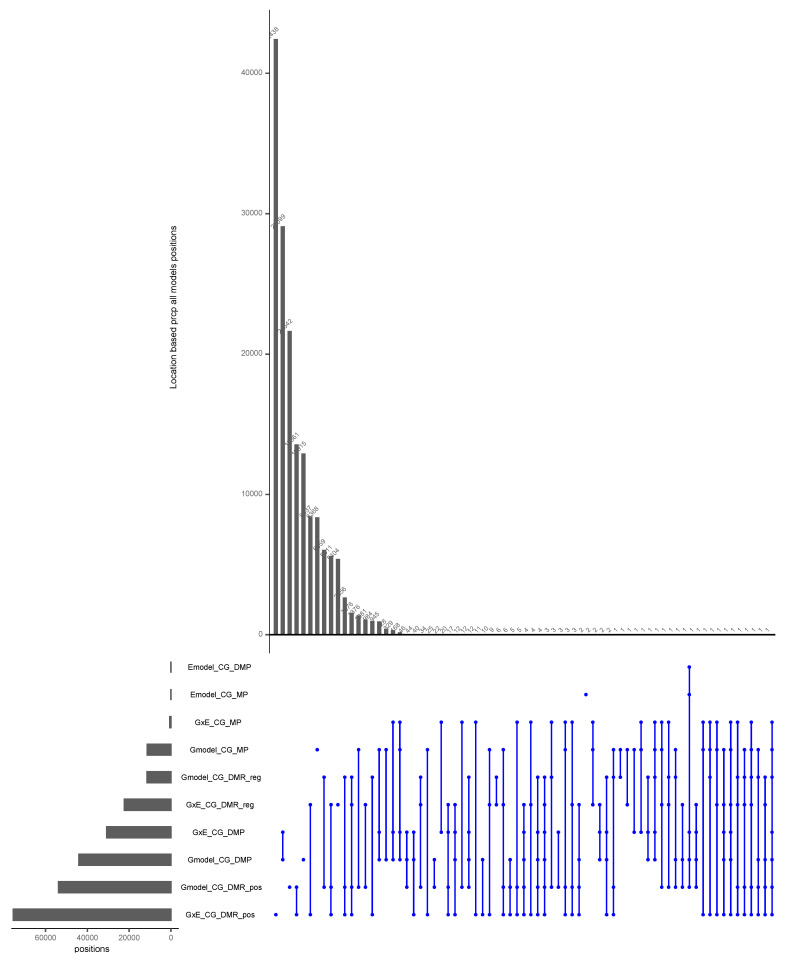
Upset plot of significant positions for all models with CG context using locations of trees as covariates with precipitation environmental data. Vertical lines refer to shared terms between classes on the left side. A maximum shared number of terms is between G and GxE models with DMP input type. Overall, 39% of the terms are shared and 61% are unique to single inputs. The highest number of unique elements are found for GxE DMR input with 42,438 terms, and the lowest is with two terms for the Emodel CG MP input.

It was shown that gene body CG methylation is relatively stable across seasons [66]. Hence, for this test study, the EWAS run with all models was performed in the CG context only. Significant positions in all model outputs were intersected for location-based clustering using precipitation environment and CG context data with the UpsetR R package [67] for all input types (Figure 5). We selected precipitation for this study because it showed significant differences between all sample locations.

In summary, G and GxE models with DMPs as input is the combination that yields the highest number of significant positions. The maximum number of groups that share a position is seven (check the vertical line on the far right in Figure 5). Moreover, 20, 182, 9713, 37,026, and 77,405 terms are, respectively, shared by five, four, three, two, and single groups. It makes sense to test several inputs and models for higher sensitivity.

Depending on the input type and model, the output in terms of significant positions varies considerably.

##### Removal of Genetic Variants That Might Be Interpreted as Significant Epigenetic Marks

To determine potentially problematic overlap between genetic and epigenetic variation we intersected all models. Only one position was found to be shared between Emodel MP, G & GxE models DMP input, “MA_160146:1616-1617”, i.e., position 1616/17 on *P. abies* contig MA_160146 (Figure S33). A total of 16 SNPs were found to be correlated with this CG, 16 for the GxE and one for the Gmodel, 17 SNPs are in common between G and GxE models, and only five of them are on CG bases. Additionally, those 17 shared SNPs were intersected with the output of the EWAS runs and it was observed that the G and GxE DMRs and averaged outputs have an intersection with these 17 SNPs. In conclusion, the optimal intervention as post hoc analysis should be excluding the intersected CG identifiers from Emodel, in other words, shared positions between Emodel and Gmodel/GxE should be removed from the Emodel output if the aim is to discover epigenetic associations that arise purely due to environmental factors (the same is valid for Gmodel when obtaining solely genetic-related identifiers). Please check the “Removal of genetic variants that might be interpreted as significant epigenetic marks” chapter in Supplementary for more details.

##### Emodel Output Gene Ontology (GO) Analysis

To determine environmentally associated positions, we conducted an Emodel analysis, integrating all available climatic data and all contexts. Current climatic datasets through the CHELSA database (Climatologies at high resolution for the earth’s land surface areas) [68] are maximum, minimum temperature, and precipitation. Since on the level of individual positions, there is scarce overlap between all input types (MPs, DMPs, and DMRs), we decided to analyze the results on the level of Gene Ontology (GO) level (Figures S34–S36) to check concordance in different model outputs and found enriched GO terms for significant cytosines. Such analyses can be useful to reduce noise and to determine the conserved overlap of datasets [69].

To determine whether GO terms are concordant between different input types we concentrated on the usually most meaningful biological process (BP) ontology and found that 47% of terms overlap with the previous study in which only ortets vs. ramets were analyzed [59] (Figure S34). The highest number of significant terms (26) in the Emodel analysis resulted from MP input, CHH context, and tmin climatic data (Figure S37).

##### CG Context G and GxE GO Analysis

Since the G and GxE models are computationally intensive, we ran them only on precipitation data, which was found to be significantly different between all locations (Figure S12), and in CG context, since it was shown that CG methylation is relatively stable seasonally [66,70]. G and GxE GO outputs (Figures S38–S41) were intersected to distinguish the effect of climatic data vs. SNP data. The filtered output results (*q* < 0.05) for CG context with Gmodel show that all four input types yield significant terms (Table S3). In total, 69% of BP terms are shared and only 31% are unique to single inputs (Figure S38).

Enriched BP terms that are found with G and GxE models include, e.g., “monoterpene,” “metabolic organic biopolymer,” and “phenylpropanoid.” The term “response to temperature stimulus” was found with Gmodel DMP and GxE MP inputs. Other prominent terms that might be related to precipitation were “water transport” (all inputs of GxE, and DMP input with Gmodel) and “water homeostasis” (Gmodel with DMR average method).

The *P. abies* dataset analysis showed that most of the GO terms between different models are shared and only a few are unique to a given model. The term “phenylpropanoid metabolic process” is shared between all outputs except for Gmodel MPs and DMPs. Phenolic extracts in *P. abies* were reported to exhibit antifungal [71], antibacterial [72], and antioxidative activity [73]. Additionally, phenolic compound-related terms were found significantly in higher numbers with needles of damaged trees [74]. It was shown by other studies that the colonization of trees by various bark beetle species was related to the released number of monoterpenes [75]. Terms found with “water transfer” seem to exist in studies for drought resistance [76,77].

**Figure 6:**
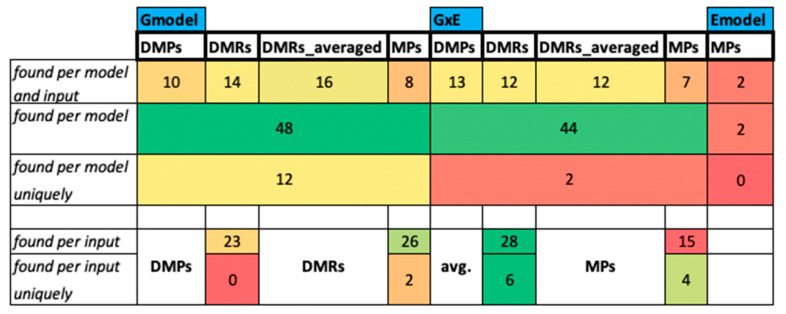
Subset of BP GO terms related to “water”, “root”, “shoot”, and “defense” per input type under three models (G, GxE, and E), in GC context for precipitation. Several BP GO terms matching “water”, “root”, “shoot”, or “defense” are shown per model and input type. Cells are colored from green = high to red = low.

In summary, Gmodel with averaged DMRs as input is the combination that yields the highest number of terms. However, it should be noted that the GxE also found a (low) number of unique terms and that DMRs and MPs as inputs also yielded unique terms. Hence, for higher sensitivity or a consensus approach, it makes sense to test several input types and models (Figure 6), in particular since results also vary per context, as shown for the Emodel study (Figure S37).

An assumption is needed to decide which groups to compare to derive DMPs and DMRs, but no assumption is required with MP input type. Covariates are used for grouping samples, and the user may prefer multiple of them. Computation time may take longer while using the MP type input, but it should be noted that using methylated positions are suggested to be used as default in case a user does not have differential methylation values, and it would be advantageous to use the whole (unbiased) methylation data. Additionally, a user should consider the time that will be spent to obtain the differential methylation before executing EWAS. Therefore, we recommend MP input for a start and in absence of concrete ideas (such as ramet vs. ortet in the *P. abies* dataset) for pairwise groupings. If those are present, averaged DMRs might be preferable based on the number of terms that can be derived from them (Figure 6).

### 2.3. Conclusions

The EpiDiverse EWAS pipeline allows the analysis of MPs and differential methylation data. It presents logical missing data imputation with beta distribution and produces multiple graphs with each model in the GEM package to help the user to observe results better. We reanalyzed data published previously [45] and found a significant amount of overlap in terms of significant MPs related to spatial and climatic variables.

In terms of the *Q. lobata* dataset [45], we found that nearly all significant C’s could be reproduced, although the underlying statistical methods differ. Missing data estimation as implemented in the EpiDiverse EWAS pipeline suggests that beta distribution is a robust and accurate choice for approximation, as inferred from the significant amount of overlap. Most importantly, nearly all of the unique C’s only found by the EWAS pipeline seem to have meaningful associations with spatial and climate variables in the literature.

We used the *P. abies* dataset to determine the overlap between different GEM models and input types. We found that the choice of model and input depends on the user’s research question. G and GxE models detected more significant GO terms, compared to the Emodel terms in GC context (Figure 6, and averaged DMRs are superior to the other input types in terms of how many terms can be detected. As a hierarchical controlled vocabulary, gene ontology helps to group meaningful biological functions that might be missed in individual gene descriptions. Different genes related to the same biological function may have GO terms in common. Finding most of the GO terms overlapping between different analyses shows a large part of the findings of these analyses are shared on the level of the ontological vocabulary and its underlying functionality, e.g., the biological process enacted. Yet, Emodel found the highest number of terms in the CHH context (Figure S36). Most of the detected GO terms overlapped between different models, inputs, and contexts, suggesting robust results regardless of the model and, to some extent, input type. However, most models and input type combinations yield a certain fraction of uniquely found terms, suggesting that a consensus approach (using several models and input types and using their overlap) might make sense (Figure 6).

In terms of input filtering, we found that *p*-value-filtered metilene DMPs do not lead to severely different results from using *q*-value-filtered methylkit or defiant DMPs. Unsupervised clustering using kWIP to derive groups for DMP and DMR analysis was found to be a potential replacement for a priori grouping.

In summary, we present the EpiDiverse EWAS pipeline as a versatile tool to perform plant EWAS analyses, either using the output of the other EpiDiverse pipelines or custom data.

## 3. Materials and Methods

### 3.1. The EpiDiverse EWAS Pipeline

The EpiDiverse EWAS pipeline is available on GitHub (https://github.com/EpiDiverse/ewas/tree/master, accessed on 1 March 2021). The pipeline was set up using Nextflow 20.07.1 revisions [54] and is based on the GEM R package [50].

### 3.2. Analysis of Q. lobata Data

As described in the original publication, mature leaves from *Q. lobata* were sampled at each of 58 locations spread along the foothills of the Coastal and Sierra Nevada ranges [45]. Positions with more than 10% missing data, less than 10X coverage, and a 10% range of variation (the difference between the maximum and minimum methylation per position) were filtered out. The authors considered four different climate variables and integrated amount of water availability or stress, considering evapotranspiration, basin hydrology, and rainfall, mean minimum temperature of the coldest month (tmin), mean maximum temperature of the warmest month (tmax), and growing season growing degree days above 5°C (GSDD5). A multivariate method called redundancy analysis (RDA) was employed to test the variation explained by SMVs and SNPs, and positions were filtered with multiple testing (*q* < 0.1).

To mirror these analyses, the EWAS pipeline run was performed with 10× coverage, *q* value < 0.1, with a maximum of 10% of missing data, and different standard deviation values per position (0.028, 0.0176, and 0.0197 for CG, CHG, and CHH, respectively) to replicate the results in the previously published study [45]. These parameters produced the same amount of data produced in Gugger et al. (2016) with negligible differences in terms of FDR calculation (Figure S41).

### 3.3. Analysis of P. abies Data

The *P. abies* dataset was chosen to perform a comprehensive test for measuring the performance and parameters of the EWAS pipeline. In total, 28 samples were used, composed of four original trees or ortets (ID = 65, 67, 68, 72), four clones that originated from those ortets or ramets (ID = 65, 67, 68, 72), and 20 clone trees originated from three trees (ID = 4259732, 4960703, 186370), two located in Germany and one in Sweden. Clones from these trees were planted by the Northwest German Forest Research Institute as part of their project “fit for clim” (https://www.fitforclim.de/, accessed on 1 March 2021) with varying climatic conditions in Germany, namely, Neuhaus (with unique numbers or Mitte/middle, oben/up, unten/low extensions based on the position in the tree they were sampled from), Göppingen (G extension), and Harsefeld (H extension) (Figure S42).

The EpiDiverse WGBS pipeline with the segemehl standalone tool [66] was used for methylation calling with the options --noDedup, --SE, and --unique, using the high confidence gene set as reference [52]. Overall, 15 to 85 million reads per sample were left after trimming, yielding a coverage of 8- to 26-fold (Table S4). Although the duplicate ratio was quite high due to the linear relationship of sequencing depth and duplicate level, this is probably not due to PCR bias (see Supplement, Ratio of PCR Duplicates for *P. abies* Datasets, Figure S43).

The EpiDiverse DMR pipeline with the metilene software was used to call DMRs with default settings and --sig 0.1 and --diff 20 for DMPs. The EpiDiverse SNP pipeline was used with the --variants parameter to derive SNP variants per sample as separated .vcf files. All files were compressed with bgzip, indexed, and finally merged and filtered to keep variants that have been successfully genotyped in 100% of individuals, a minimum quality score of 30, and a minor allele count of three. This final file was used with the –SNPs parameter for the EWAS pipeline run. The EpiDiverse EWAS pipeline takes individual variant files to merge and filter them.

The EpiDiverse EWAS pipeline run was conducted separately for each methylation context while disabling the other two, e.g., --noCHH --noCHG parameters used for a CG run, --distance parameter was set to 2000, and --coverage to 5. G and GxE models were run in 10 separate runs, and all positions with noninfinite t statistics were discarded. Separate outputs were merged, and FDR calculation was carried out.

Hierarchical clustering (HC) with the Euclidean distance method with ctc package in R was performed on genetic variability (SNPs) and on epigenetic variability (methylated positions). The idea was that if there is no difference between the two resulting topologies, either of them might be used to derive groups. Coalescence analysis was performed to check for commonalities/differences of the topologies, and methylation calls and SNP HC graphs were compared via the compare2trees standalone tool [67]. The SNP tree is composed of 11 samples, including the four ramets, the four ortets (ramets and ortets are assumed to be genetically identical), and the three new clones. The methylation data comprises 28 samples: 4 from the ramets, 4 from the ortets, and 20 from the new clones. Two versions of the methylation tree were used, with all samples separate, and using averaged methylation per position for the same tree ID located in different locations. Branch thickness in the result of the compare2trees software is used to show the topological score, which is the percentage fraction of clades/branches that are present in the actual tree that is also present in the estimated tree; thicker branches refer to a lower score (Figure 4 and Figures S13–S18). The kWIP tool was used to cluster methylation data for four ramets and ortets.

GO bias analyses were performed with the GOSTAT pipeline while intersecting the results from the EWAS pipeline with GO term annotations [68].

## Figures and Tables

**Table 1:** Required inputs and file formats for the EpiDiverse EWAS pipeline. All possible input types and requirements for different models to run the epigenome-wide association studies (EWAS) pipeline. Tab-separated “sample sheet” file and methylated positions (MPs) input are required for all runs, differentially methylated positions (DMPs), and differentially methylated regions (DMRs) are needed if users would like to run the pipeline with these inputs. Genetic variants are necessary for G and GxE models.

Input	Description	File(s) Formats	Required for Which Runs?	Required for Which Model?
sample sheet	Sample list, which includes sample names as key variables, single environment/phenotype data, and covariate(s).	txt	Required for all runs	Required for all models
MPs	Context-specific methylation calls per sample.	bedGraph	Required for all runs	Required for all models
DMPs	Context-specific differentially methylated positions.	bed	Required to run the pipeline with DMPs	Allowed for all models
DMRs	Context-specific differentially methylated regions.	bed	Required to run the pipeline with DMRs	Allowed for all models
Genetic variants	Genetic markers either in single or multisample formats.	vcf or vcf.gz	Required to run the G and GxE models	Required for G and GxE models

**Table 2:** Comparison of EpiDiverse EWAS Emodel output for valley oak with the published data.

CG	tmax ^1^	tmin ^2^	GSDD5 ^3^	CWD ^4^
Gugger et al., 2016	38	1	0	0
EpiDiverse EWAS pipeline	47	2	0	0
shared amount	33	1	not applicable	not applicable
Shared % based on Gugger et al., 2016	86.42%	100%	100%	100%
**CHG**				
Gugger et al., 2016	1	0	1	0
EpiDiverse EWAS pipeline	1	0	0	1
shared amount	1	Not applicable	0	0
Shared % based on Gugger et al., 2016	100%	100%	0%	0%
**CHH**				
Gugger et al., 2016	1	0	1	0
EpiDiverse EWAS pipeline	3	16	0	0
shared amount	1	not applicable	0	not applicable
Shared % based on Gugger et al., 2016	100%	0%	0%	100%

^1^ tmax: maximum temperature, ^2^ tmin: minimum temperature, ^3^ CWD: (an integrated measure of water availability or stress considering rainfall, evapotranspiration, and basin hydrology), and ^4^ GSDD5 (growing season growing degree days above 5 °C).

## Data Availability

The pipeline is available at https://github.com/EpiDiverse/ewas/tree/master, accessed on 1 March 2021. BAM Files containing the mapped reads for ramet and ortets are available at European Nucleotide Archive (ENA, www.ebi.ac.uk/ena/, accessed on 1 September 2019) under the project PRJEB26494, raw read fastq accessions under ERR2591764:ERR2591771. Mapped reads for the other 20 clone trees are under the project PRJNA703787, raw read fastq accessions are under the SRA run accessions SRR13760855: SRR13760874.

## Supplementary Materials

The following are available online at https://www.mdpi.com/article/10.3390/epigenomes5020012/s1, Figure S1: WGBS output directory and a bedGraph formatted dataset, Figure S2: Methylome input and the final file after bedtools unionbedg process, Figure S3: DMPs or DMRs output directory and a bed formatted dataset, Figure S4: Required inputs to run EpiDiverse EWAS pipeline with different models, Figure S5: The output of the Emodel, Figure S6: The output of the Gmodel, Figure S7: Q–Q plots, Figure S8: Histogram plots, Figure S9: Manhattan plots, Figure S10: Sequence dot plots, Figure S11: Top significant k-plots, Figure S12: Nonparametric Wilcoxon test to compare climatic datasets for locations of trees, Figure S13: Coalescence analysis with SNP and averaged methylation data for the CG context, Figure S14: Coalescence analysis with SNP and not averaged methylation data for the CG context, Figure S15: Coalescence analysis with SNP and averaged methylation data for the CHG context, Figure S16: Coalescence analysis with SNP and not averaged methylation data for the CHG context, Figure S17: Coalescence analysis with SNP and averaged methylation data for the CHH context, Figure S18: Coalescence analysis with SNP and not averaged methylation data for the CHH context, Figure S19: fastq raw files HC with k32 performed by kWIP software, Figure S20: Intersection of significant C’s with *p*- and *q*-values on gene level for methylkit, metilene, and defiant DMR callers., Figure S21: Intersection of outputs with different filter_NA values for MPs input using all covariates, Figure S22: Intersection of outputs with different filter_NA values for DMPs input using all covariates, Figure S23: Intersection of outputs with different filter_NA values for DMRs input using all covariates, Figure S24: Intersection of outputs with different filter_NA values for MPs input using only location-methylation-based covariates, Figure S25: Intersection of outputs with different filter_NA values for DMPs input using only location-methylation-based covariates, Figure S26: Intersection of outputs with different filter_NA values for MPs input using only location-SNP-based covariates, Figure S27: Intersection of outputs with different filter_NA values for DMPs input using only location-SNP-based covariates, Figure S28: Intersection of outputs with different filter_NA values for DMRs input using only location-SNP-based covariates, Figure S29: Intersection of outputs with different filter_NA values for MPs input using only SNP-methylation-based covariates, Figure S30: Intersection of outputs with different filter_NA values for MPs input using only location-based covariates, Figure S31: Intersection of outputs with different filter_NA values for DMPs input using only location-based covariates, Figure S32: Intersection of outputs with different filter_NA values for DMRs input using only location-based covariates, Figure S33: Intersection of shared SNPs and significant common markers between G and GxE models, Figure S34: Intersection of significant BP GO terms between location-based Emodel output and a previous study [5] with UpsetR package, Figure S35: Intersection of significant MF GO terms between location-based Emodel output and a previous study with UpsetR package, Figure S36: Intersection of significant CC GO terms between location-based Emodel output and a previous study with UpsetR package, Figure S37: Highlighted GO terms based on Emodel, Figure S38: Intersection of significant BP GO terms between all models, only CG context and precipitation data for location-based clustering, and a previous study with UpsetR package, Figure S39: Intersection of significant MF GO terms between all models, only CG context and precipitation data for location-based clustering, and a previous study with UpsetR package, Figure S40: Intersection of significant CC GO terms between all models, only CG context and precipitation data for location-based clustering, and a previous study with UpsetR package, Figure S41: Gugger et al. (2016) methylation and climatic data processing and analysis by the EpiDiverse EWAS pipeline, Figure S42: Location of *P. abies* trees (a), additional clone information (b), and grouping of trees (c), Figure S43: PCR duplicate analysis, Table S1: *Q. lobata* blastx analysis, Table S2: Missing data estimation of EpiDiverse EWAS pipeline (a) and GEM R package (b) [9]. Missing data statistics for P. abies dataset with CG (c), CHG (d), and CHH (e) contexts, Table S3: EWAS output and GO statistics, Table S4: Statistics of additional *P. abies* samples.
