# Supplementary material for: The EpiDiverse Plant Epigenome-Wide Association Studies (EWAS) Pipeline
Source: Epigenomes. 2021 May 4;5(2):12. doi: 10.3390/epigenomes5020012 (PMC8594691; doi:10.3390/epigenomes5020012)
Supplement: Supplementary file 1 [file epigenomes-05-00012-s001.zip › epigenomes-1165117-Supplementary.pdf]

epigenomes

Supplementary information for

The EpiDiverse plant Epigenome-Wide Association Studies (EWAS) pipeline

S. Nilay Can et al.

Supplementary Figures and Table

Table of contents

Figure S1: WGBS output directory and a bedGraph formatted dataset.
Figure S2: Methylome input and the final file after bedtools unionbedg process.
Figure S3: DMPs or DMRs output directory and a bed formatted dataset.
Figure S4: Required inputs to run EpiDiverse EWAS pipeline with different models.
Figure S5: The output of the Emodel.
Figure S6: The output of the Gmodel.
Figure S7: QQ plots.
Figure S8: Histogram plots.
Figure S9: Manhattan plots.
Figure S10: Sequence dot plots.
Figure S11: Top significant k-plots.
Figure S12: Non- parametric Wilcoxon test to compare climatic datasets for locations of trees.
Figure S13: Coalescence analysis with SNP and averaged methylation data for the CG context.
Figure S14: Coalescence analysis with SNP and not averaged methylation data for the CG context.
Figure S15: Coalescence analysis with SNP and averaged methylation data for the CHG context.
Figure S16: Coalescence analysis with SNP and not averaged methylation data for the CHG context.
Figure S17: Coalescence analysis with SNP and averaged methylation data for the CHH context.
Figure S18: Coalescence analysis with SNP and not averaged methylation data for the CHH context.
Figure S19: fastq raw files HC with k32 done by kWIP software.
Figure S20: Intersection of significant Cs with p and q-values on gene-level for methylkit, metilene, and defiant DMR callers.
Figure S21: Intersection of outputs with different filter_NA values for MPs input using all covariates.
Figure S22: Intersection of outputs with different filter_NA values for DMPs input using all covariates.
Figure S23: Intersection of outputs with different filter_NA values for DMRs input using all covariates.
Figure S24: Intersection of outputs with different filter_NA values for MPs input using only location-methylation-based covariates.
Figure S25: Intersection of outputs with different filter_NA values for DMPs input using only location-methylation-based covariates.
Figure S26: Intersection of outputs with different filter_NA values for MPs input using only location-SNP-based covariates.
Figure S27: Intersection of outputs with different filter_NA values for DMPs input using only location-SNP-based covariates.
Figure S28: Intersection of outputs with different filter_NA values for DMRs input using only location-SNP-based covariates.
Figure S29: Intersection of outputs with different filter_NA values for MPs input using only SNP-methylation-based covariates.
Figure S30: Intersection of outputs with different filter_NA values for MPs input using only location-based covariates.
Figure S31: Intersection of outputs with different filter_NA values for DMPs input using only location-based covariates.
Figure S32: Intersection of outputs with different filter_NA values for DMRs input using only location-based covariates.
Figure S33: Intersection of shared SNPs and significant common markers between G and GxE models.
Figure S34: Intersection of significant BP GO terms between location-based Emodel output and a previous study [5] with UpsetR package.

Figure S35: Intersection of significant MF GO terms between location-based Emodel output and a previous study with UpsetR package.
Figure S36: Intersection of significant CC GO terms between location-based Emodel output and a previous study with UpsetR package.
Figure S37: Highlighted GO terms based on Emodel
Figure S38: Intersection of significant BP GO terms between all models, only CG context and precipitation data for location-based clustering, and a previous study with UpsetR package.
Figure S39: Intersection of significant MF GO terms between all models, only CG context and precipitation data for location-based clustering, and a previous study with UpsetR package.
Figure S40: Intersection of significant CC GO terms between all models, only CG context and precipitation data for location-based clustering, and a previous study with UpsetR package.
Figure S41: Gugger et al., 2016 methylation and climatic data processing and analysis by the EpiDiverse EWAS pipeline.
Figure S42: Location of *P. abies* trees (a), additional clone information (b), and grouping of trees (c).
Figure S43: PCR duplicate analysis.

Table S1: *Q. lobata* blastx analysis
Table S2: Missing data estimation of EpiDiverse EWAS pipeline (a) and GEM R package (b) [9]. Missing data statistics for P. abies dataset with CG (c), CHG (d), and CHH (e) contexts.
Table S3: EWAS output and GO statistics.
Table S4: Statistics of additional *P. abies* samples.

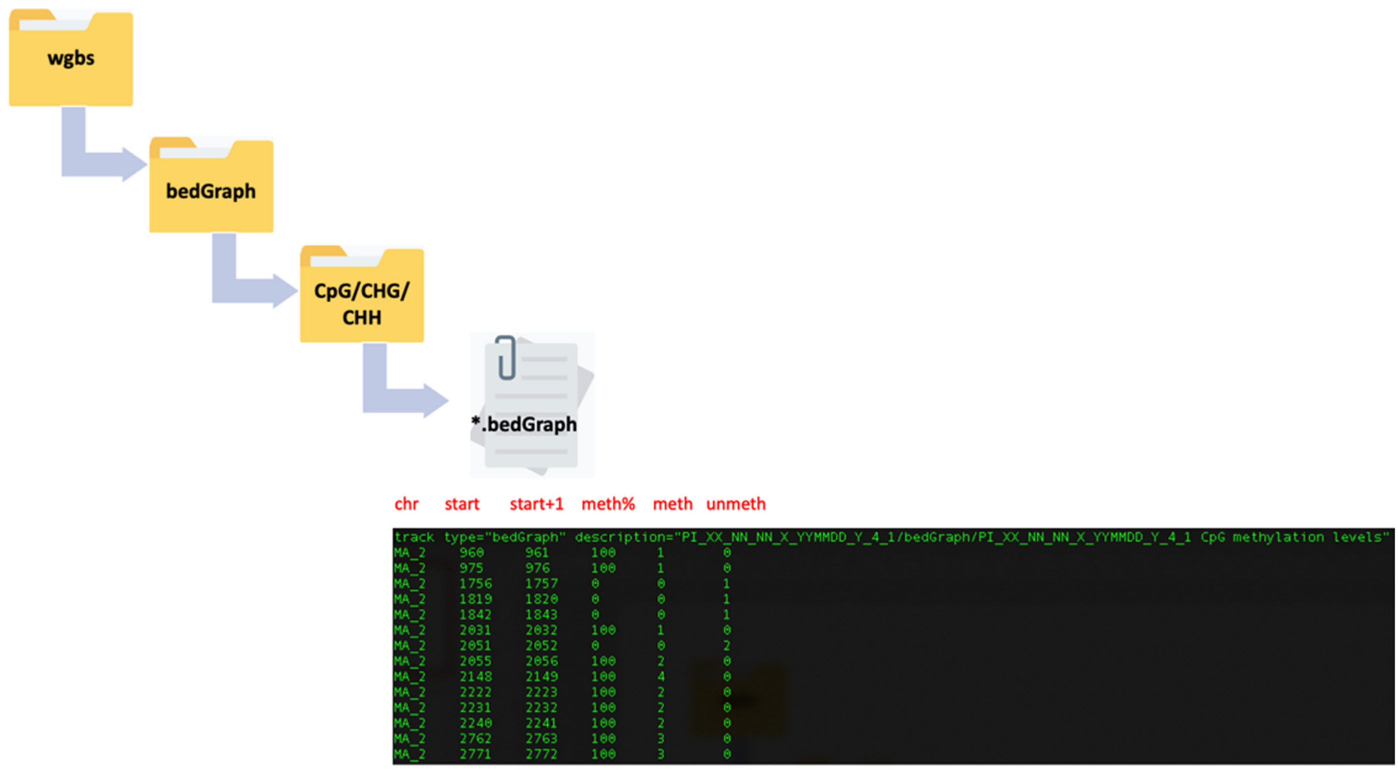

Figure S1**: WGBS output directory and a bedGraph formatted dataset.**

BedGraph files have a typical MethylDackel (https://github.com/dpryan79/MethylDackel) output format, and the user has to provide files in this format. Input files can be filtered by a coverage parameter (sum of fifth and sixth columns).

track type="bedGraph" description="sample1 CpG methylation levels"					
**#chrom**	**start**	**end**	**meth%**	**meth**	**unmeth**
**MA_10**	372	373	83	5	1
**MA_10**	449	450	33	2	1
**MA_10**	490	491	57	11	8

Methylome of sample1 (sample1.bedGraph)

track type="bedGraph" description="sample2 CpG methylation levels"					
**#chrom**	**start**	**end**	**meth%**	**meth**	**unmeth**
**MA_10**	449	450	0	0	1
**MA_10**	490	491	100	1	0

Methylome of sample2 (sample2.bedGraph)

track type="bedGraph" description="sample2 CpG methylation levels"					
**#chrom**	**start**	**end**	**meth%**	**meth**	**unmeth**
**MA_10**	490	491	42	3	4

Methylome of sample3 (sample3.bedGraph)

Bedtools unionbedg

**chrom**	**start**	**end**	**sample1**	**sample2**	**sample3**
**MA_10**	371	372	NA	NA	NA
**MA_10**	372	373	0.83	NA	NA
**MA_10**	449	450	0.33	0	NA
**MA_10**	490	491	0.57	1	0.42
**MA_10**	491	492	NA	NA	NA

Methylome of all samples

Figure S2: **Methylome input and the final file after bedtools unionbedg process.**
Each file represents the methylome of a sample and these single bedgraph files need to be united to generate the main methylation file. "Bedtools unionbedg [1] function with –filler NA -i" parameter to fill missing methylation with "NA" (not available) is used for that uniting purpose and the resulting file represents the methylation input file. The user should have this format of bedGraph files to be able to run the pipeline.

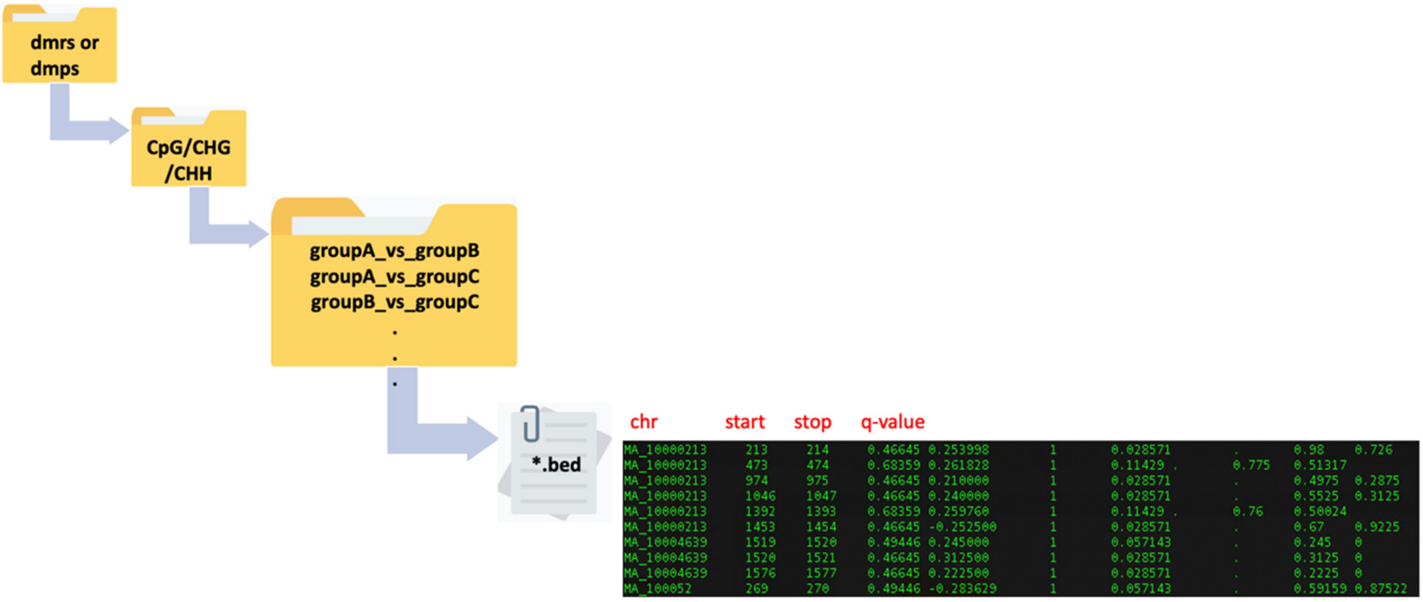

Figure S3: **DMPs or DMRs output directory and a bed formatted dataset.**

Bed files can be filtered by a "filter_FDR" parameter based on the fourth column. This is a typical output of metilene DMR caller [2] which is a default methylation caller used in the implementation of the EpiDiverse DMR pipeline, and columns are neglected after the fourth one, so it is enough for a user to have a file with chr, start, stop, and q-value information in order.

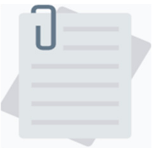

Methylation data

methylation.txt

chr	start	stop	meth $sample_1$	meth $sample_2$	meth $sample_3$	meth $sample_4$	meth $sample_5$	meth $sample_6$	meth $sample_7$
chr_3	124	125	0.45	0.57	0.78	1.00	0.00	0.00	0.00
chr_2	345	346	0.33	0.35	0.38	0.42	0.45	0.54	0.90
……									
chr_n	12	13	1.00	1.00	1.00	0.00	0.00	0.03	0.04

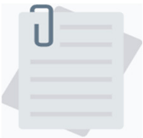

SNP genotype data

snp.txt

##SNPs	$sample_1$	$sample_2$	$sample_3$	$sample_4$	$sample_5$	$sample_6$	$sample_7$
SNP_1	1	1	2	2	3	3	3
SNP_2	1	1	2	2	2	2	3
……							
SNP_n	1	2	2	3	3	3	3

1: major homozygote (AA)
2: heterozygote (AB)
3: minor homozygote (BB)

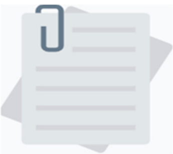

samples.tsv

#sample names	Environment value (single)	Covariate(s) can be multiple columns	cov_2	cov_3	cov_4
$sample_1$	108.65	1	1	1	1
$sample_2$	112	1	2	2	1
$sample_3$	156	2	2	2	1
$sample_4$	200	2	2	3	1
$sample_5$	102.43	3	2	4	2
$sample_6$	103.78	3	3	5	2
$sample_7$	108	4	3	6	2

Figure S4: **Required inputs to run EpiDiverse EWAS pipeline with different models.**

Methylation file is generated by the pipeline using single bedGraph files per sample. SNP genotype matrix is also generated by the pipeline either from multi-sample vcf.gz file or single .vcf files. The only user-provided files are the tab-separated samples.tsv file if other files are generated by EpiDiverse pipelines which carry environment and covariate information. The user can provide only a single environment data regardless of integer or float values. Covariate data should come after the environment data and they can be single or multiple. Values used for covariates are nominal and only for grouping samples depending on the user's needs.

ID	beta	stats	p-value	FDR
MA_14489:23538-23539	0.00309592814027513	5.58260174194547	8.32325006781751e-06	0.0495889
MA_10426464:2219-2220	0.000972548433628362	5.58295616551301	8.31571577095554e-06	0.0495889
MA_7645347:976-977	0.00075589779374216	5.58504358017725	8.27148147560822e-06	0.0495087
MA_3767:29361-29362	0.00037794889687108	5.58504358017725	8.27148147560822e-06	0.0495087
MA_10427647:1309-1310	0.00037794889687108	5.58504358017725	8.27148147560822e-06	0.0495087
MA_10394370:9851-9852	0.00105494852067155	5.58684814886495	8.23343278630598e-06	0.0495087
MA_666458:1936-1937	0.00188363311830361	5.59002223392068	8.16693731763542e-06	0.0493398
MA_10431464:4467-4468	0.00118021215245349	5.59251715824307	8.11505139162908e-06	0.0491411
MA_10430912:11319-11320	0.00104056617820108	5.59338279592362	8.09712709235094e-06	0.0491411
MA_41176:14201-14202	0.00135151709301068	5.59391786854644	8.08606765998442e-06	0.0491411
MA_20503:50086-50087	0.00084656885720403	5.59741918832477	8.01407490777187e-06	0.048873
MA_10435640:15749-15750	0.00172947615747984	5.60148922684603	7.93120278177804e-06	0.048482

Figure S5: **The output of the Emodel.**

This file has "ID|beta|stats|pvalue|FDR" columns where cpg is for significant chr/scaffold names, beta is a beta coefficient in a linear model, stats is the t-statistics for the C in interest, pvalue is the probabilistic score of a C and FDR is corrected p-values, in other words, q-values.

ID	SNP	beta	stats	p-value	FDR
MA_395749:465-466	MA_8292402:2532-2533	0.0583676366217176	4.64000229971677	9.47326036380823e-05	0.0499999751858288
MA_395749:465-466	MA_10437042:17374-17375	0.0583676366217175	4.64000229971677	9.47326036380823e-05	0.0499999751858288
MA_395749:465-466	MA_10435682:17404-17405	-0.0583676366217175	-4.64000229971677	9.47326036380833e-05	0.0499999751858288
MA_395749:465-466	MA_10435682:17404-17405	0.0583676366217175	4.64000229971677	9.47326036380833e-05	0.0499999751858288
MA_10302644:7598-7599	MA_45157:14959-14960	0.0828140703517588	4.64000159732888	9.47327761869289e-05	0.0500000583090976
MA_10302644:7598-7599	MA_45157:14974-14975	0.0828140703517588	4.64000159732888	9.47327761869289e-05	0.0500000583090976
MA_10426291:3069-3070	MA_89747:15112-15113	-0.0565140073081608	-4.64000106859483	9.47329060761196e-05	0.0500001228906404
MA_10372599:1442-1443	MA_15175:36373-36374	0.479535192563081	4.63999719636802	9.47338573356091e-05	0.0500006209922063
MA_14988:11612-11613	MA_371398:6472-6473	0.0913369139207432	4.63999636335537	9.47340619765198e-05	0.0500007250277207

Figure S6: **The output of the Gmodel.**

It is a list of C-SNP pairs, where the SNP is the appropriate couple to explain CG in interest. The only different column from the Emodel output is the additional "snp" column next to the ID column. The output of the GxE model is the same.

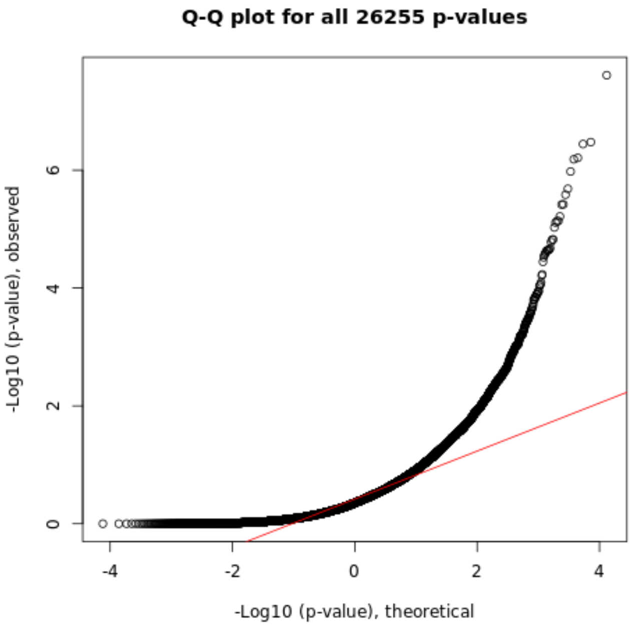

Figure S7: **QQ plots.**

Q-Q plots are generated with all models and give a theoretical vs observed distribution of all p-values. The x-axis is an indicator of normal distribution and ranges between [-4,4]. A total number of p-values can be seen in the header.

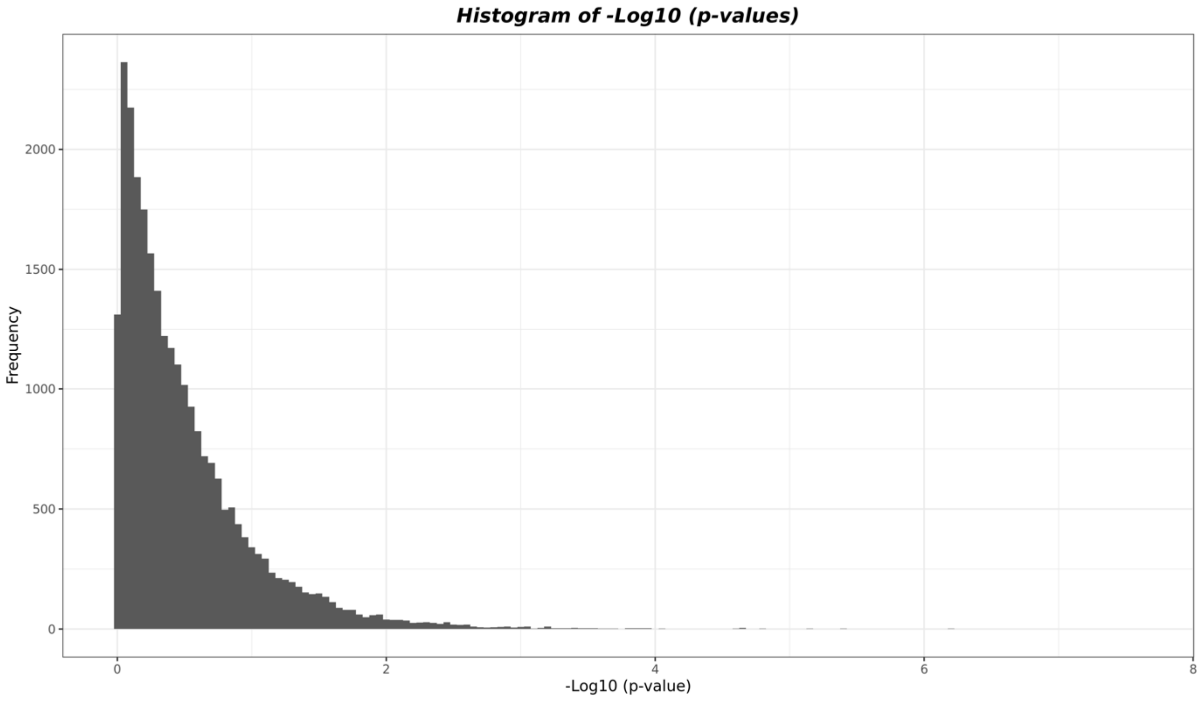

Figure S8: **Histograms plots.**

Histograms are generated with every model and drawn with all p-values to get an insight into their distribution. The x-axis is based on -$\log_{10}$ transformation and the y-axis show frequency.

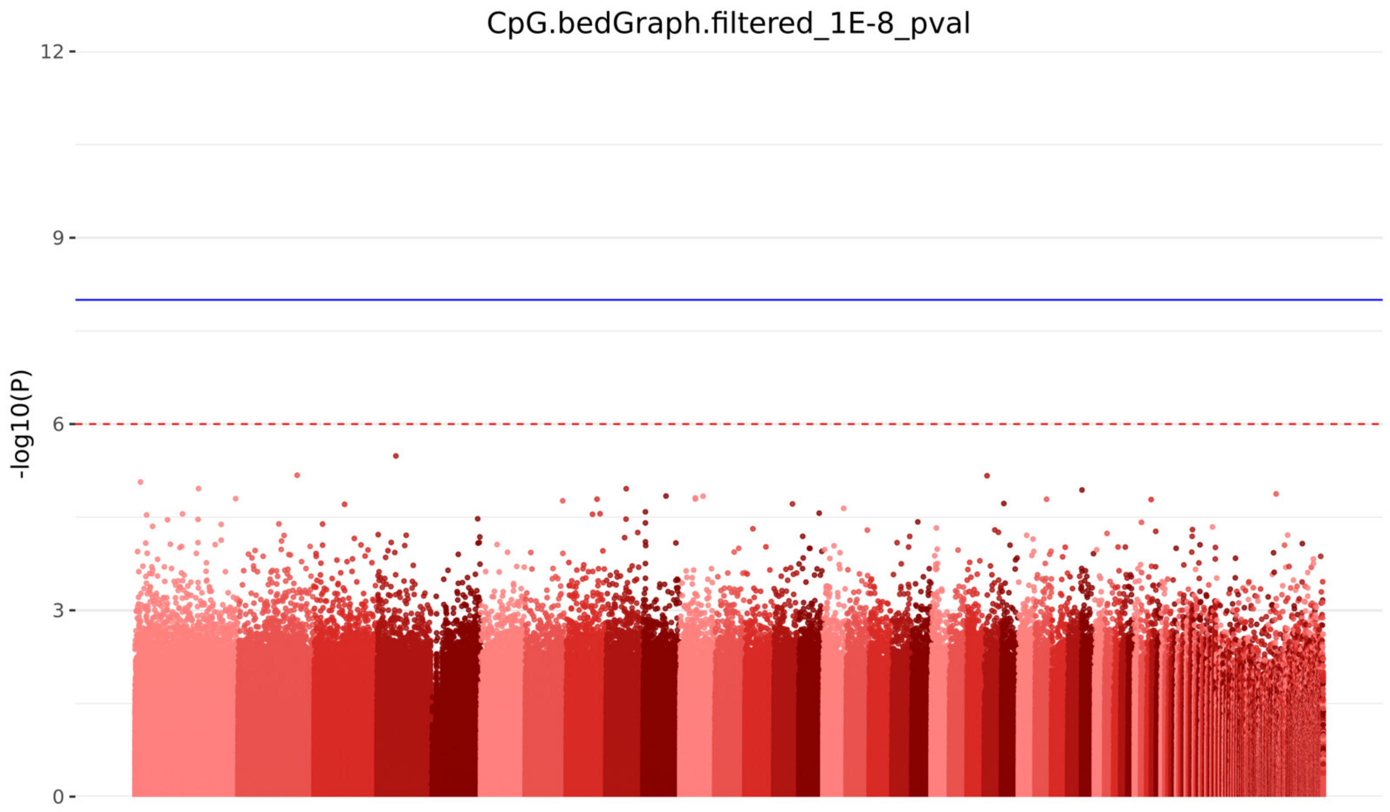

Figure S9: **Manhattan plots.**

Example of Manhattan plot output for CpG context and Emodel from the EWAS pipeline, representing all positions below p-value 1e-8. The dashed red line is a suggestive threshold (10-6 by default) and the blue line shows the epigenome-wide significance threshold (suggestive threshold / 100) to narrow down highly significant biomarkers above the lines.

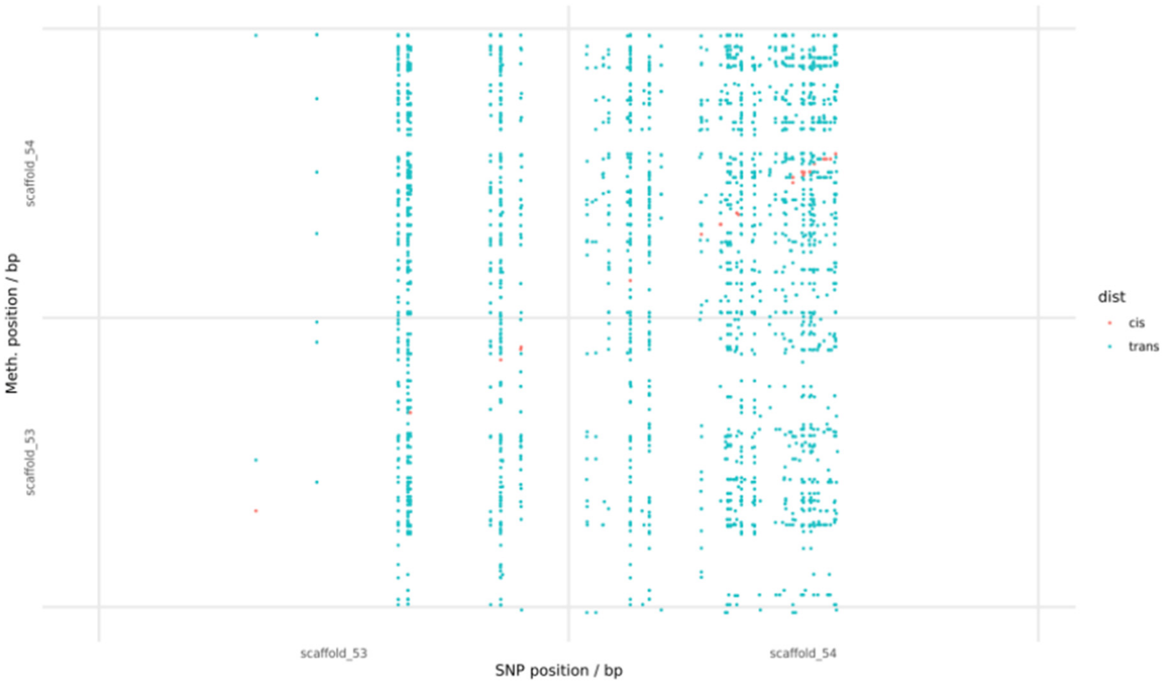

Figure S10: **Sequence dot plots.**

They are generated with the G model using Plotly with relative positions of SNPs and methylated positions in significant methQTLs. Cis and trans SNP-Cytosine pairs are marked as red and blue respectively. Scaffold/chr names are written on axes.

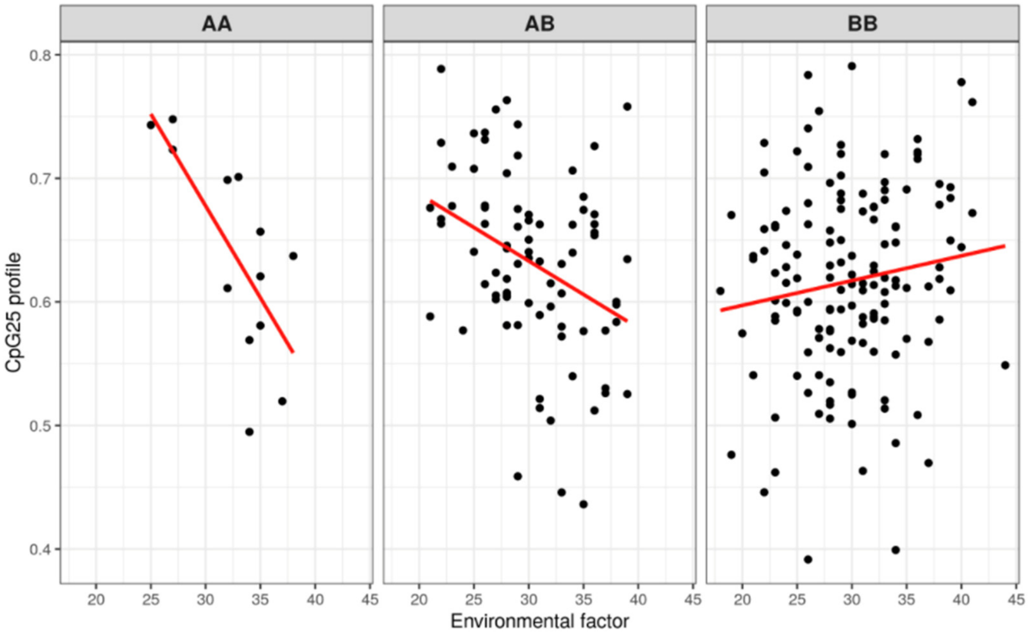

Figure S11: **Top significant k-plots.**

Top k-plots are generated with the phenotypic trait for major allele homozygote (AA), heterozygote (AB), and minor allele homozygote (BB) for all SNPs across all samples are generated with the GxE model. Significant Cytosine name is indicated at the left, not necessarily three alleles have to be produced with each C, the red line shows the slope of the linear relationship between individuals, dots (samples) and environmental factor (climatic data) is shown on the x-axis, methylation beta values are seen on the y-axis. Ref: Pan et al., 2016.

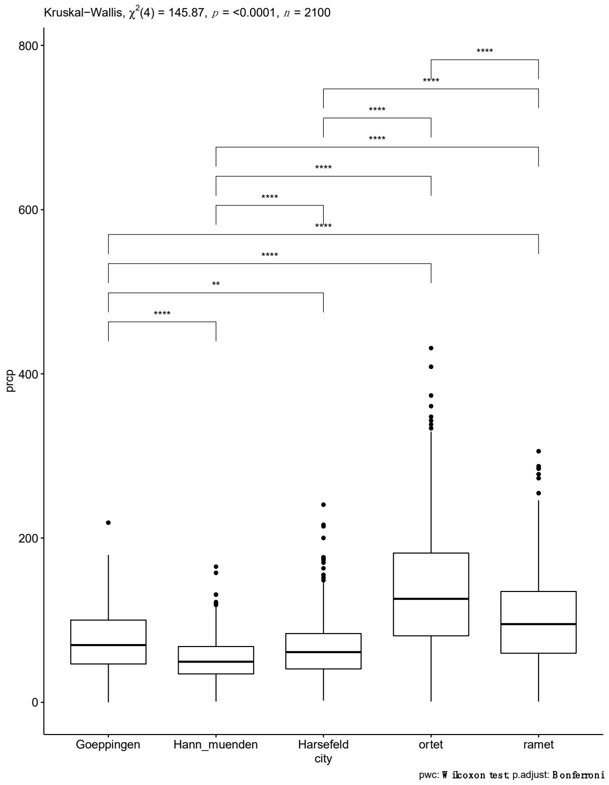

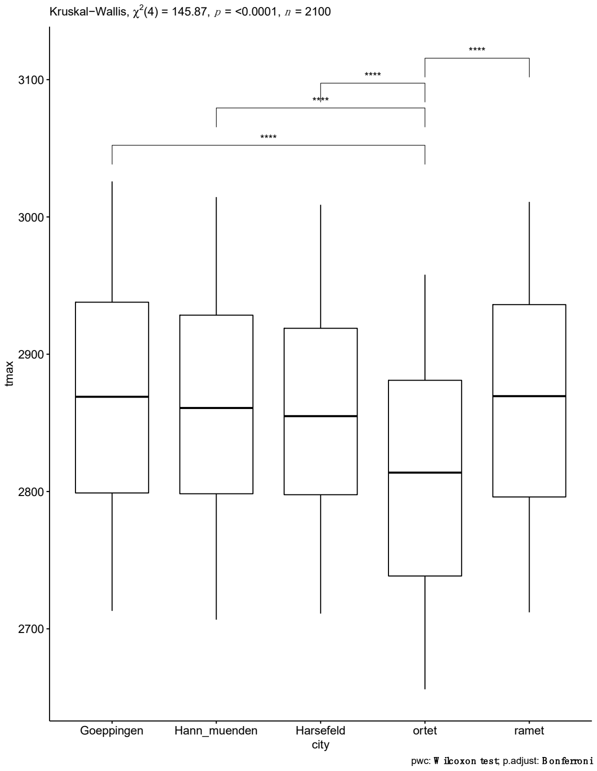

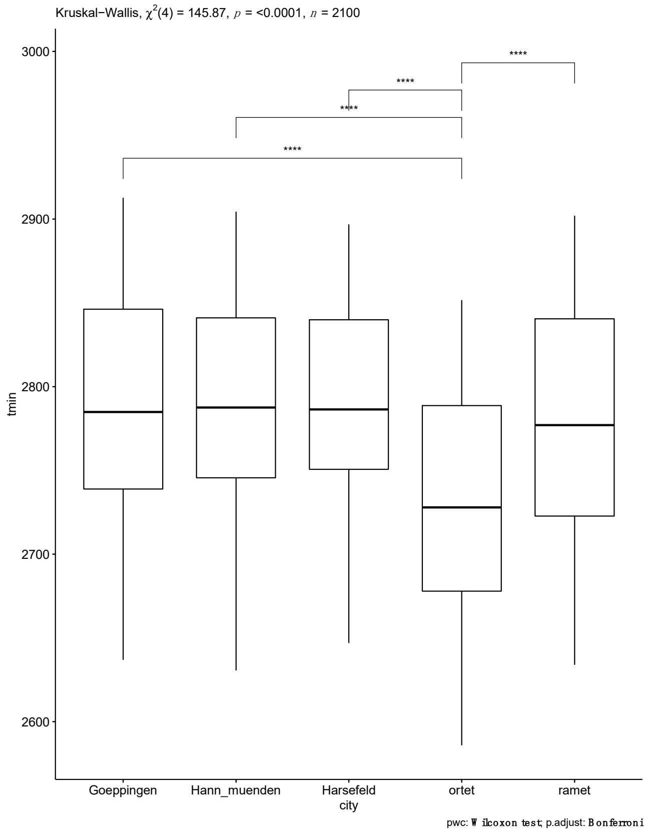

Figure S12: **Non- parametric Wilcoxon test to compare climatic datasets for locations of trees.**

Boxplots and test results for precipitation (prcp) (upper left), maximum temperature (tmax) (upper right), and minimum temperature (lower) datasets. All differences were found significant for the prcp data. Differences between ortet and all other locations were found significant for tmax and tmin data.

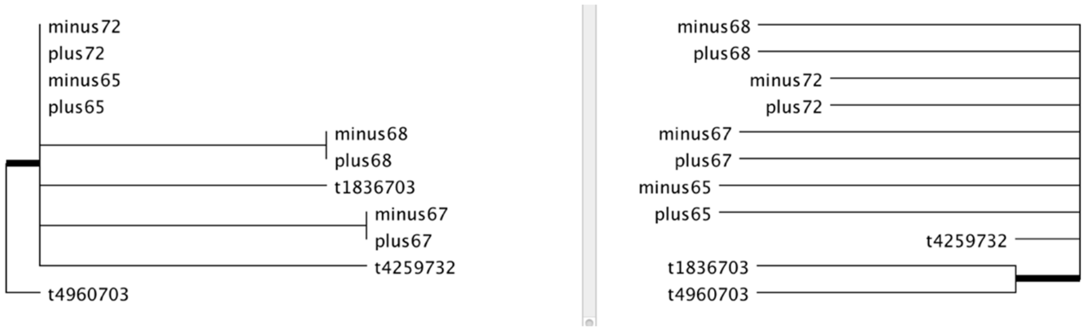

Figure S13: **Coalescence analysis with SNP and averaged methylation data for the CG context.**

Averaged methylation calls (left) and SNP (right) tree comparison output with compare2trees software [3] for the CG context. This comparison yields a 72% topological score indicating a relatively high fraction of clades/branches present in both trees (cf. Methods for details). The thick branches represent deviating topologies.

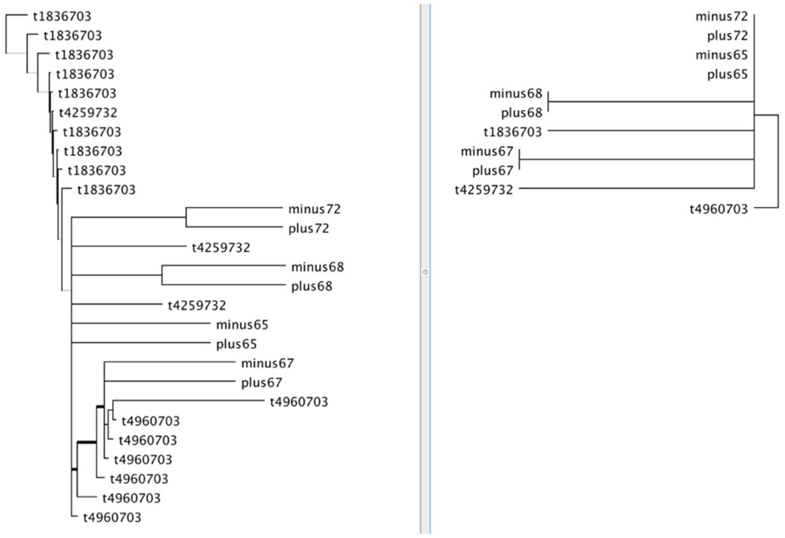

Figure S14: **Coalescence analysis with SNP and not averaged methylation data for the CG context.**

Not averaged methylation calls (left) and SNP (right) tree comparison output with compare2trees software [3] for the CG context. This comparison has a 78.7% over topological score indicating a relatively high fraction of clades/branches present in both trees (cf. Methods for details). The thick branches represent deviating topologies.

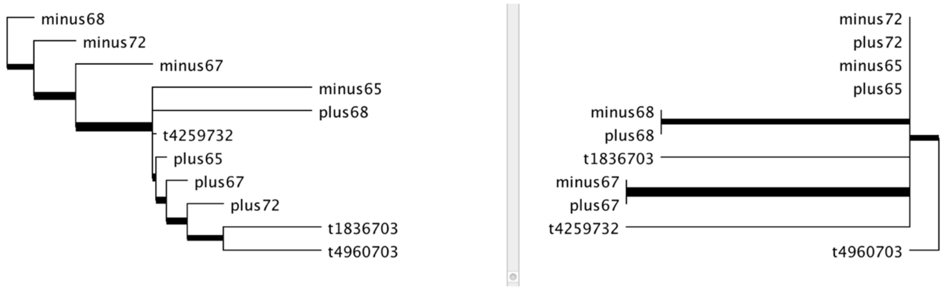

Figure S15: **Coalescence analysis with SNP and averaged methylation data for the CHG context.**

Averaged methylation calls (left) and SNP (right) tree comparison output with compare2trees software [3] for the CG context. This comparison has a 38.2% over topological score indicating a relatively low fraction of clades/branches present in both trees (cf. Methods for details). The thick branches represent deviating topologies.

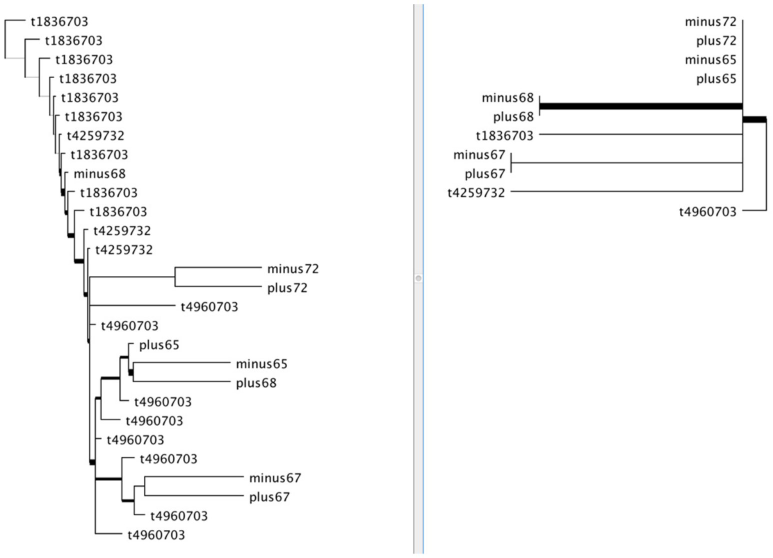

Figure S16: **Coalescence analysis with SNP and not averaged methylation data for the CHG context.**

Not averaged methylation calls (left) and SNP (right) tree comparison output with compare2trees software [3] for the CG context. This comparison has a 58.5% over topological score which indicating a medium fraction of clades/branches present in both trees (cf. Methods for details). The thick branches represent deviating topologies.

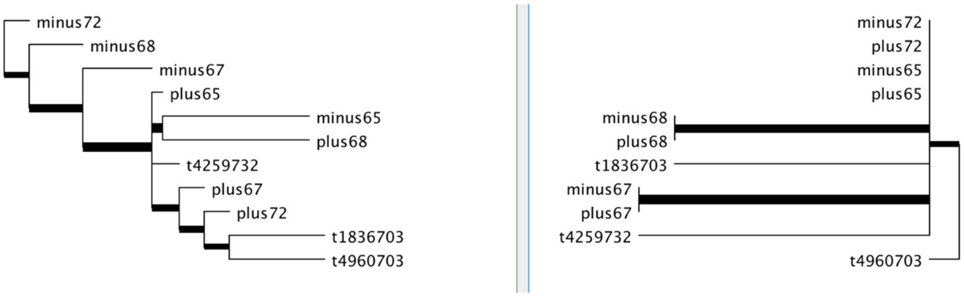

Figure S17: **Coalescence analysis with SNP and averaged methylation data for the CHH context.**

Averaged methylation calls (left) and SNP (right) tree comparison output with compare2trees software [3] for the CG context. This comparison has a 42.3% over topological score indicating a relatively low fraction of clades/branches present in both trees (cf. Methods for details). The thick branches represent deviating topologies.

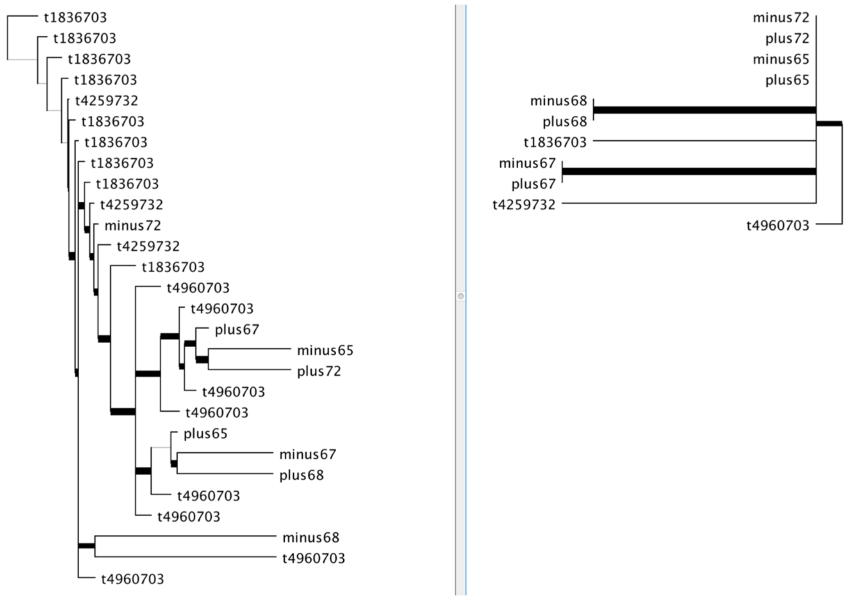

Figure S18: **Coalescence analysis with SNP and not averaged methylation data for the CHH context.**

Not averaged methylation calls (left) and SNP (right) tree comparison output with compare2trees software [3] for the CG context. This comparison has a 35.3% over topological score indicating a relatively low fraction of clades/branches present in both trees (cf. Methods for details). The thick branches represent deviating topologies.

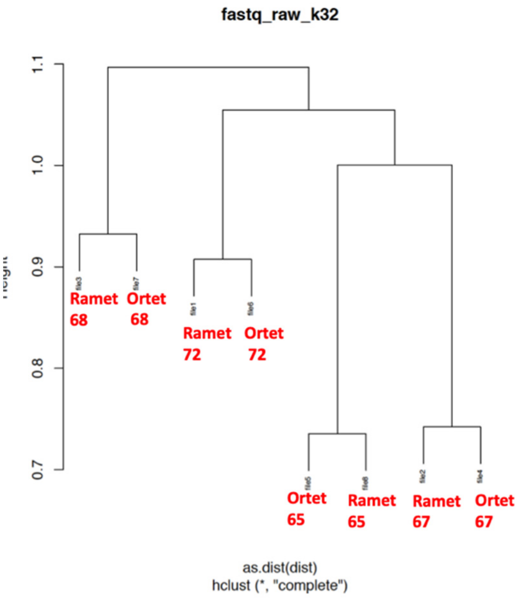

Figure S19: f**astq raw files HC with k32 done by kWIP software [4].**

Clear clustering is seen between clones and original tree pairs can be pointed and names in red were added later to clarify observation.

DMP/DMR Analysis using different callers

Metilene was not able to call FDR corrected DMPs due to the implemented statistics (the rank test does not have enough power to yield significant q-values). The high total number of tests required to process all DMPs leads to no significant results after FDR filtering which is a relatively common problem of adjusting p-values for big datasets [5]. Yet, as shown above, almost all q-value filtered defiant DMPs and the majority of q-value filtered methylkit DMPs are shared with the p-value filtered metilene DMPs. This shows that DMP calling is relatively robust, but that using metilene's p-value filtered DMPs will include many (potentially false positive) positions not detected when applying FDR correction. To test whether this abundance of positions would bias the detection of significant positions, we compared the EWAS output for GC context of the ramet vs. ortet comparison (based on the metilene $p < 0.05$ DMPs) with the defiant results. We found that the overlap is significantly (Fisher's exact test, $p < 0.05$) more than expected by chance for G as well as the GxE model for the prcp environmental variable (Figure S20c).

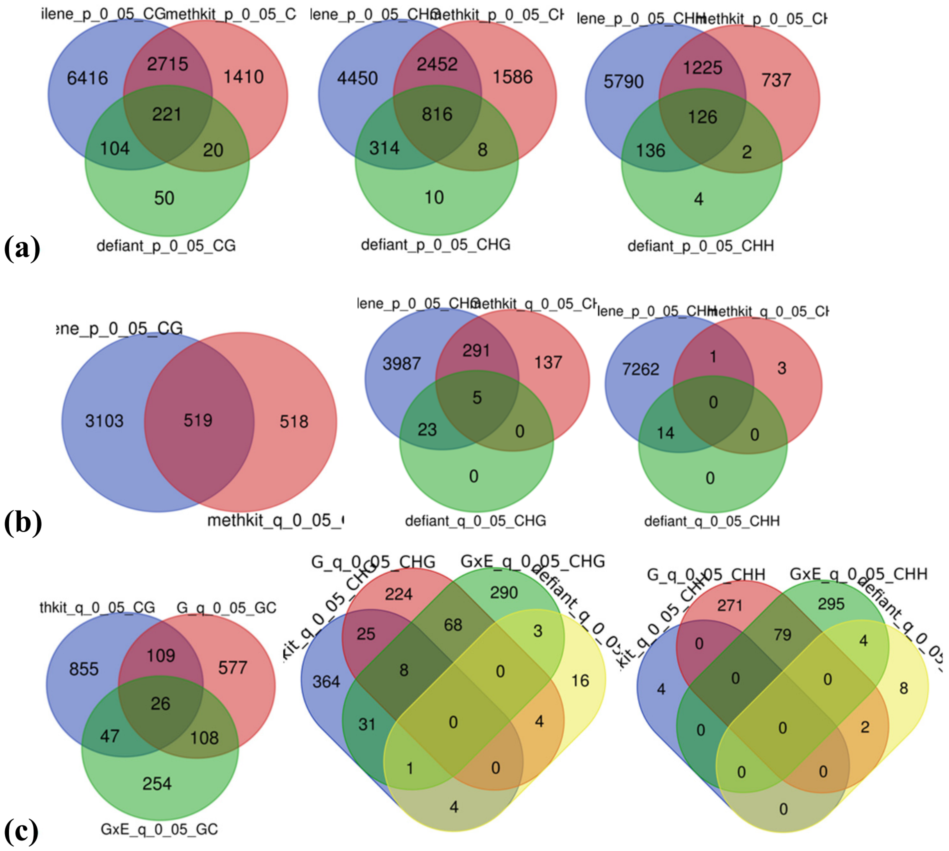

Figure S20: **Intersection of significant Cs with p and q-values on gene-level for methylkit, metilene, and defiant DMR callers.**

(a) Genes with 0.05 p-value cut-off between metilene, methylkit, and defiant for CG (left), CHG (middle), and CHH (right) for ramets vs ortets comparison. 32%, 44% & 20% of metilene genes, 0.55, 0.90 & 0.98 of defiant DMPs and 67%, 67% & 64% of methylkit genes are shared with other two. Metilene genes have the highest excluded amount. Diagrams (separated by contexts like CG, CHG, and CHH from left to right) in (b) shows the 0.05 q-value cut-off genes with DMR callers again with ramets vs ortets comparison, unfortunately, no gene(s) were found related to the CG context DMP(s) and 14% of metilene genes are shared with the methylkit ones. All, 7% and 68% of DMPs are shared by either one of two other genes for defiant, metilene, and methylkit respectively for CHG context and numbers are like again all, 0.1% and 25 for defiant, metilene, and methylkit. Diagrams in (c) show the intersection between pipeline output and the DMR callers between ramets and ortets. 13% and 7% of methylkit genes are shared between Gmodel and GxE models (left). CHG (middle) and CHH (right) genes exclusively shared by a single diagram of two diagrams.

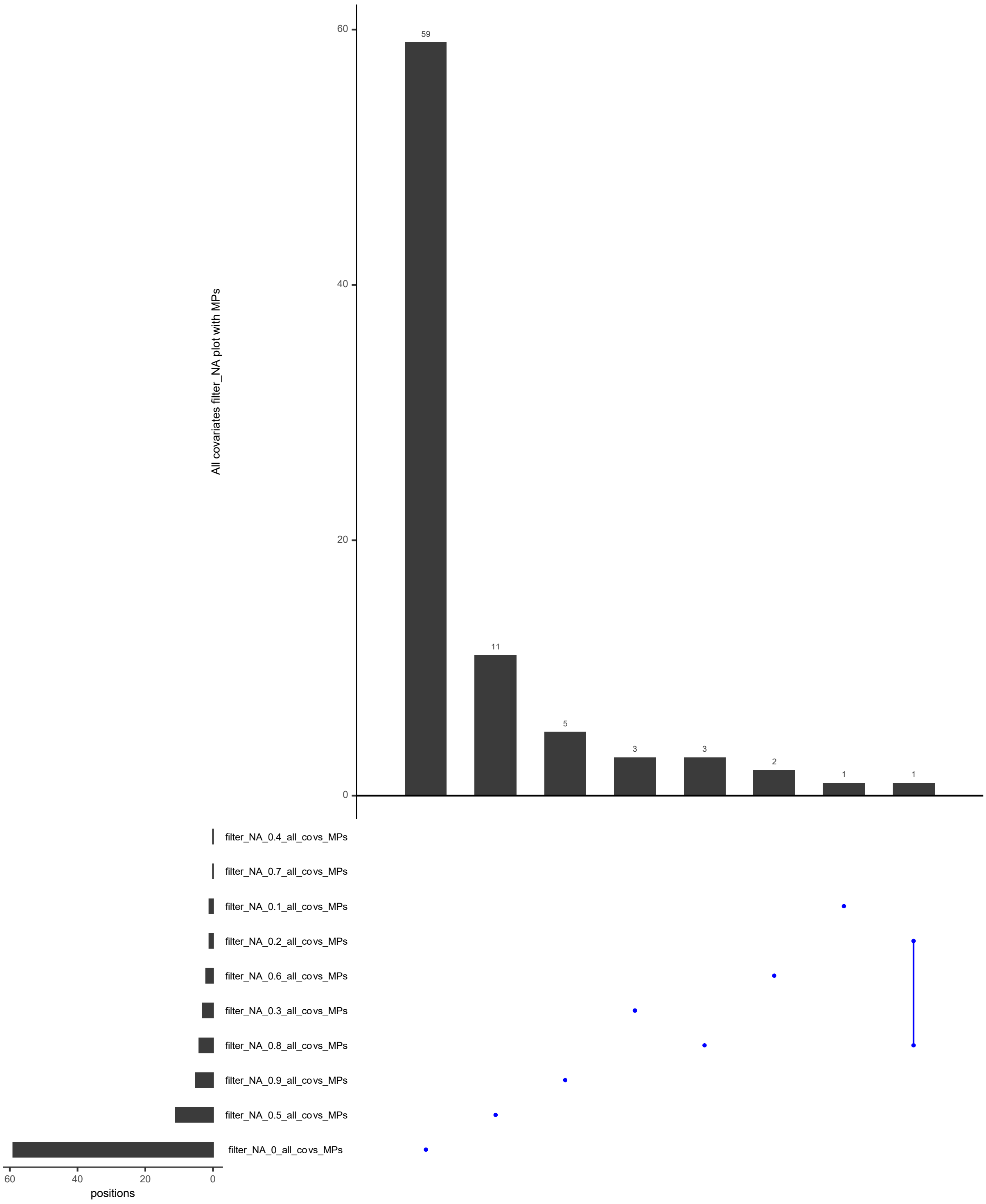

Figure S21: **Intersection of outputs with different filter_NA values for MPs input using all covariates.**

There is a single intersection with 0.2 and 0.8 filter_NA values and the rest has unique outputs.

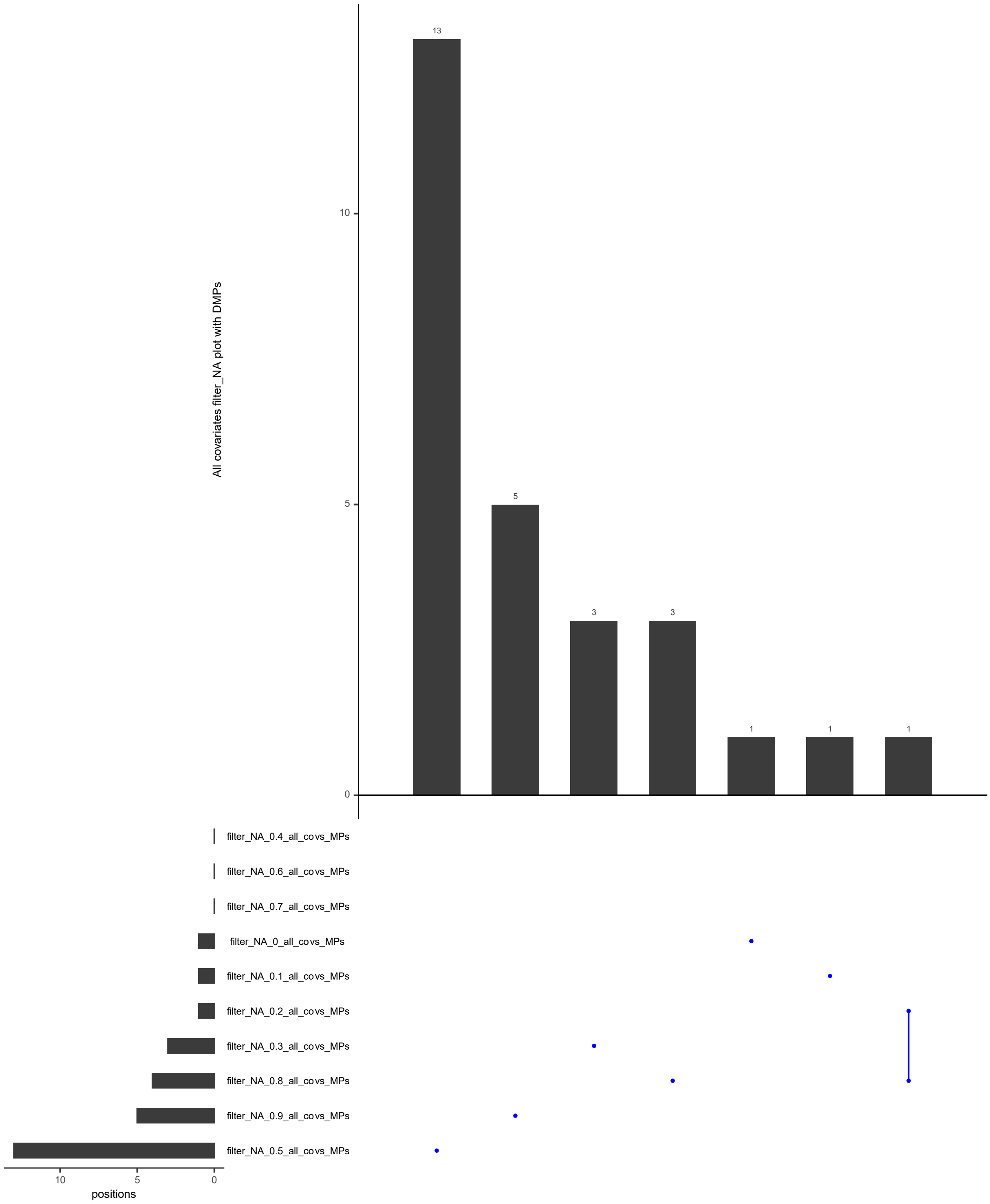

Figure S22: **Intersection of outputs with different filter_NA values for DMPs input using all covariates.**

There is a single intersection with 0.2 and 0.8 filter_NA values and the rest has unique outputs.

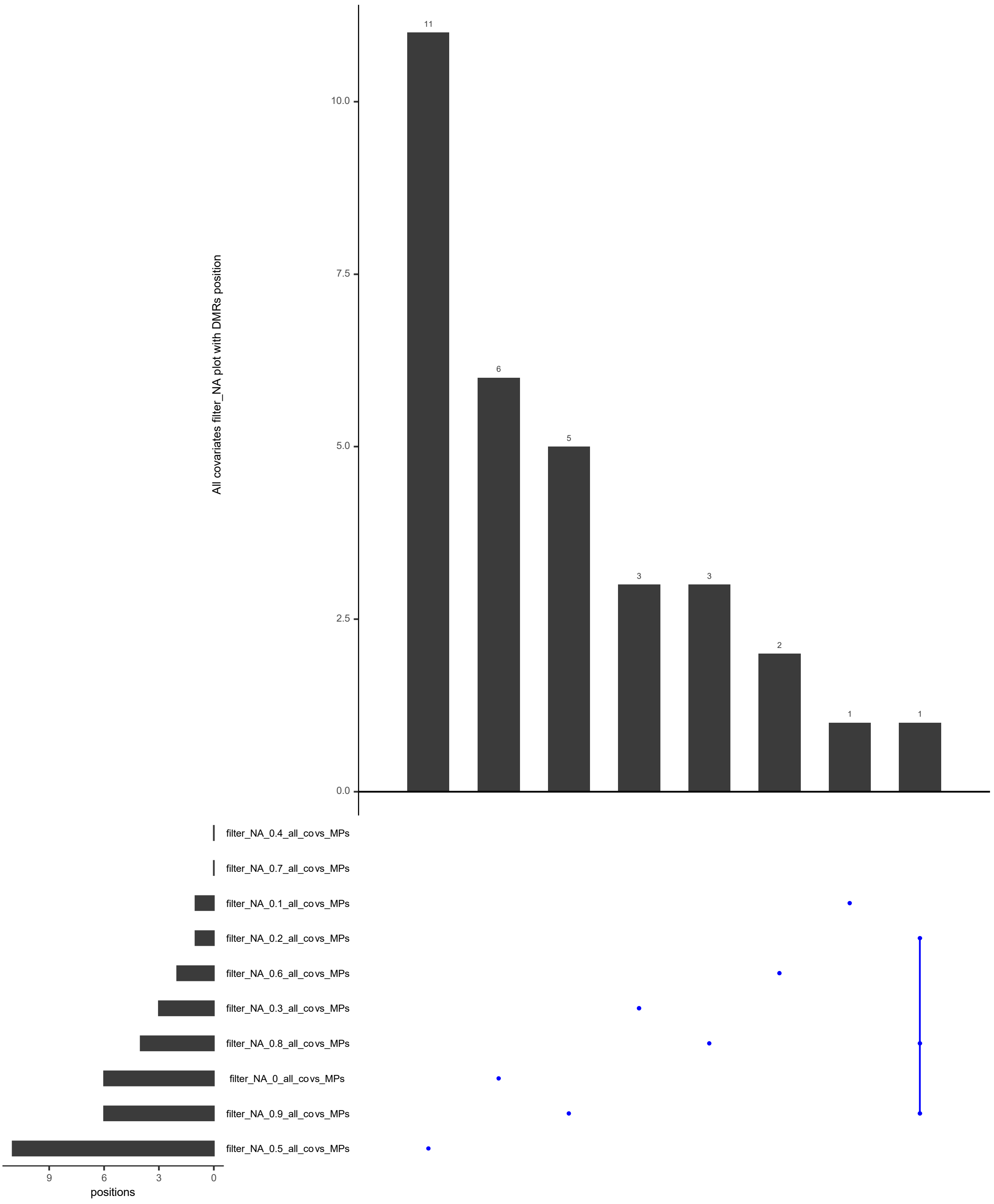

Figure S23: **Intersection of outputs with different filter_NA values for DMRs input using all covariates.**

There is a single intersection with 0.2, 0.8, and 0.9 filter_NA values, and the rest has unique outputs.

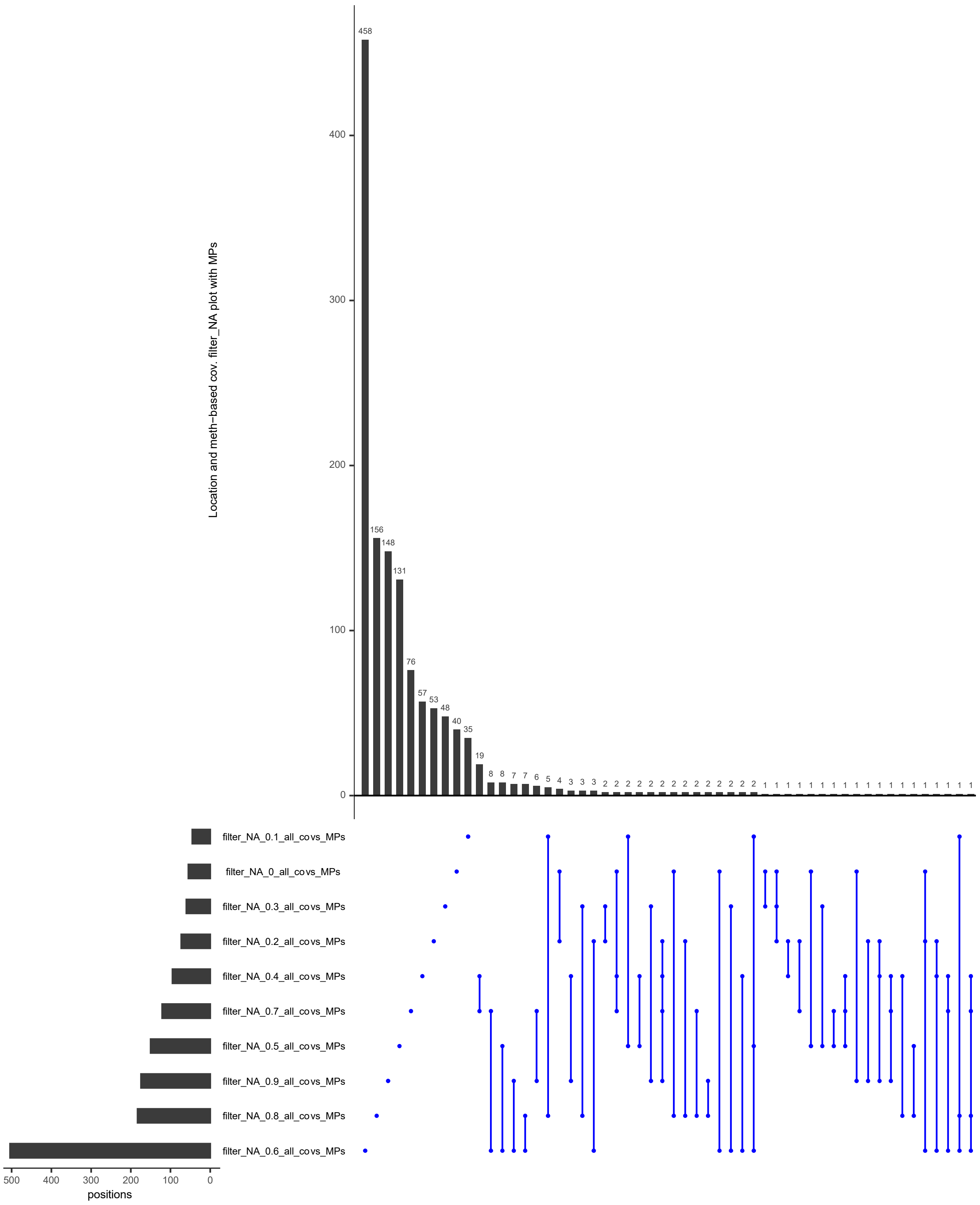

Figure S24: **Intersection of outputs with different filter_NA values for MPs input using only location-methylation-based covariates.**

The 0.4, 0.6, 0.8, and 0.8 filter_NA values have a shared gene, besides this, there are a lot of shared genes between outputs.

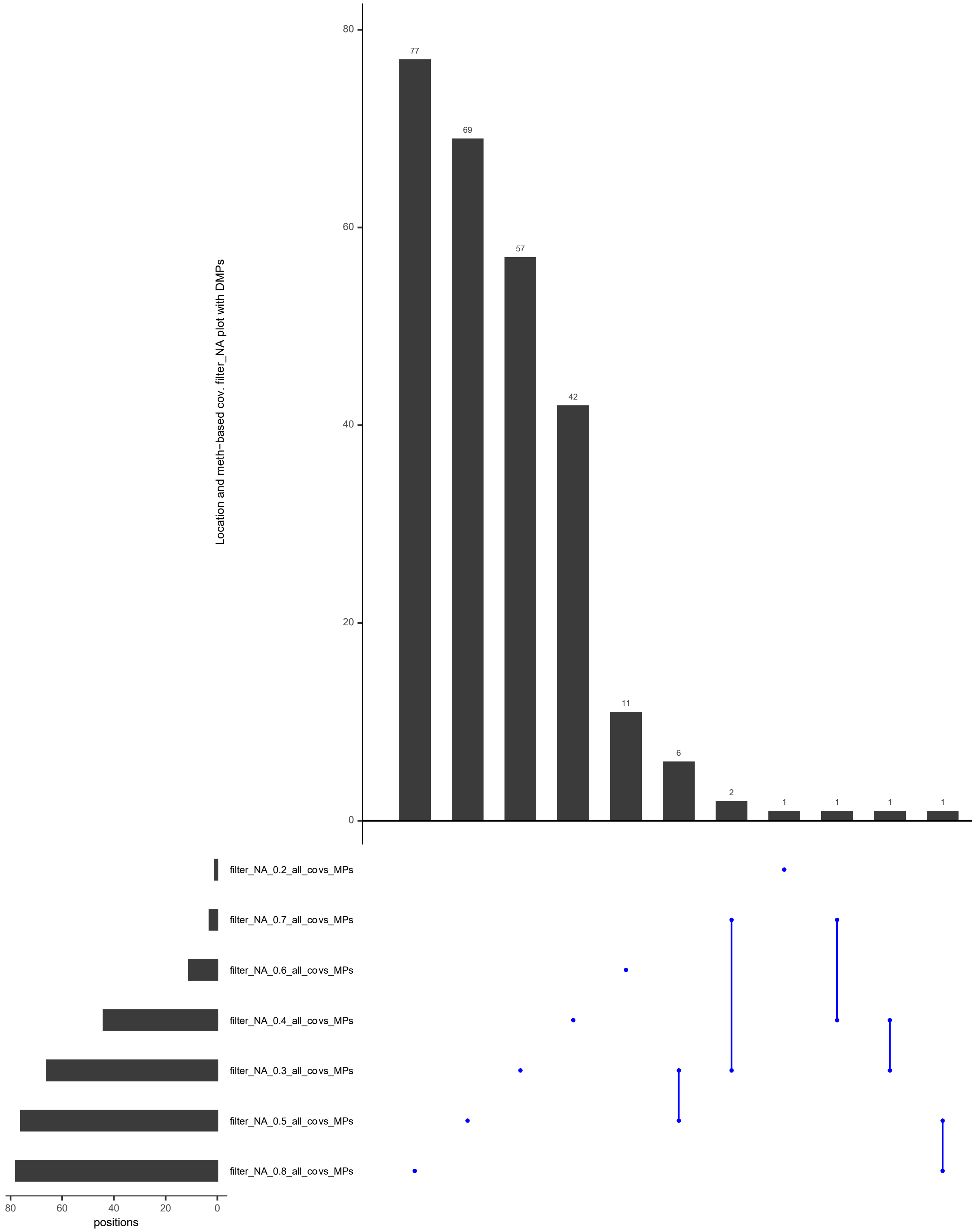

Figure S25: **Intersection of outputs with different filter_NA values for DMPs input using only location-methylation-based co-variates.**

0.3 and 0.5 NA filtering outputs have the maximum number of common genes as six and 0.7 and 0.3 intersections follow it with two genes for DMPs input (upper right). A single gene is common between 0.7 & 0.4, 0.4 & 0.3 and 0.5 & 0.8.

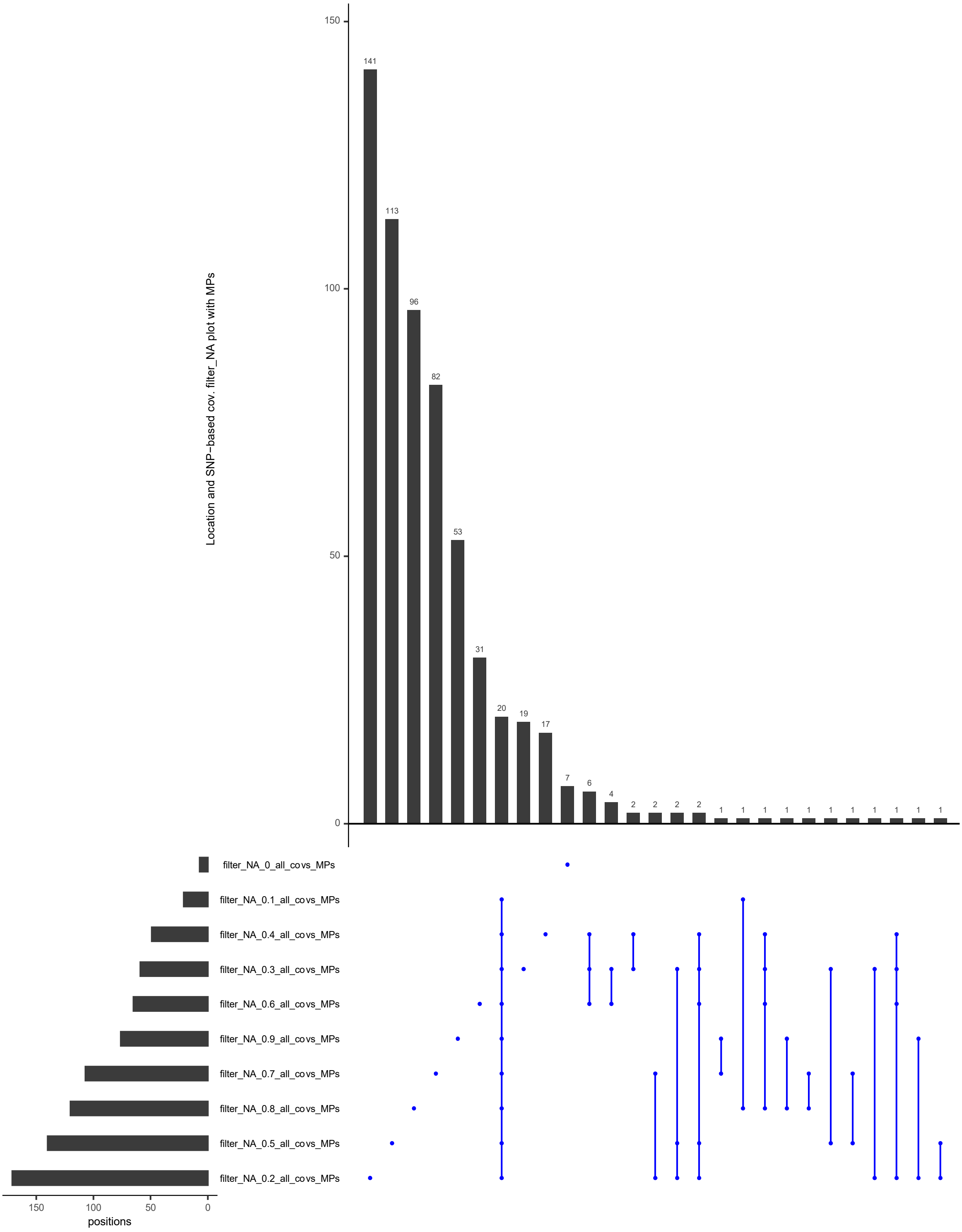

Figure S26: **Intersection of outputs with different filter_NA values for MPs input using only location-SNP-based covariates.**

There are a lot of shared genes between outputs. 20 elements are shared between all groups but 0 filter_NA.

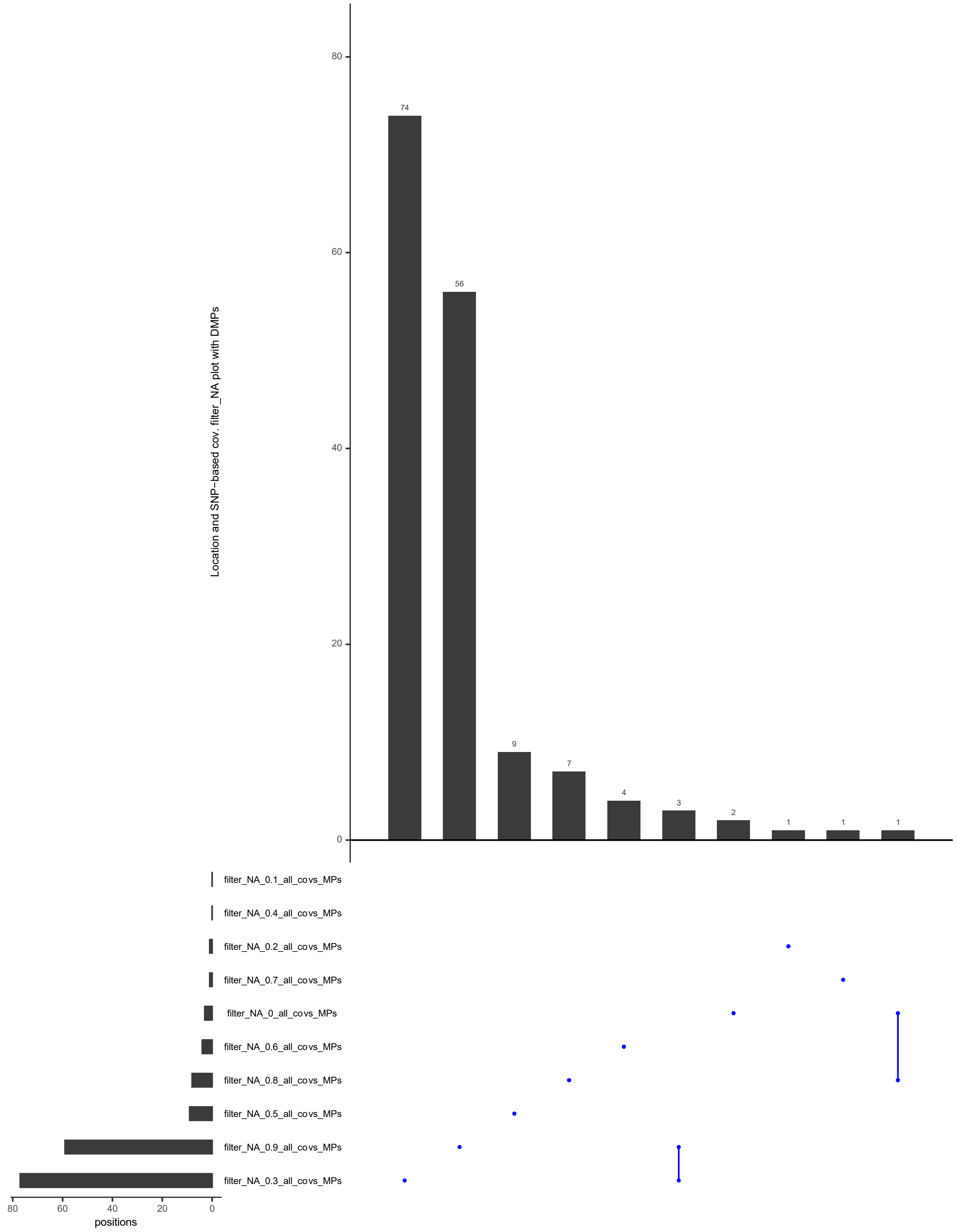

Figure 27: **Intersection of outputs with different filter_NA values for DMPs input using only location-SNP-based covariates.**

0 and 0.8 NA & 0.9 and 0.3 filtering outputs share a one and three gene(s) in common.

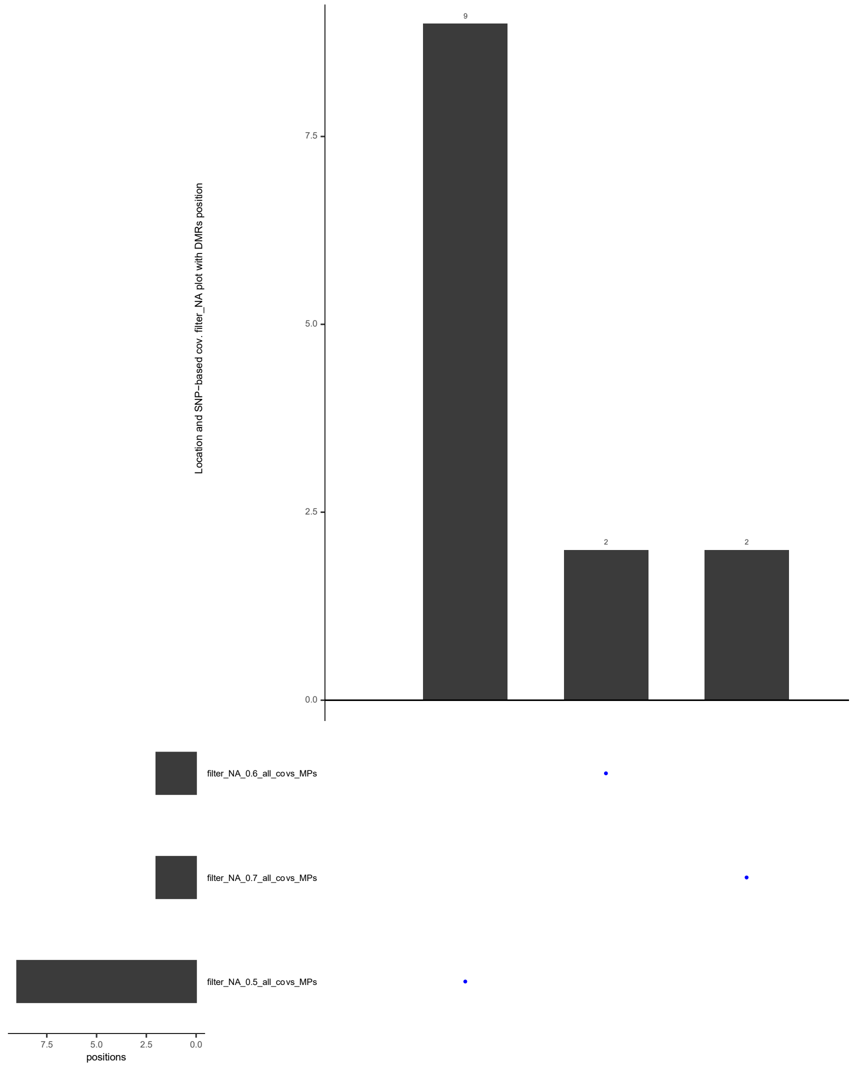

Figure S28: **Intersection of outputs with different filter_NA values for DMRs input using only location-SNP-based covariates.**

Nothing is shared between outputs.

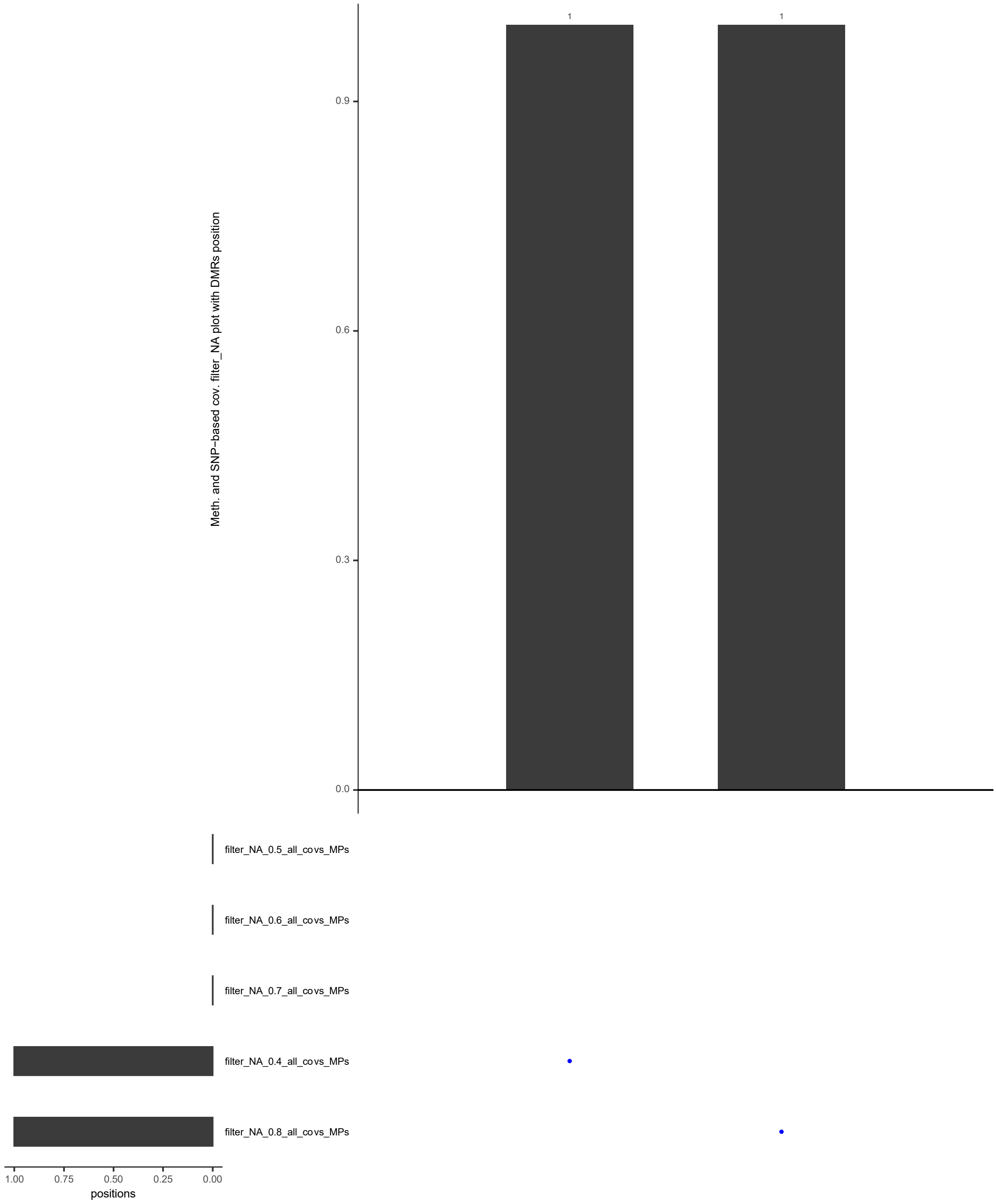

Figure S29: **Intersection of outputs with different filter_NA values for MPs input using only SNP-methylation-based covariates.**

Nothing is shared between outputs.

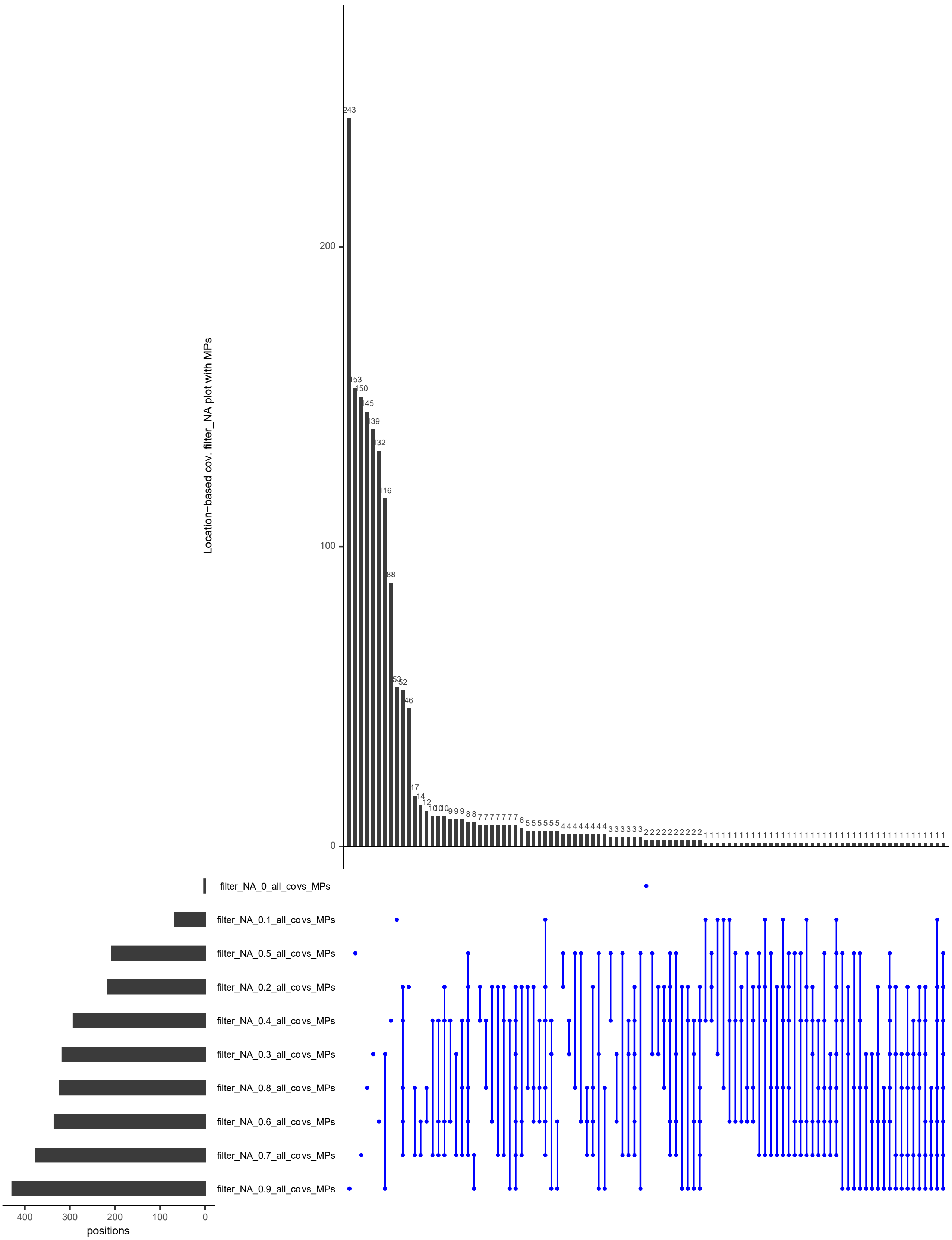

Figure 30: **Intersection of outputs with different filter_NA values for MPs input using only location-based covariates.**

This input has the maximum number of shared elements.

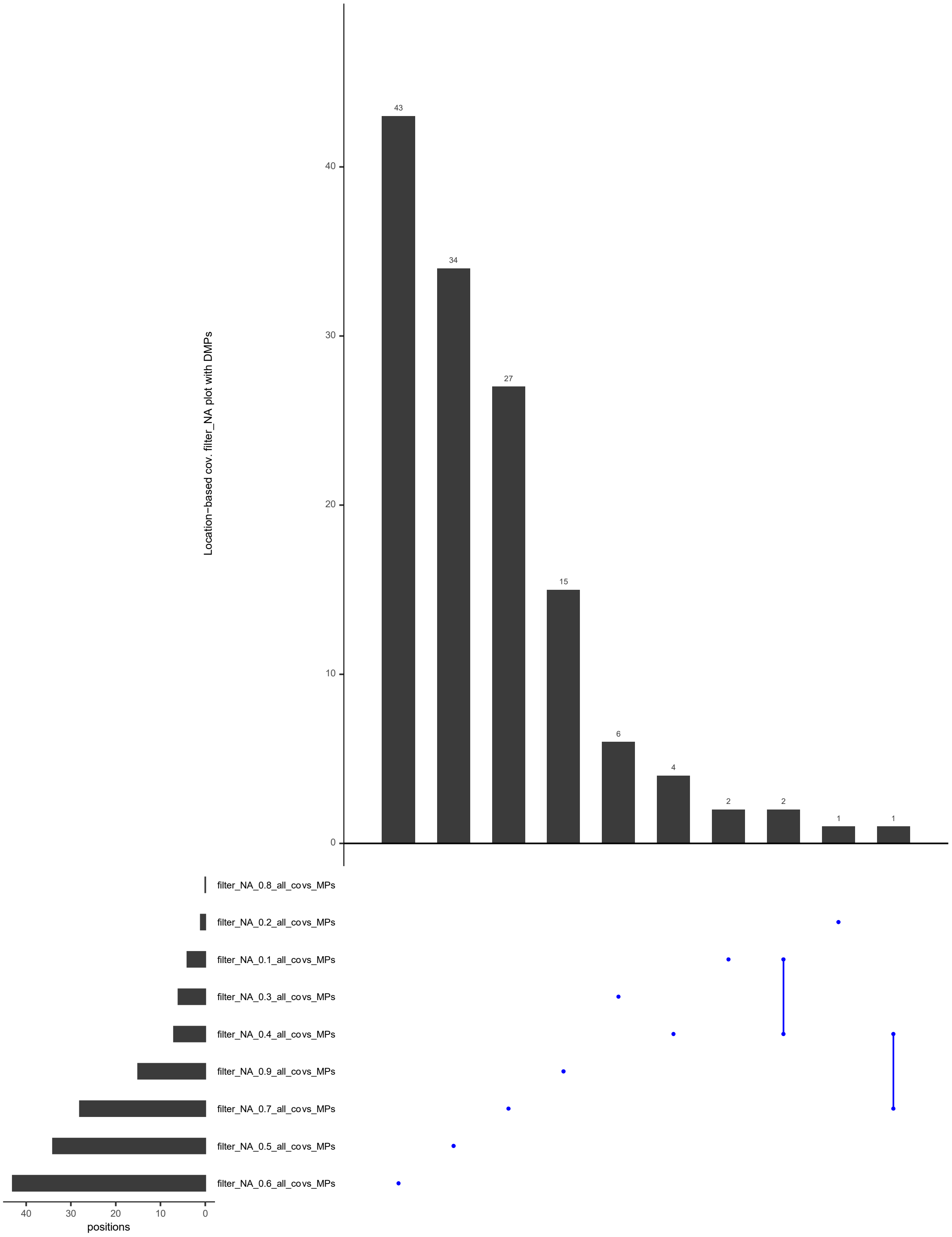

Figure 31: **Intersection of outputs with different filter_NA values for DMPs input using only location-based covariates.**

This input showed a single gene in common between 0.1 & 0.4 and 0.4 & 0.7 filters.

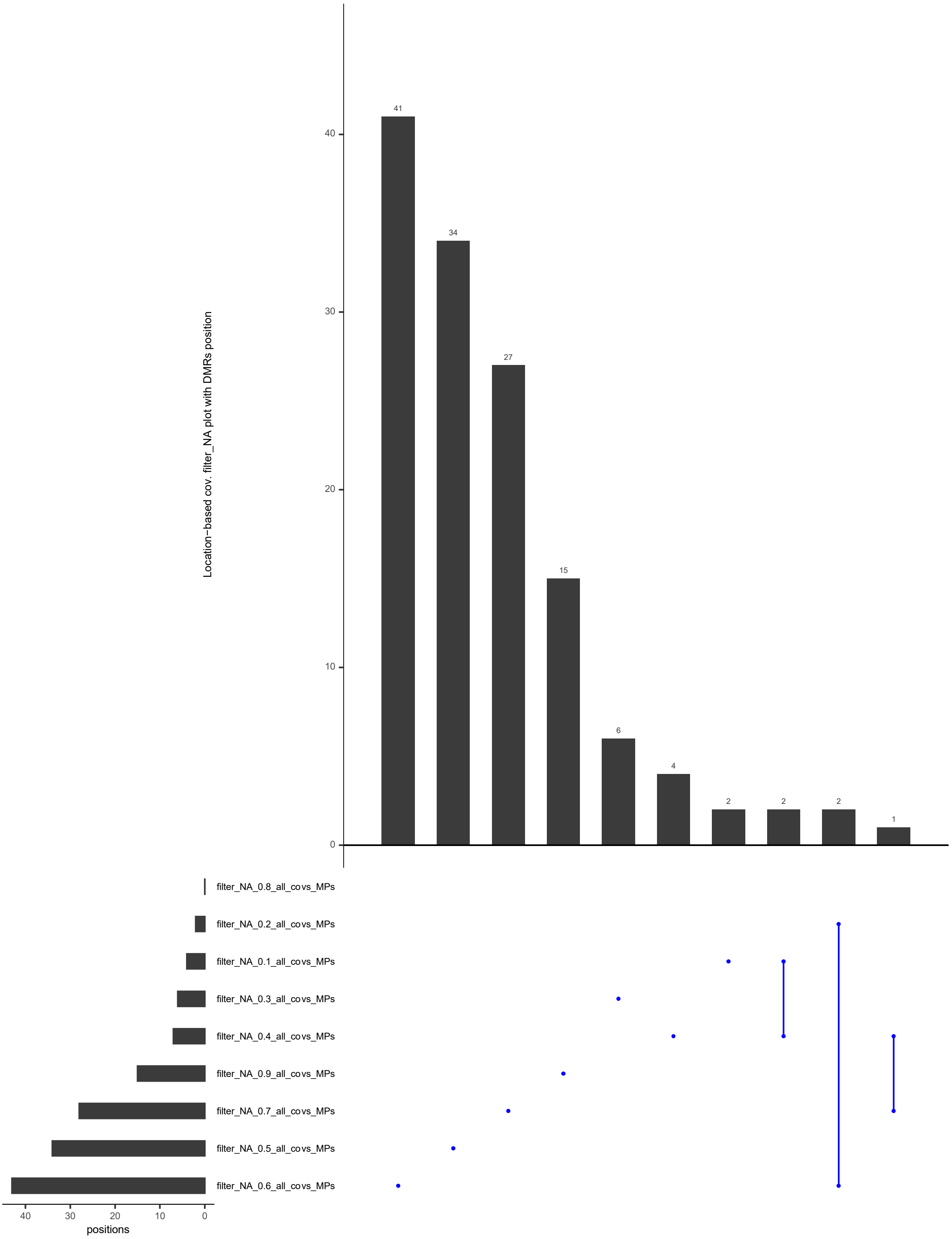

Figure S32: **Intersection of outputs with different filter_NA values for DMRs input using only location-based covariates.**

This input showed an intersection between 0.1 & 0.4 and 0.2 & 0.5 filter_NA's with two genes, 0.4 & 0.7 filter_NA with a single gene in common.

Removal of genetic variants that might be interpreted as significant epigenetic marks

Consider a SNP that will change a CG to a GG –methylation of the position will not be possible anymore and it might appear as a significant position in EWAS. If it's a homozygous SNP, then there will be a C in the reference genome and all Gs in the alignments (or Cs on Crick strand) so it will just be interpreted as a normal SNP by a haplotype-based variant detector. If it's a heterozygous SNP, then on the crick strand everything is read as normal FreeBayes [6], but the Cs on the Watson strand will likely be converted to Ts by the bisulfite treatment and these will be ignored. The Gs on Watson will be interpreted normally so as the result it will look like a heterozygous CG to GG SNP.

Such sites that might lead to a loss or gain of a methylation site based on genetic divergence can be investigated by intersecting the Emodel results with either Gmodel and/or GxE. If there are SNPs that will lead to a context change, intersecting the model outputs solves this issue. In the case of a homozygous SNP, there will be a C in the reference and Gs in the alignments (or Cs on the Crick strand), so the position will be interpreted as a normal SNP by a variant calling software, FreeBayes. In the case of a heterozygous SNP, the crick strand is read as normal by FreeBayes, but the Cs on the Watson strand will likely be converted to Ts by the bisulfite treatment, and these will be ignored. The Gs on Watson will be interpreted normally. This case will be interpreted as a heterozygous C > G SNP by the bisulfite aware variant callers such as the EpiDiverse SNP pipeline and solved. We were looking into this in detail in an example by checking CG sites shared by all models.

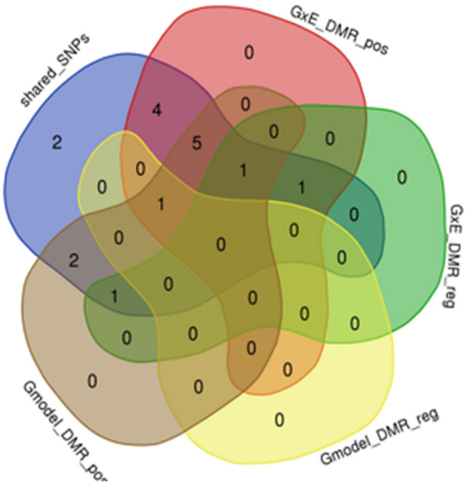

Figure S33: **Intersection of shared SNPs and significant common markers between G and GxE models.**

Only a single position is shared between Emodel MP & DMP inputs and G & GxE models DMPs input. The number of SNPs correlated with that shared positions is 1, 16, for G and GxE models respectively.

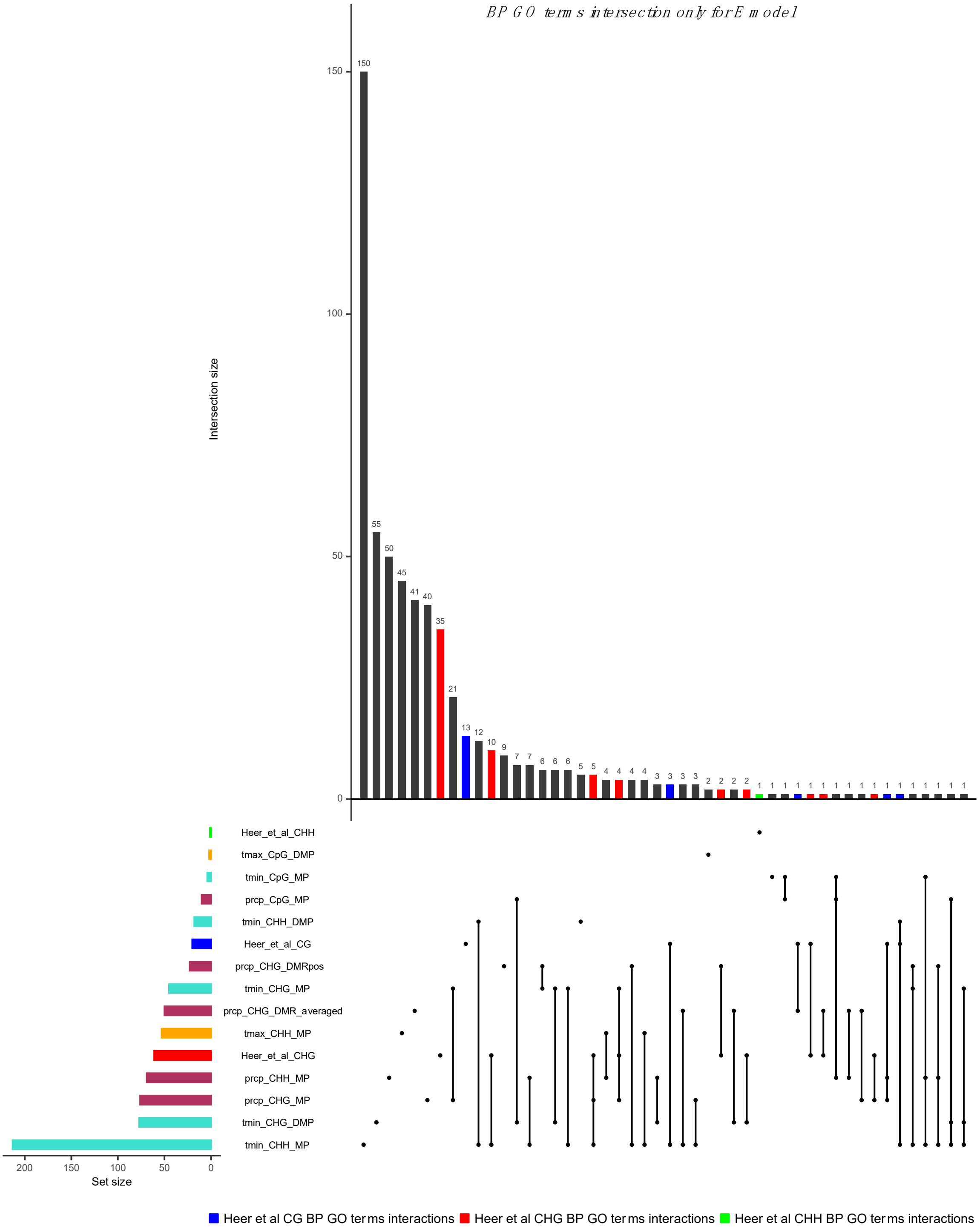

Figure S34: **Intersection of significant BP GO terms between location-based Emodel output and a previous study [7] with UpsetR package [8].**

Common BP GO terms are connected via vertical lines. No output could be derived with SNP and methylation-based clustering, therefore only results of location-based clustering are shown. Bars are ordered from smallest to largest by sample size. The maximum and the minimum number of unique elements belong to the tmin_CHH_MP and tmax_CG_DMP inputs, with 150 and one elements respectively. 47%, 45%, and 0% of BP GO terms are shared with Heer et al., 2016 study for CG the (blue), CHG (red), and CHH (green) contexts. Tmin, prcp, and tmax sets are colored as turquoise, maroon, and orange.

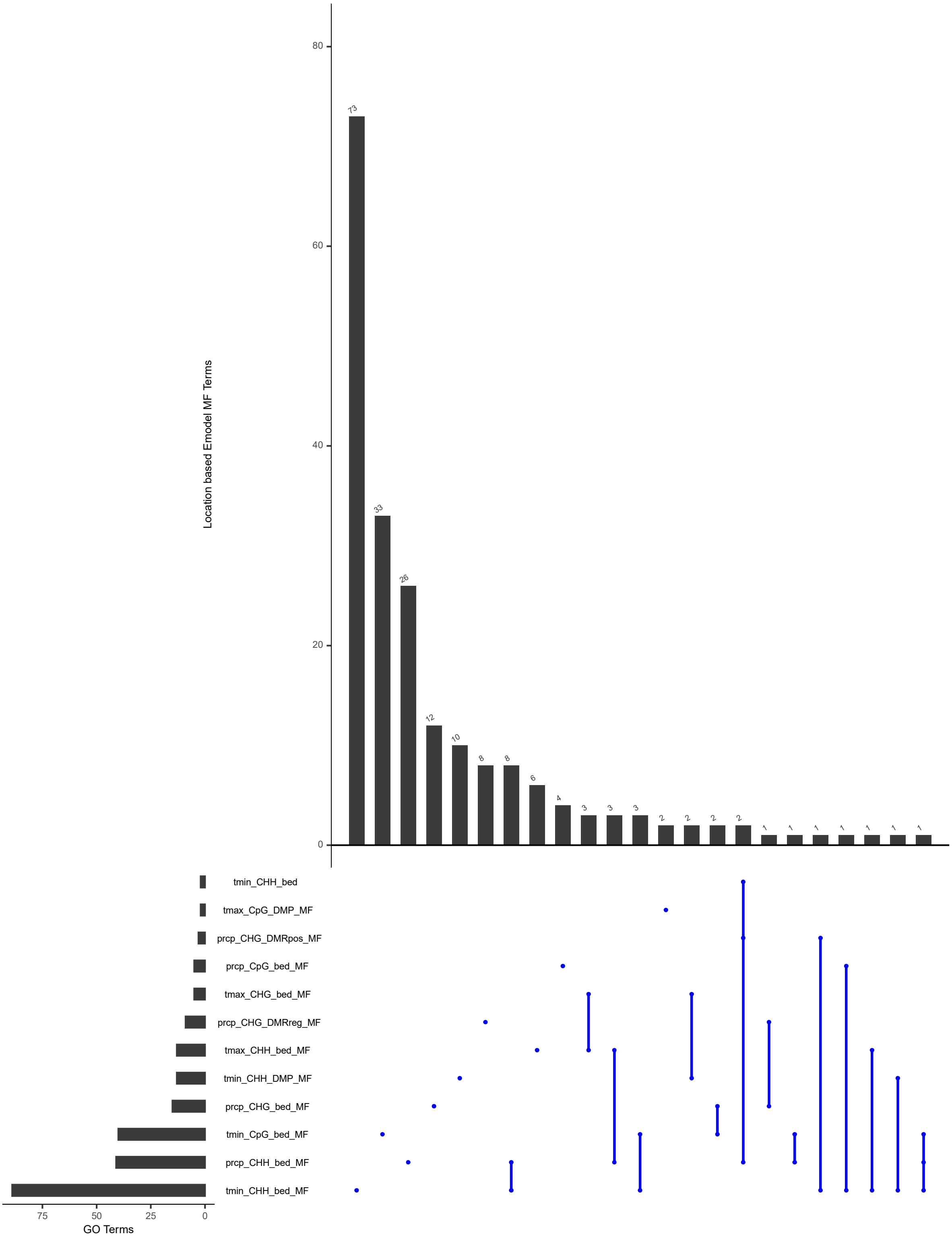

Figure S35: **Intersection of significant MF GO terms between location-based Emodel output and a previous study [7] with UpsetR package [8].**

Three terms are shared by three groups, 27 terms are shared by two groups and 174 terms are uniquely shared by single inputs. The highest number of elements belongs to CHH tmin MP input with 73 terms and the lowest is with two terms for the CG tmax DMP input. 15% of terms are shared by inputs and 85% is unique to single inputs.

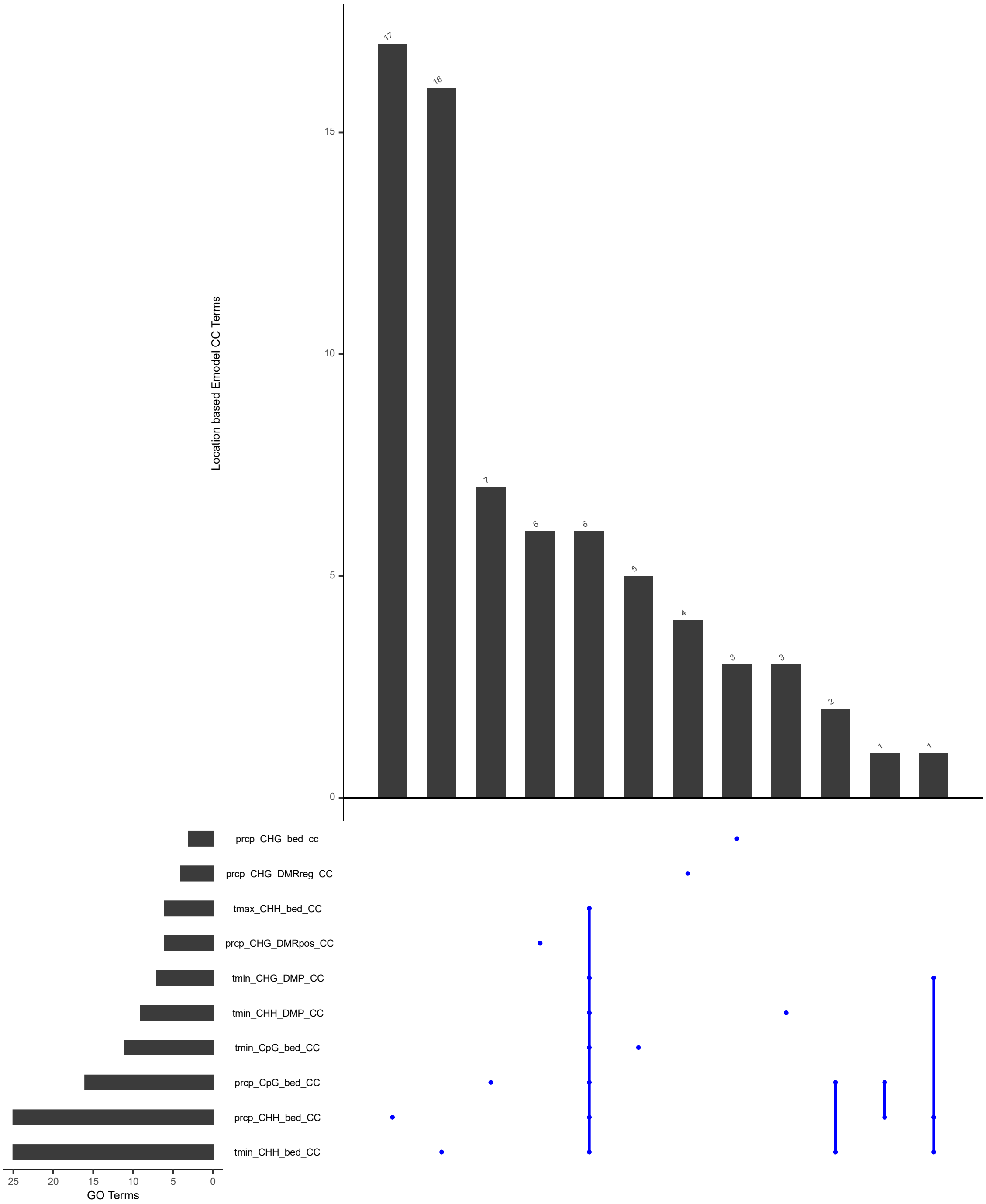

Figure S36: **Intersection of significant CC GO terms between location-based Emodel output and a previous study [7] with UpsetR package [8].**

Six terms are shared by seven groups, one term is shared by three groups, three terms are shared by two groups and 61 terms are uniquely shared by single inputs. The highest number of elements belongs to CHH prcp MP input with 17 terms and the lowest is with three terms for the CHH tmin DMP input. 15% of terms are shared by inputs and 85% is unique to single inputs.

	Emodel											
	prcp_CHH_MPs	tmin_CHH_MPs	tmin_CHH_DMPs	tmax_CHH_MPs	tmin_CG_MPs	prcp_CG_MPs	tmax_CG_DMPs	tmin_CHG_DMPs	tmin_CHG_MPs	prcp_CHG_DMRs	prcp_CHG_DMRs_avg	prcp_CHG_MPs
found per input and context	5	14	0	1	2	2	0	2	3	2	0	4
terms shared by a maximum amount of inputs	2	6	0	0	2	2	0	2	1	2	0	1
found per input	**MPs**	26	**DMPs**	2	**DMRs**	2	**avg.**	0				
found per context	**CG**	4	**CHG**	11	**CHH**	20						
found per climatic data	**prcp**	8	**tmin**	21	**tmax**	1						

Figure 37: **Highlighted GO terms based on Emodel.**

Terms comprising “water”, “shoot”, “root”, and “defense” are highlighted for all inputs and contexts. Tmin_CHH_MP yields the largest number of terms, while tmin_CHH_DMPs, tmax_CG_DMPs, and prcp CHG_DMRs_averaged do not lead to any terms. MPs, CHH, and tmin yield the highest numbers of terms per input, context, and climatic data, respectively. Cells are colored from green = high to red = low.

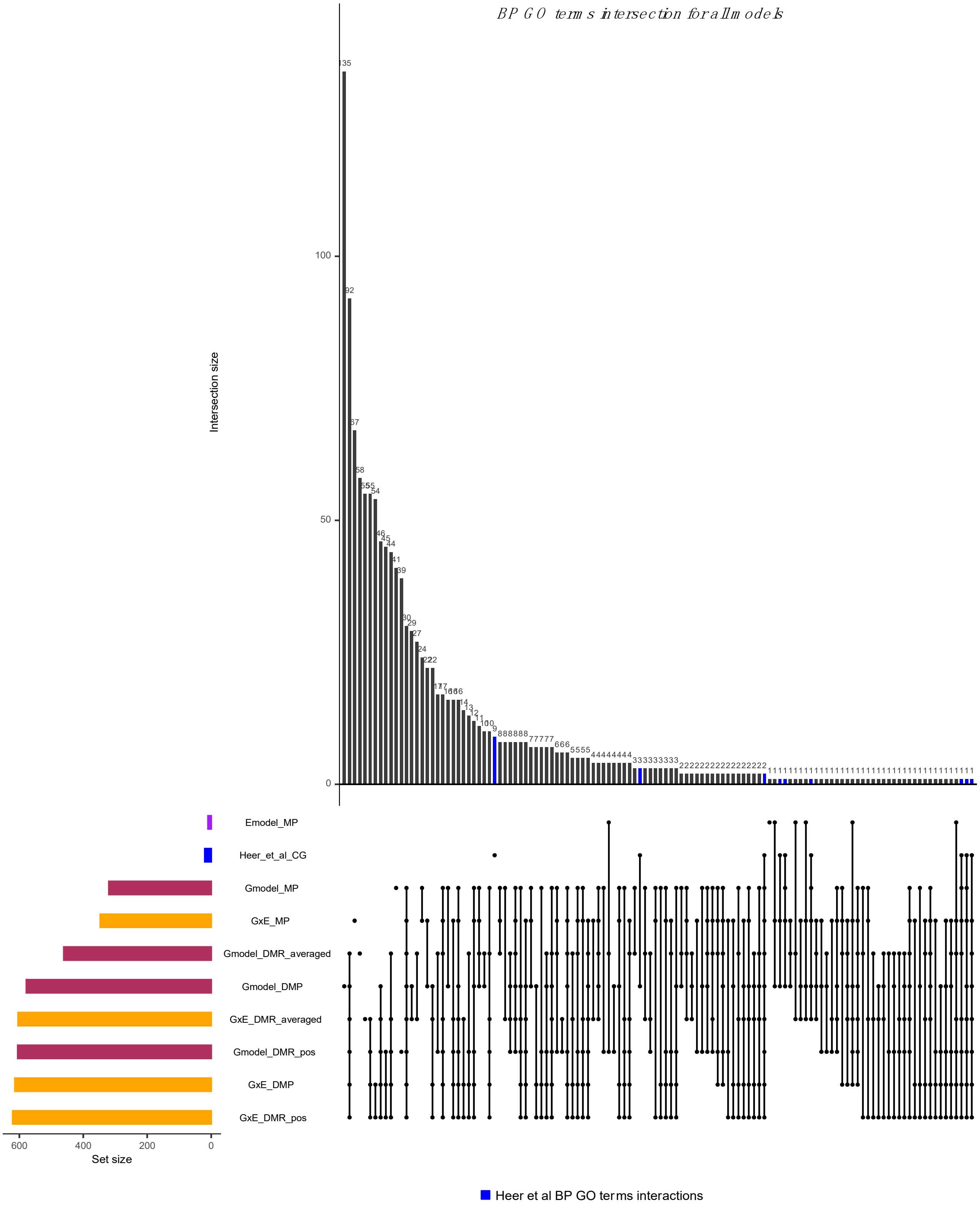

Figure S38: **Intersection of significant BP GO terms between all models, CG context, and precipitation data for location-based clustering, and a previous study [7] with the UpsetR package [8].**

The highest amount of sharing can be seen in G and GxE models DMP and DMR input types (98 terms). A term shared by seven groups as a maximum is "phenylpropanoid metabolic process". 34, 52, 160, 149, 149, 111, 225, and 397 terms are shared by eight, seven, six, five, four, three, two, and single unique inputs. In total 69% of elements are shared and only 31% are unique to single inputs. The highest number of unique elements is derived from Gmodel DMP input with 137 terms and the lowest is with one term

for the Emodel MP input. 45% of Heer et al BP terms match with the other inputs and some unmatched terms show semantic similarity.

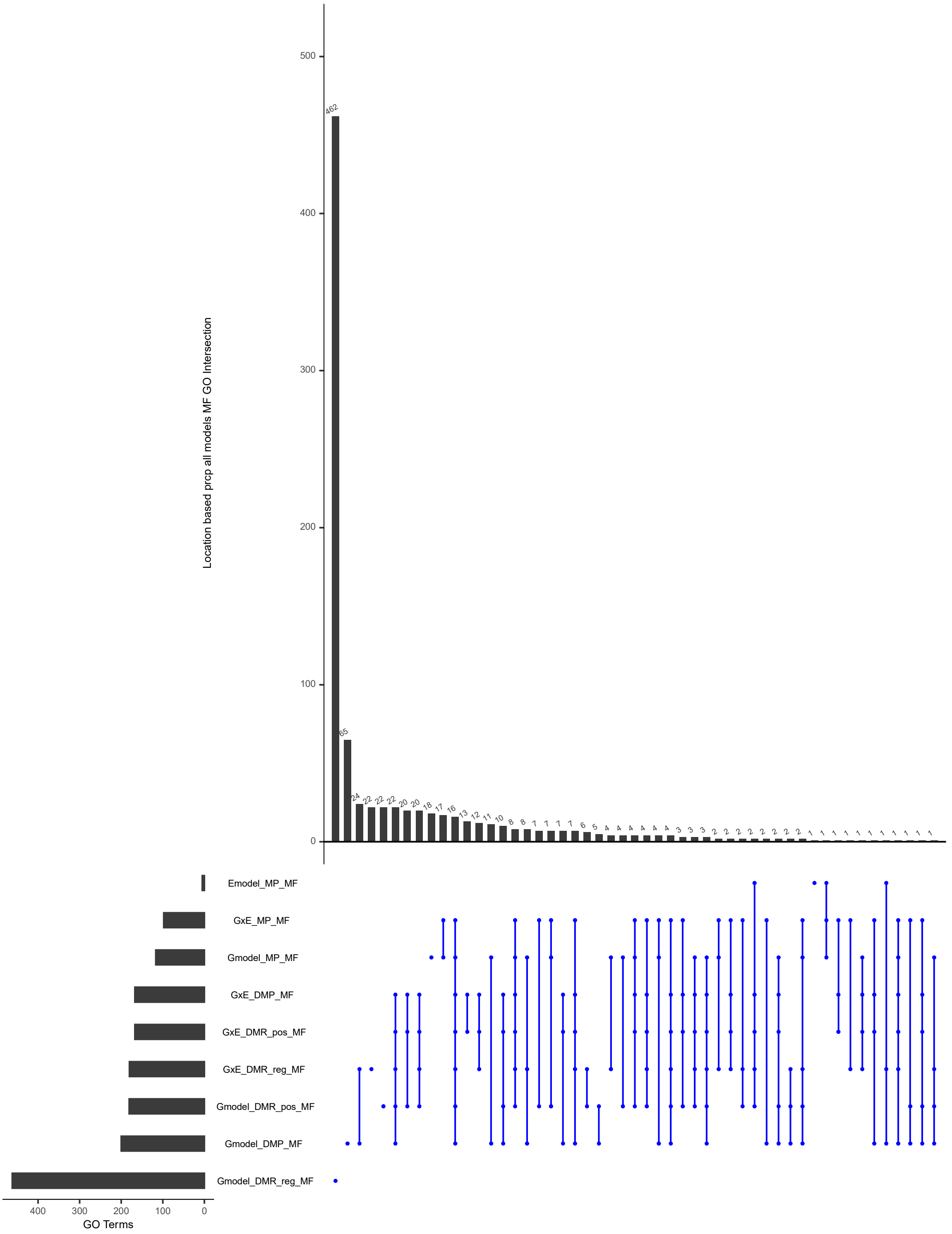

Figure S39: **Intersection of significant MF GO terms between all models, only CG context and precipitation data for location-based clustering, and a previous study [7] with UpsetR package [8].**
The highest sharing is seen in G and GxE models DMP and DMR (but reg for Gmodel) input types as 65. 13 terms are shared between seven outputs as highest intersection amount. 16, 14, 43, 44, 69, 92, and 611 terms are shared by seven, six, five, four, three, two, and single unique inputs. In total 31% of elements are shared and 69% are unique to the single inputs (reverse ratios with the BP output).

The highest number of unique elements belongs to the Gmodel DMR reg. input with 462 terms and the lowest is with one term for the Emodel MP input.

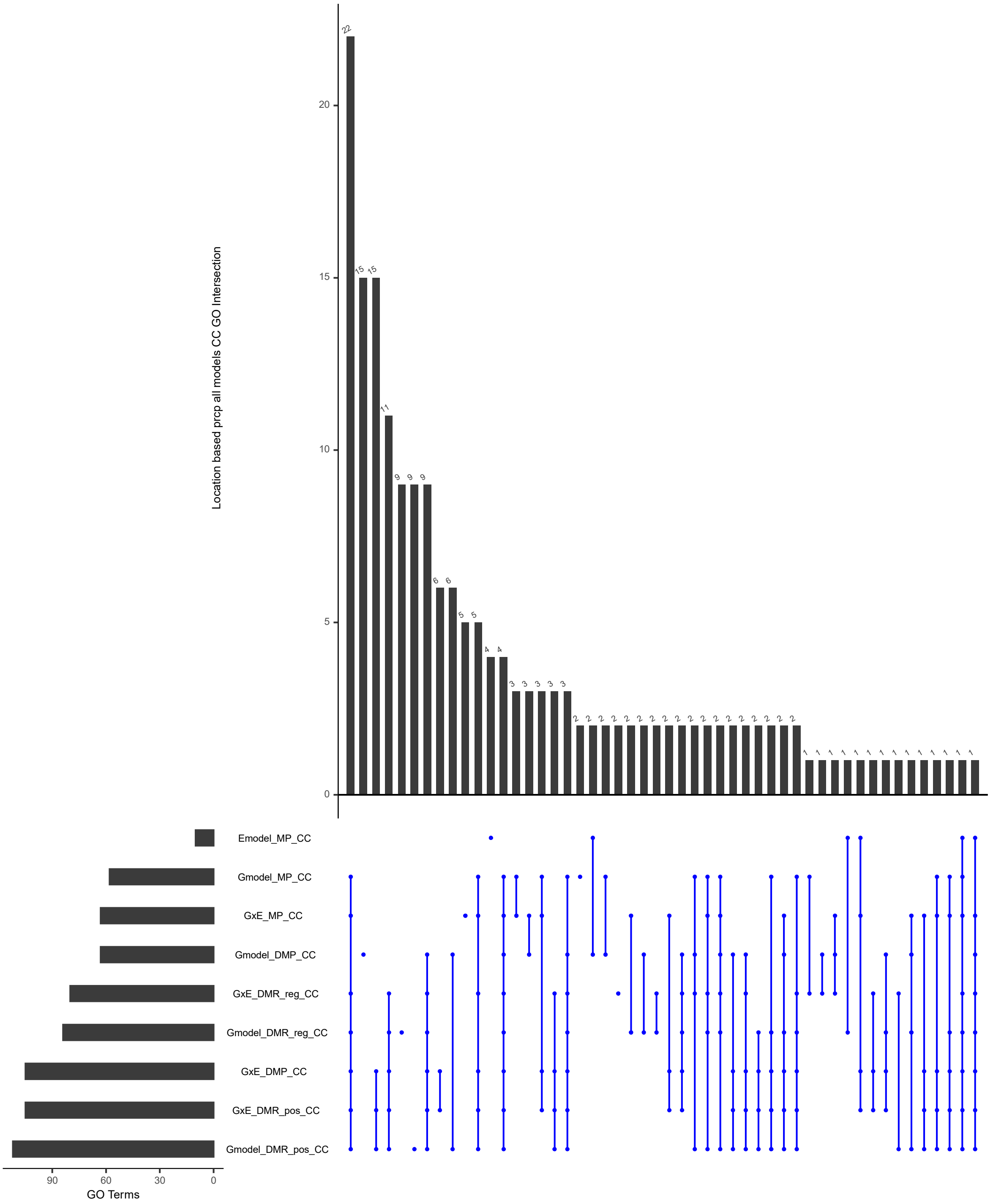

Figure S40: **Intersection of significant CC GO terms between all models, only CG context and precipitation data for location-based clustering, and a previous study [7] with UpsetR package [8].**

The highest sharing is seen between all models but the Emodel MP input type with 22 terms. The single term is shared by all outputs but Gmodel MP is “transferase complex” (on the far-right bar). 13 terms are shared between seven outputs as the highest intersection amount. 5, 26, 22, 26, 13, 22, 32, and 45 terms are shared by eight, seven, six, five, four, three, two, and single unique inputs. In total 76% of elements are shared and 23% are unique to the single inputs (reverse ratios with the BP output). The highest number of unique elements belongs to the Gmodel DMP input with 15 terms and the lowest is with two terms for the Gmodel MP and the GxE model DMR reg. input.

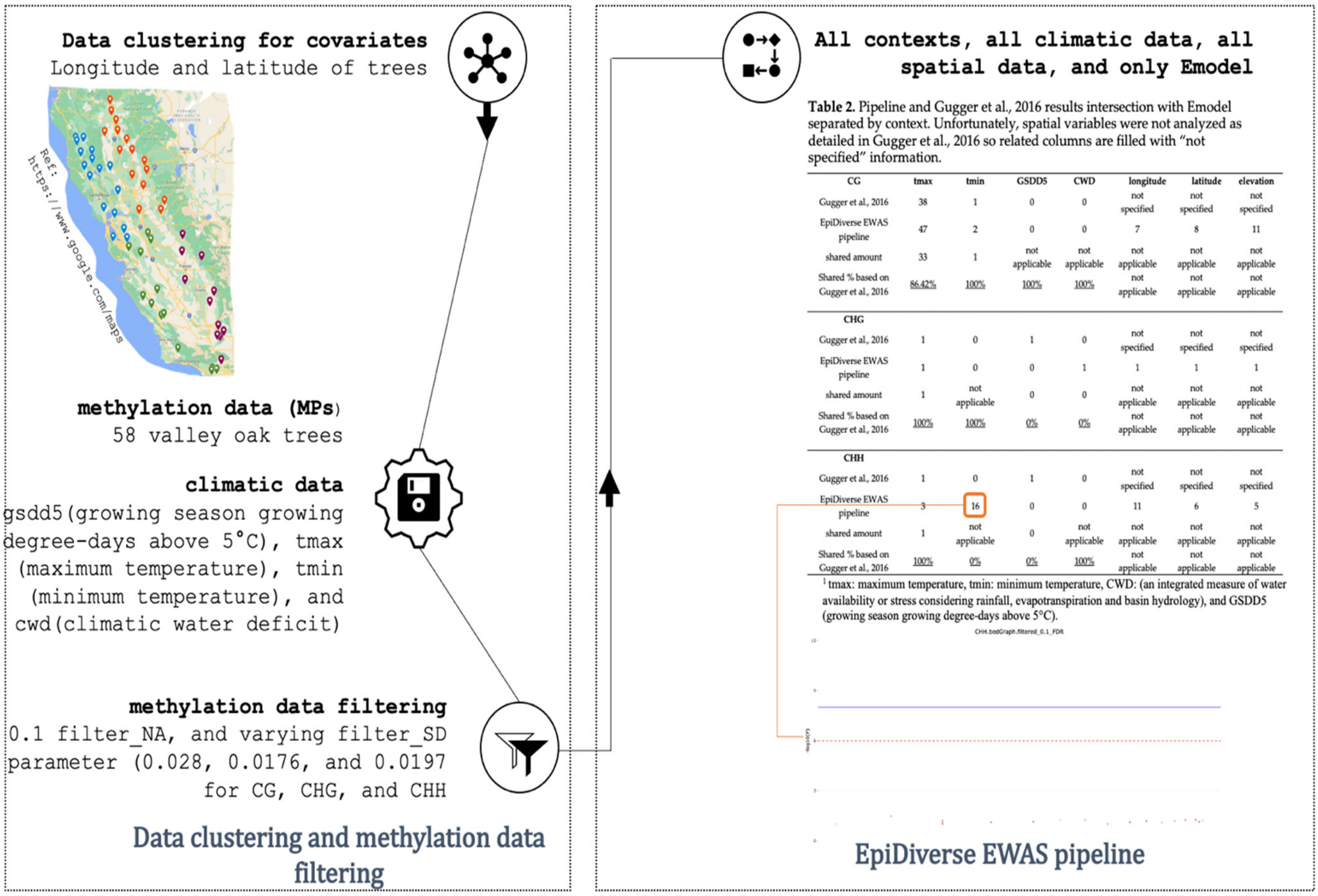

Table 2. Pipeline and Gugger et al., 2016 results intersection with Emodel separated by context. Unfortunately, spatial variables were not analyzed as detailed in Gugger et al., 2016 so related columns are filled with "not specified" information.

CG	tmax	tmin	GSDD5	CWD	longitude	latitude	elevation
Gugger et al., 2016	38	1	0	0	not specified	not specified	not specified
EpiDiverse EWAS pipeline	47	2	0	0	7	8	11
shared amount	33	1	not applicable	not applicable	not applicable	not applicable	not applicable
Shared % based on Gugger et al., 2016	86.42%	100%	100%	100%	not applicable	not applicable	not applicable
**CHG**							
Gugger et al., 2016	1	0	1	0	not specified	not specified	not specified
EpiDiverse EWAS pipeline	1	0	0	1	1	1	1
shared amount	1	not applicable	0	0	not applicable	not applicable	not applicable
Shared % based on Gugger et al., 2016	100%	100%	0%	0%	not applicable	not applicable	not applicable
**CHH**							
Gugger et al., 2016	1	0	1	0	not specified	not specified	not specified
EpiDiverse EWAS pipeline	3	16	0	0	11	6	5
shared amount	1	not applicable	0	not applicable	not applicable	not applicable	not applicable
Shared % based on Gugger et al., 2016	100%	0%	0%	100%	not applicable	not applicable	not applicable

[1] tmax: maximum temperature, tmin: minimum temperature, CWD: (an integrated measure of water availability or stress considering rainfall, evapotranspiration and basin hydrology), and GSDD5 (growing season growing degree-days above 5°C).

Figure S41: **Gugger et al., 2016 [9] methylation and climatic data processing and analysis by the EpiDiverse EWAS pipeline.** EWAS pipeline run was performed only with an Emodel and the intersection of Gugger analysis and the pipeline were outlined. An example Manhattan graph for the CHH context tmin data with the pipeline is shown below the statistic tables.

(a)

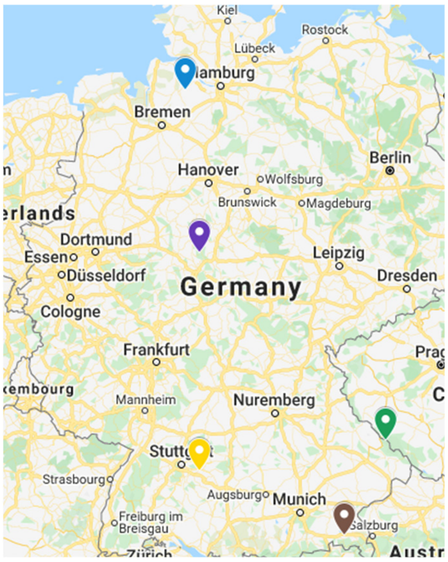

(b)

Clone ID	Location	Elevation (m)
**1836703**	West Germany Highland	300-600
**4259732**	Sweden	?
**4960703**	Other (Germany)	300-700

(c)

ramet	65	ramet2	1863703_01_G
ramet	67	ramet2	1863703_H
ramet	68	ramet2	1863703_mitte
ramet	72	ramet2	1863703_01_oben
ortet	65	ramet2	1863703_01_unten
ortet	67	ramet2	1863703_02
ortet	68	ramet2	1863703_02_H
ortet	72	ramet2	1863703_03
		ramet2	1863703_03_H
		ramet2	4259732_02_G
		ramet2	4259732_04
		ramet2	4259732_04_H
		ramet2	4960703_03_G
		ramet2	4960703_05_H
		ramet2	4960703_06_H
		ramet2	4960703_10_mitte
		ramet2	4960703_10_oben
		ramet2	4960703_10_unten
		ramet2	4960703_11
		ramet2	4960703_12

Figure S42: **Location of *P. abies* trees (a), additional clone information (b), and grouping of trees (c).**

The screenshot from Google maps with blue, purple, yellow, green, and brown colors are respectively for Harsefeld, Neuhaus, Göppingen locations with ortet and ramet growing areas (all are in Germany), ref: https://www.google.com/maps. (b) Elevation information for clone 4259732 is not known and marked as "?". Part (c) shows the grouping of trees. Ortet stands for the original tree, ramets are the clones of those ortets, and numbers (65,67, 68, and 72) are tree IDs, ramet2 trees are also clones but independent from ortets, G and H stands for Goeppingen and Harsefeld respectively. "Mitte (middle)", "oben (up)" and "unten (below)" are the trees that belong to the Neuhaus area and middle, upper, and lower parts of the trees).

The ratio of PCR Duplicates for *P. abies* dataset

The ratio of PCR duplicates by dividing them with the total number of reads were collected for the 28 *P. abies* samples (Figure S39), the average ratio was 54% and ranges between 36% to 82%. The line fit plot was showing a linear relationship with .65 R^2. Therefore, the WGBS pipeline run was conducted with –noDedup parameter, i.e., not removing PCR duplicates. Stochastically, the more fragments of a genome are analyzed, the more identical fragments are expected. That means if the genome is sequenced with high depth, more duplicates will be seen than sequencing with low depths. Hence, an approximately linear relationship between depth and duplicates is expected. During PCR, a bias might exist that leads to some of the fragments being more often amplified than others. However, such data will probably not be Poisson distributed. If such a sample is sequenced, a steeper curve than a linear relationship is expected because of the skewed distribution of the sample. In other words, it is assumed that PCR introduces a bias [10], and hence shifts the distribution of observed fragments.

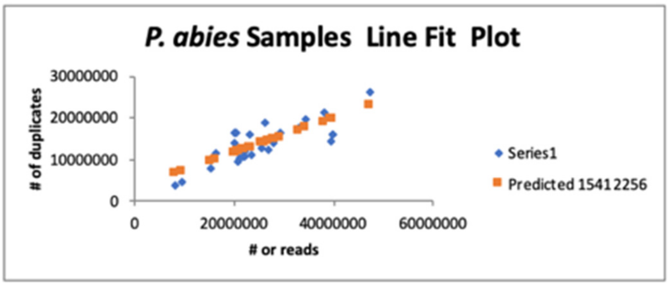

(a)

ratio [(# of duplicates) / (# of reads)]	average_ratio
0.389390719	0.54137744
0.395907094	
0.530205112	
0.462654635	
0.824350567	
0.472255475	
0.537949386	
0.562112105	
0.492288177	
0.537526155	
0.463538174	
0.493502672	
0.687897342	
0.814408895	
0.483676088	
0.721670762	
0.512367966	
0.692839721	
0.698235605	
0.506135637	
0.466641802	
0.461149251	
0.556199319	
0.55978579	
0.363372054	
0.40190483	
0.50202681	
0.568576305	

(b)

SUMMARY OUTPUT

Regression Statistics	
Multiple R	0,81056618
R Square	0,657017532
Adjusted R Square	0,643298233
Standard Error	2896720,4
Observations	27

(c)

Figure S43: **PCR duplicate analysis.**

PCR duplicate ratio (a), line fit plot with a duplicate number of duplicates vs total reads (b), and statistical summary output (c).

Blastx analysis with the *Q. lobata* dataset

13 Cs uniquely found by the pipeline with CG context and tmax data showed that three of them are genic with the closest gene hit, one shows no-hit, one was not found in a genic area with 57.0 Kb contig, and the rest found to be connected to related studies in the literature with (21-22570) bps (base pairs) distance to the closest genes. For example, receptor-like kinases show a great enlargement in the flowering plant lineage within Viridiplantae which may probably explains the gain of new roles that are essential for plants in constantly changing environment [11]. Ubiquitin carboxyl-terminal hydrolases are required for periodic maintenance of the circadian clock at high temperatures in Arabidopsis [12]. NAD biosynthesis is found to be essential in plant development and stress responses [13]. MYB58 and MYB63 are transcriptional activators of the lignin biosynthetic pathway during secondary cell wall formation in Arabidopsis [14] and Two MYB transcription factors as MYB63 and MYBR104 were stimulated in the wild type in response to heat in maize [15]. All eukaryotic circadian clocks take advantage of F-box proteins to form substrate adaptor components of a larger E3 ubiquitin ligase complex and the timing of the clock is decided by environmental factors for example light and temperature changes as well as a self-sustaining mechanism [16]. A study by Yamada et al., 2019 showed that TRANSPORT INHIBITOR RESPONSE (TIR2) is regulated by temperature and is necessary for temperature-dependent hypocotyl elongation [17]. The INO80 is the most recently well-known chromatin remodeler subfamily and it is represented by two complexes in cerevisiae INO80 and Swi2/snf2-related 1 (SWR1) [18]. H2A.Z deposition around the flowering locus (FLC) transcription start site (TSS) by SWR1 is required for FRI-mediated FLC activation and it was seen by Kumar and Wigge et al., 2010 that the temperature increases from 17 to 27 ◦C caused the eviction of the H2A.Z [19]. Ty1-copia elements are the insertion of the retrotransposon Hopscotch to the regulatory region of the gene teosinte branched1 (tb1) of the maize [20] and similar enhancing mechanisms are observed in other TE family members in maize which is for upregulating gene expression in response to abiotic stresses [21]. Wickland et al., 2015 proposed that their knowledge about FT/TFL1 gene family will let to modify florigen and anti-florigen action in the future and therefore promotes plant adaptation with climatic changes [22]. The auxin signaling pathway depends on the auxin gradient within cells and tissues, The TRYPTOPHAN AMINOTRANSFERASE OF ARABIDOPSIS (TAA)/YUCCA (YUC) pathway is the most well-known pathway to study auxin mechanisms and expression of YUC gene members are known to contribute to plant development besides environmental responses [23].

According to the pipeline results, genes found to be related to spatial variables seem found in root/shoot development systems in the literature. The closest genes with five Cs found to be related to longitude, latitude, elevation for the CG and CHG contexts showed suggestive results too none are genic but distance with [118,1357] bps to the closest gene. For example, the GDSL esterase/lipase protein (GELP) family is found to be important in plant growth and defense [24]. Naramoto et al., 2009 showed that phosphoinositide-dependent regulation of Van3 ARF-GAP localization and activity is important for vascular tissue persistence in plants [25]. The vesicle transport system appears to be crucial for plant development and environmental responses [26]. The closest gene to a single C (genic) associates with longitude for CG context encodes atypical and second-largest polymerase subunits in Arabidopsis are nuclear proteins with Pol IV and Pol V enzymes and play roles in RNA silencing and response biotic and abiotic stresses [27]. The other two closest genes to two Cs for latitude and elevation for the CG context with [2089,17001] bps distance showed that zinc finger BED domain-containing protein RICESLEEPER 3-like was found to be in stress-responsive mechanisms evolved by *S. chilense* to survive under high temperature, drought, and salt stress environment in Northern Chile of Atacama Desert [28]. Four Cs showed an association only with the elevation data for the CG context with [105,1290] bps distance to the closes gene and the literature showed that roots keep specific growth angles in many plants to the direction of gravity known as gravitropic setpoint angles (GSAs) for enough water and nutrient intake. Furutani et al., 2020 showed that some genes involved in GSA control by regulating auxin flow in Arabidopsis and RCC1- like domain (RLD) proteins are crucial regulators for polar auxin transport [29].

Tmin connected genes in the literature gave insights about environmental stress studies with various plant species. A single C found uniquely by the pipeline with CG context and tmin data was seen in a genic region and a relation to pentatricopeptide repeat (PPR) proteins. PPR proteins are one of the largest protein families in land plants and their function is found as effective on organelles biogenesis, photosynthesis, respiration, plant development, and environmental responses [30]. 16 Cs found only by the pipeline with CHH context and tmin data revealed meaningful insights too. Two of those 16 Cs are genic, one has no hits, one is not in a genic region with 17.0 Kb contig, and the rest have a distance between [116, 19,435] bps to the closest gene. The study by Baek et al., 2011 showed that overexpression of a gene encoding mitochondrial AAA protein in Arabidopsis, ATPase-in-Seed-Development (ASD), leads to morphological and anatomical seed maturation defects and it is induced by abiotic stresses like low temperature and high salinity [31]. Experiments by Kathiria et al., 2013 showed that high temperature is associated with N protein activity and therefore would result in less efficient response to tobacco mosaic virus (TMV) [32]. A KH domain-containing putative RNA-binding protein is found to be essential for heat-responsive gene regulation and thermotolerance in Arabidopsis [33]. Cysteine-rich receptor-like kinases (CRKs) were found to be related to cold, salt, drought, UV, wounding, heat, osmotic, and oxidative stress in Arabidopsis [34]. Data by Xia et al., 2017 suggested that microRNA5144 (osa-miR5144-3p) in rice responds to biotic stresses like salt, mercury, dark, and high temperature to modulate the protein disulfide isomerase (PDI) (OsPDIL1) [35]. Several studies showed that protein ubiquitination plays a very important role in plant developmental stages and abiotic stress responses such as drought and high salinity via diverse E3 ligases by mediating phytohormone and other pathways [36]. Plant peptides are key molecules of stress responses such as drought and high salinity and stress-related signaling peptides in plants found to be connected to proteolytic processing of protein precursors [37].

A single C uniquely found by the pipeline with the CG context and the CWD data seems to be connected to an uncharacterized protein for *Q.lobata* with a 1290 bps distance.

Table S1: ***Q. lobata*** **blastx analysis**

Genomic context of Cs with significant association to climate, and spatial variables including the number of Cs within the fragment, closest gene via blastx, distance to the closest gene (upstream). and putative protein product, sorted by matching status of Cs between Gugger et al., 2016 [9] study, and the EWAS pipeline. The fragment label shows the genomic contig and the position of the most significant C (contig: position) and the lowest p-values are listed for fragments with multiple significant C's. Distances are in nucleotides and measured from the closest end of the amino acid alignment.

Fragment	Climate or spatial association	Context	Climate association Q-value (min)	Closest gene via BLASTX match	Distance from BLASTX [illegible]	BLASTX Expect (E value)	Per cent of nr database
C2135867:734	tmax	CG	0.0145158	heparan-alpha-glucosaminide N-acetyltransferase [Quercus lobata]	483	3.73E-11	100
C2243561:431	tmax	CG X 2, CHH, CHG	0.00461053	none on 1.0 kb contig	NA	NA	NA
C2279863:599	tmax	CG	0.000641161	UPF0481 protein At3g47200-like [Quercus lobata]	genic	1.08E-43	61.9
C2660447:1010	tmax	CG	0.0240459	SKP1-like protein 1B-like [Tripterygium wilfordii]	-305	3.00E-22	82
C2687893:1978	tmax	CG X 6	0.0164384	mitochondrial outer membrane protein porin 4 [Quercus lobata]	394	2.00E-58	93
C2716265:837	tmax	CG	0.0274896	protein HESO1 [Quercus lobata]	9	2.00E-84	98.45
scaffold11289:53221	tmax	CG X 3	0.0145158	uncharacterized protein LOC115950208 [Quercus lobata]	11779	4.00E-73	96.55
scaffold1450:104386	tmax	CG	0.0829442	uncharacterized protein LOC115952099	-15979	0.00E+00	50.552
scaffold20751:23374	tmax	CG X 9	0.00315336	dehydration-responsive element-binding protein 1B-like [Quercus lobata]	genic	1e-107	99.17
scaffold2576:38891	tmax	CG X 2	0.0711293	short-chain dehydrogenase/reductase family 42E member 1 [Quercus lobata]	-1290	6e-79	100
scaffold27436:2761	tmax	CG	0.0264279	protein SIEVE ELEMENT OCCLUSION B-like [Quercus suber]	-5854	1.00E-04	63.16
scaffold4607:51780	tmax	CG	0.0678375	probable methyltransferase PMT5 [Quercus lobata]	-217	2.14E-14	83.6
scaffold7563:31896	tmax	CG X 2	0.0860142	exopolygalacturonase-like [Quercus lobata]	genic	5e-120	100
scaffold877:14952	tmax	CG	0.0531586	pentatricopeptide repeat-containing protein At1g20230 [Quercus lobata]	-1637	0.00E+00	99.87
scaffold9024:922	tmin, tmax	CG, CHH	0.00277591	hypothetical protein HYC85_028212 [Camellia sinensis]	8252	4.00E-04	55.32
C2163755:95	tmax	CG	0.0336844	cysteine-rich receptor-like protein kinase 19 [Quercus lobata]	-358	9.28E-55	100
C2492003:414	tmax	CG	0.0292999	ubiquitin carboxyl-terminal hydrolase MINDY-3 [Quercus lobata]	-21	0.00E+00	100

scaffold10371:36553	tmax	CG	0.0837199	uncharacterized protein At4g14450, chloroplastic-like [Quercus lobata]	11918	3.00E-66	100
scaffold11764:34472	tmax	CG	0.0902859	NAD+ synthase [Hoeflea marina]	1061	3.40E-02	78.26
scaffold12971:18452	tmax	CG	0.0902859	transcription factor MYB63-like [Quercus	-22570	0.00E+00	99.65
scaffold15605:15293	tmax	CG	0.0885572	putative F-box protein At1g47790 [Quercus lobata]	6244	0.00E+00	100
scaffold16331:7389	tmax	CG	0.0860142	RRP12-like protein [Quercus lobata]	8263	2.00E-43	98.73
scaffold18176:14869	tmax	CG	0.0711293	transport inhibitor response 1-like protein [Quercus lobata]	320	0.00E+00	100
scaffold18408:10636	tmax	CG	0.0233086	ATP-dependent helicase BRM isoform X2 [Quercus lobata]	genic	2e-105	99.4
scaffold24805:809	tmax	CG	0.0902859	no hits found	NA	NA	NA
scaffold3494:53955	tmax	CG	0.0264279	Ty1/Copia family ribonuclease HI [Bacillus coagulans]	7021	3.00E-58	50.28
scaffold41507:9942	tmax	CG	0.0860142	FT-interacting protein 3-like [Quercus lobata]	genic	0	100
scaffold6889:211950	tmax	CG	0.0829442	L-tryptophan--pyruvate aminotransferase 1 [Quercus lobata]	genic	3.46E-18	96.9
C2432489:725	tmin	CG	0.0484508	Pentatricopeptide repeat-containing protein At5g18390, mitochondrial [Quercus lobata]	genic	0	100
scaffold17237:20407	longitude, latitude, elevation	CG	0.00671081	VAN3-binding protein [Quercus lobata]	-1357	7.00E-59	80.39
C1857159:430	longitude	CG	0.0651646	DNA-directed RNA polymerases IV and V subunit 2-like [Quercus lobata]	genic	2.00E-97	100
scaffold12904:46759	longitude, latitude, and elevation	CG	0.0263294	GDSL esterase/lipase At5g45670-like [Quercus lobata]	-118	2.00E-52	95.6
scaffold24291:61775	latitude X 2 , elevation	CG	0.023653	zinc finger BED domain-containing protein RICESLEEPER 2-like [Camellia sinensis]	17001	1e-34	57.72
scaffold2525:43216	longitude, latitude, and elevation	CG X 3, CHG	1.78E-05	uncharacterized protein LOC115970248 [Quercus lobata]	1354	9e-43	75.76
scaffold9188:19867	latitude, elevation	CG	0.0186881	kinesin-like protein KIN-12C [Quercus lobata]	-2089	8e-45	97.56
scaffold34567:4783	elevation	CG	0.0933422	hypothetical protein CCACVL1_00579 [Corchorus capsularis]	-105	0.004	74.19
scaffold14291:97792	elevation	CG X 2	0.0880809	none on 40.0 kb contig	NA	NA	NA
C2692053:1750	elevation	CG	0.081777	PH, RCC1 and FYVE domains-containing protein 1-like [Quercus lobata]	1274	4e-76	100
scaffold24798:8357	CWD	CHG	0.0773968	uncharacterized protein LOC115985343 [Quercus lobata]	-1290	3.00E-59	89.8
scaffold10407:15711	tmin	CHH	0.0662687	no hits found	NA	NA	NA
scaffold10862:27958	tmin	CHH	0.098611	uncharacterized protein LOC115956251 [Quercus lobata]	genic	9e-133	83.2

scaffold14444:52956	tmin	CHH	0.0722576	uncharacterized protein LOC115959459 [Quercus lobata]	7679	0	64.99
scaffold16568:31152	tmin	CHH	0.0561036	uncharacterized protein LOC115990037 [Quercus lobata]	-116	2.00E-108	85.16
scaffold18257:4451	tmin	CHH	0.0725103	AAA-ATPase At3g50940-like [Quercus lobata]	1271	0.00E+00	100
scaffold203:20709	tmin	CHH	0.0662687	hypothetical protein CMV_020407 [Castanea mollissima]	-7650	0	100
scaffold21196:12675	tmin	CHH	0.0561036	TMV resistance protein N-like [Quercus lobata]	1993	1.00E-109	98.83
scaffold21666:33740	tmin	CHH	0.0561036	KH domain-containing protein At4g18375 [Quercus lobata]	1513	3.00E-136	98.98
scaffold2940:64285	tmin	CHH X 3	0.0602405	uncharacterized protein LOC115961955 [Quercus lobata]	genic	0.00E+00	94.32
scaffold30221:60787	tmin	CHH	0.0757422	uncharacterized protein LOC115974015 [Quercus lobata]	10842	3.00E-172	99.62
scaffold40023:13580	tmin	CHH	0.0561036	hypothetical protein [Tanacetum cinerariifolium]	-3977	1.00E-59	93.4
scaffold4751:19577	tmin	CHH	0.0787377	putative cysteine-rich receptor-like protein kinase 9 [Quercus lobata]	1342	2.00E-112	85.71
scaffold5942:282421	tmin	CHH	0.0996185	none on 17 kb contig	NA	NA	NA
scaffold7242:68670	tmin	CHH	0.0332872	uncharacterized protein LOC112019070 [Quercus suber]	19435	4e-97	82.68
scaffold8993:27871	tmin	CHH	0.0757422	protein disulfide isomerase-like 1-6 [Quercus lobata]	16819	8.00E-119	100
scaffold1177:136473	tmax	CHH	0.0790621	none on 80 kb contig	NA	NA	NA
scaffold32607:1777	longitude	CHH	0.058845	uncharacterized protein LOC115961982 [Quercus lobata]	genic	9e-161	60.62
scaffold30338:20218	longitude	CHH	0.0534505	E3 ubiquitin-protein ligase AIP2 [Quercus suber]	-211	7.00E-44	97.22
scaffold385:75210	longitude	CHH	0.0480613	none on 36 kb contig	NA	NA	NA
scaffold8012:41559	longitude	CHH	0.0436613	none on 14 kb contig	NA	NA	NA
scaffold2525:43227	longitude, latitude, and elevation, tmin	CHH X 5	0.00341586	protease Do-like 7 [Quercus suber]	7771	2.00E-57	64.89
C2705937:2352	longitude	CHH	0.0339211	hypothetical protein RHSIM_Rhsim01G0051200 [Rhododendron simsii]	733	1.00E-60	55.81
scaffold3249:231768	longitude	CHH	0.0339211	none on 18 kb contig	NA	NA	NA
scaffold5469:4524	latitude, elevation	CHH	0.0869118	uncharacterized protein LOC115963309 [Quercus lobata]	genic	0	98.17

Table S2: **Missing data estimation of EpiDiverse EWAS pipeline (a) and GEM R package (b) [38]. Missing data statistics for *P. abies* dataset with CG (c), CHG (d), and CHH (e) contexts.**

GEM's missing data estimation was misleading due to missing values, it was expected (a) that only FDR of the same position should differ colored as red and the rest should be the same as shown in green, but this was not the case (b) for GEM package. Some statistics like beta coefficient, t-statistic, and p-value were also calculated as different for the same position they only depend on the number of samples, not the total number of tests. "0" filter_NA and filter_SD parameters lead to 7%, 7% and 5% of total data for CG (c), CHG (d) and CHH (e) contexts. Different NA filtering from 0 to 0.90 intervals with 0.10 increments was used with single covariates, pairwise combinations, and all to test the effect of the filter_NA parameter. Please see the "Filtering missing data after uniting mapped reads" chapter in the main document for more details.

methylation calls output	CpG	SNP(with G and GxE models)	beta coefficient	t-statistic	p-value	FDR
	scaffold1_pos1					

DMPs output	CpG	SNP(with G and GxE models)	beta coefficient	t-statistic	p-value	FDR
	scaffold1_pos1					

DMRs output	CpG	SNP(with G and GxE models)	beta coefficient	t-statistic	p-value	FDR
	scaffold1_pos1					

(a)

methylation calls output	CpG	SNP(with G and GxE models)	beta coefficient	t-statistic	p-value	FDR
	scaffold1_pos1					

DMPs output	CpG	SNP(with G and GxE models)	beta coefficient	t-statistic	p-value	FDR
	scaffold1_pos1					

DMRs output	CpG	SNP(with G and GxE models)	beta coefficient	t-statistic	p-value	FDR
	scaffold1_pos1					

(b)

context:CG							
filter_NA	SD>=0	SD>=0.01	SD>=0.02	SD>=0.03	SD>=0.04	SD>=0.05	SD>=0.1
1 (whole data)	100	85.153	81.90743	78.61236	75.7394	73.1676	63.66231
0	7.183985	6.73756	6.009393	5.347533	4.801495	4.3389	2.539602
0.1	13.92669	12.79063	11.49225	10.32303	9.36978	8.585965	5.75164
0.2	23.37534	20.88417	18.83888	16.99949	15.5426	14.32861	10.20437
0.3	32.23377	28.14355	25.53151	23.16221	21.26519	19.6703	14.42738
0.4	40.26477	34.44791	31.49925	28.72294	26.47437	24.5761	18.41943
0.5	47.46093	39.93573	36.7995	33.75252	31.25347	29.14142	22.24315
0.6	51.94222	43.30974	40.1115	36.95909	34.3281	32.09423	24.77461
0.7	58.47812	48.22868	45.00093	41.76229	38.99429	36.60666	28.72201
0.8	65.64178	53.64643	50.40824	47.13255	44.29136	41.79891	33.33625
0.9	76.38519	61.53819	58.29262	54.99755	52.12459	49.55279	40.0475

(c)

context:CHG							
filter_NA	SD>=0	SD>=0.01	SD>=0.02	SD>=0.03	SD>=0.04	SD>=0.05	SD>=0.1
1 (whole data)	100	78.3535	73.59877	68.94692	65.12936	61.97948	52.09532
0	7.015135	6.445415	5.618783	4.924045	4.380634	3.939505	2.215696
0.1	14.25013	12.62472	10.97799	9.580412	8.499923	7.65552	4.783059
0.2	24.91511	21.01059	18.14793	15.67612	13.83572	12.38552	7.960841
0.3	34.74895	28.13361	24.33299	21.01319	18.50788	16.51788	10.78365
0.4	43.35413	33.8973	29.57225	25.62209	22.60843	20.22477	13.47159
0.5	50.86679	38.66873	34.06141	29.71799	26.35309	23.69792	16.17178
0.6	55.44294	41.51547	36.81956	32.3386	28.79566	25.99746	18.05174
0.7	61.96619	45.62302	40.88814	36.29623	32.5878	29.61434	21.14473
0.8	68.87794	50.24202	45.49515	40.86231	37.07512	34.00019	25.02301
0.9	78.90952	57.26302	52.50829	47.85644	44.03888	40.889	31.00485

(d)

(e)

context:CHH							
filter_NA	SD>=0	SD>=0.01	SD>=0.02	SD>=0.03	SD>=0.04	SD>=0.05	SD>=0.1
1 (whole data)	100	62.88156	57.26846	51.91174	47.52326	43.98433	34.77636
0	5.57226	4.386021	2.883888	1.885208	1.266226	0.880526	0.191903
0.1	10.94162	8.497514	6.029757	4.221747	3.007255	2.202042	0.581595
0.2	18.82104	13.98604	10.35435	7.507061	5.549493	4.172654	1.251026
0.3	26.56354	18.84145	14.29122	10.59255	7.980436	6.075174	1.967498
0.4	33.96631	22.8894	17.7993	13.40728	10.2282	7.873528	2.710093
0.5	41.06613	26.25673	20.8424	15.95377	12.31647	9.610056	3.525333
0.6	45.7106	28.19992	22.66701	17.58184	13.67699	10.7707	4.110746
0.7	52.78919	30.78139	25.19118	19.93012	15.74738	12.5263	5.060556
0.8	60.78352	33.18546	27.5746	22.2354	17.88863	14.46531	6.176177
0.9	72.92983	35.81139	30.19829	24.84157	20.45309	16.91416	7.706189

Table S3: **EWAS output and GO statistics.**

The uppermost table shows statistics for all models, precipitation data, CG context with location-based clustering. The following three tables are only for the Emodel, context, and separated with environment data as precipitation, tmax, and tmin respectively. Each table is also separated into contexts for CG, CHG, and CHH. No input means either no significant output is produced by the pipeline or no gene conjugate was found to perform GO analysis. No output means the GOSTAT pipeline did not yield any significant CG. Different color is for discriminating different tables, inputs, and models and have no other meaning.

Precipitation data, CG context, and location-based clustering				
model	meth_calls	DMPs	DMRs_position	DMRs_region
Gmodel	4603 (BP[320], MF [117], CC [55])	7595 (BP[320], MF [117], CC [55])	12278 (BP[607], MF [183], CC [112])	5793 (BP[462], MF [187], CC [85])
GxE	2656 (BP[358], MF [115], CC [63])	12979 (BP[623], MF [170], CC [105])	7814 (BP[544], MF [154], CC [82])	7681 (BP[594], MF [182], CC [79])
Emodel	3 (BP[10], MF [5], CC [10])	no input	no output	no input

Only Emodel and precipitation data				
CG context	meth_calls	DMPs	DMRs_position	DMRs_region
location_based_clustering	3 (BP[10], MF [5], CC [10])	no input	no output	no output
SNP_HC_based_clustering	no output	no output	no output	no output
meth_HC_based_clustering	no output	no output	no output	no output
CHG context	meth_calls	DMPs	DMRs_position	DMRs_region
location_based_clustering	21 (BP[77], MF [16], CC [8])	no input	2 (BP[24], MF [8], CC [8])	2 (BP[51], MF [10], CC [8])
SNP_HC_based_clustering	no output	no output	no output	no output
meth_HC_based_clustering	no input	no input	no input	no output
CHH context	meth_calls	DMPs	DMRs_position	DMRs_region
location_based_clustering	210 (BP[169], MF [39], CC [32])	no input	no output	no output
SNP_HC_based_clustering	no output	no output	no output	no output
meth_HC_based_clustering	no output	no output	no output	no output

Only Emodel and tmax data				
CG context	meth_calls	DMPs	DMRs_position	DMRs_region
location_based_clustering	no output	14 (BP[8], MF [8], CC [0])	no output	no output
SNP_HC_based_clustering	no output	no output	no output	no output
meth_HC_based_clustering	no output	no output	no output	no output
CHG context	meth_calls	DMPs	DMRs_position	DMRs_region
location_based_clustering	6 (BP[0], MF [8], CC [0])	no output	no output	no output
SNP_HC_based_clustering	no output	no output	no output	no output
meth_HC_based_clustering	no output	no output	no output	no output
CHH context	meth_calls	DMPs	DMRs_position	DMRs_region
location_based_clustering	56 (BP[54], MF [14], CC [8])	no output	no output	no output
SNP_HC_based_clustering	no output	no output	no output	no output
meth_HC_based_clustering	no output	no output	no output	no output

Only Emodel and tmin data				
CG context	meth_calls	DMPs	DMRs_position	DMRs_region
location_based_clustering	4 (BP[8], MF [0], CC [0])	no input	no output	no output
SNP_HC_based_clustering	no output	no output	no output	no output
meth_HC_based_clustering	no output	no output	no output	no output

CHG context	meth_calls	DMPs	DMRs_position	DMRs_region
location_based_clustering	84 (BP[92], MF [41], CC [8])	3 (BP[32], MF [0], CC [8])	no output	no output
SNP_HC_based_clustering	no output	no output	no output	no output
meth_HC_based_clustering	no output	no output	no output	no output

CHH context	meth_calls	DMPs	DMRs_position	DMRs_region
location_based_clustering	815 (BP[217], MF [95], CC [27])	14 (BP[19], MF [14], CC [8])	no output	no output
SNP_HC_based_clustering	no output	no output	no output	no output
meth_HC_based_clustering	no output	no output	no output	no output

Table S4: Statistics of additional *P. abies* samples.
Total number of reads after trimming, number of bases, and average coverage for 20 *P. abies* samples.

name	# of reads (after trimming)	# of bases	average_coverage
1863703_01_G	36,704,179	4920072696	20.4608
1863703_01mitte	39,158,493	5158271910	15.4527
1863703_01oben	52,045,993	6853371594	19.2579
1863703_01unten	36,132,791	4916389948	14.7774
1863703_02	48,798,096	6638285280	18.5874
1863703_02_H	15,180,549	2047061774	7.53737
1863703_03	37,121,421	5047374726	15.426
1863703_03_H	36,153,526	4755359364	15.7525
1863703_01_H	17,809,172	2402344197	8.32586
4259732_02_G	37,664,957	5044588256	20.0201
4259732_04	38,859,058	5267817786	15.478
4259732_04_H	47,124,752	6200261578	19.583
4960703_03_G	27,974,432	3678537518	11.8483
4960703_05_H	29,460,718	3873438994	13.8074
4960703_06_H	41,188,872	5412522166	17.5583
4960703_10mitte	47,248,888	6437003646	18.1052
4960703_10oben	39,950,533	5424276914	16.1239
4960703_10unten	36,340,407	4951833930	14.2717
4960703_11	85,163,807	11499000000	25.8284
4960703_12	70,050,616	9457219816	22.1892

Supplementary references

1. Quinlan, A.R. and I.M. Hall, *BEDTools: a flexible suite of utilities for comparing genomic features.* Bioinformatics, 2010. **26**(6): p. 841-2.

2. Jühling, F., *metilene: fast and sensitive calling of differentially methylated regions from bisulfite sequencing data*, S.H.B. Helene Kretzmer, Christian Otto , Peter F. Stadler, Steve Hoffmann, Editor. 2016.
3. Nye, T.M., P. Liò, and W.R. Gilks, *A novel algorithm and web-based tool for comparing two alternative phylogenetic trees.* Bioinformatics, 2006. **22**(1): p. 117-9.
4. Murray, K.D., et al., *kWIP: The k-mer weighted inner product, a de novo estimator of genetic similarity.* PLoS Comput Biol, 2017. **13**(9): p. e1005727.
5. Martínez-Camblor, P., S. Pérez-Fernández, and S. Díaz-Coto, *The role of the p-value in the multitesting problem, Journal of Applied Statistics.* Journal of Applied Statistics, 2020. **47:9**: p. 1529-1542.
6. Nunn, A., et al., *Manipulating base quality scores enables variant calling from bisulfite sequencing alignments using conventional Bayesian approaches.* BioRxiv, 2020. **10.1101**(2021.01.11.425926): p. 17.
7. Heer, K., et al., *Detection of somatic epigenetic variation in Norway spruce via targeted bisulfite sequencing.* Ecol Evol, 2018. **8**(19): p. 9672-9682.
8. Conway, J.R., A. Lex, and N. Gehlenborg, *UpSetR: an R package for the visualization of intersecting sets and their properties.* Bioinformatics, 2017. **33**(18): p. 2938-2940.
9. Gugger, P.F., et al., *Species-wide patterns of DNA methylation variation in Quercus lobata and their association with climate gradients.* Mol Ecol, 2016. **25**(8): p. 1665-80.
10. Parekh, S., et al., *The impact of amplification on differential expression analyses by RNA-seq.* Sci Rep, 2016. **6**: p. 25533.
11. Jose, J., S. Ghantasala, and S. Roy Choudhury, *Arabidopsis Transmembrane Receptor-Like Kinases (RLKs): A Bridge between Extracellular Signal and Intracellular Regulatory Machinery.* Int J Mol Sci, 2020. **21**(11).
12. Hayama, R., et al., *Ubiquitin carboxyl-terminal hydrolases are required for period maintenance of the circadian clock at high temperature in Arabidopsis.* Sci Rep, 2019. **9**(1): p. 17030.
13. Hashida, S.N., H. Takahashi, and H. Uchimiya, *The role of NAD biosynthesis in plant development and stress responses.* Ann Bot, 2009. **103**(6): p. 819-24.
14. Zhou, J., et al., *MYB58 and MYB63 are transcriptional activators of the lignin biosynthetic pathway during secondary cell wall formation in Arabidopsis.* Plant Cell, 2009. **21**(1): p. 248-66.
15. Casaretto, J.A., et al., *Expression of OsMYB55 in maize activates stress-responsive genes and enhances heat and drought tolerance.* BMC Genomics, 2016. **17**: p. 312.
16. Feke, A., et al., *Decoys provide a scalable platform for the identification of plant E3 ubiquitin ligases that regulate circadian function.* Elife, 2019. **8**.
17. Yamada, M., et al., *The TRANSPORT INHIBITOR RESPONSE2 gene is required for auxin synthesis and diverse aspects of plant development.* Plant Physiol, 2009. **151**(1): p. 168-79.
18. Ojolo, S.P., et al., *Regulation of Plant Growth and Development: A Review From a Chromatin Remodeling Perspective.* Front Plant Sci, 2018. **9**: p. 1232.
19. Kumar, S.V. and P.A. Wigge, *H2A.Z-containing nucleosomes mediate the thermosensory response in Arabidopsis.* Cell, 2010. **140**(1): p. 136-47.
20. Galindo-González, L., et al., *LTR-retrotransposons in plants: Engines of evolution.* Gene, 2017. **626**: p. 14-25.
21. Makarevitch, I., et al., *Correction: Transposable Elements Contribute to Activation of Maize Genes in Response to Abiotic Stress.* PLoS Genet, 2015. **11**(10): p. e1005566.
22. Wickland, D.P. and Y. Hanzawa, *The FLOWERING LOCUS T/TERMINAL FLOWER 1 Gene Family: Functional Evolution and Molecular Mechanisms.* Mol Plant, 2015. **8**(7): p. 983-97.
23. Cao, X., et al., *The Roles of Auxin Biosynthesis YUCCA Gene Family in Plants.* Int J Mol Sci, 2019. **20**(24).
24. Li, H., et al., *Identification and expression analysis of the GDSL esterase/lipase family genes, and the characterization of.* PeerJ, 2019. **7**: p. e6741.
25. Naramoto, S., et al., *Phosphoinositide-dependent regulation of VAN3 ARF-GAP localization and activity essential for vascular tissue continuity in plants.* Development, 2009. **136**(9): p. 1529-38.
26. Carland, F. and T. Nelson, *CVP2- and CVL1-mediated phosphoinositide signaling as a regulator of the ARF GAP SFC/VAN3 in establishment of foliar vein patterns.* Plant J, 2009. **59**(6): p. 895-907.
27. Ream, T.S., et al., *Subunit compositions of the RNA-silencing enzymes Pol IV and Pol V reveal their origins as specialized forms of RNA polymerase II.* Mol Cell, 2009. **33**(2): p. 192-203.
28. Kashyap, S.P., et al., *Understanding salt tolerance mechanism using transcriptome profiling and de novo assembly of wild tomato Solanum chilense.* Sci Rep, 2020. **10**(1): p. 15835.
29. Furutani, M., et al., *Polar recruitment of RLD by LAZY1-like protein during gravity signaling in root branch angle control.* Nat Commun, 2020. **11**(1): p. 76.
30. Barkan, A. and I. Small, *Pentatricopeptide repeat proteins in plants.* Annu Rev Plant Biol, 2014. **65**: p. 415-42.
31. Baek, K., P.J. Seo, and C.M. Park, *Activation of a mitochondrial ATPase gene induces abnormal seed development in Arabidopsis.* Mol Cells, 2011. **31**(4): p. 361-9.
32. Kathiria, P., et al., *Effect of external and internal factors on the expression of reporter genes driven by the N resistance gene promoter.* Plant Signal Behav, 2013. **8**(7): p. e24760.
33. Guan, Q., et al., *A KH domain-containing putative RNA-binding protein is critical for heat stress-responsive gene regulation and thermotolerance in Arabidopsis.* Mol Plant, 2013. **6**(2): p. 386-95.

34. Burdiak, P., et al., *Cysteine-rich receptor-like kinase CRK5 as a regulator of growth, development, and ultraviolet radiation responses in Arabidopsis thaliana.* J Exp Bot, 2015. **66**(11): p. 3325-37.
35. Xia, K., et al., *Formation of Protein Disulfide Bonds Catalyzed by OsPDIL1;1 is Mediated by MicroRNA5144-3p in Rice.* Plant Cell Physiol, 2018. **59**(2): p. 331-342.
36. Shu, K. and W. Yang, *E3 Ubiquitin Ligases: Ubiquitous Actors in Plant Development and Abiotic Stress Responses.* Plant Cell Physiol, 2017. **58**(9): p. 1461-1476.
37. Chen, Y.L., et al., *The role of peptides cleaved from protein precursors in eliciting plant stress reactions.* New Phytol, 2020. **225**(6): p. 2267-2282.
38. Pan, H., et al., *Gene, Environment and Methylation (GEM): a tool suite to efficiently navigate large scale epigenome wide association studies and integrate genotype and interaction between genotype and environment.* BMC Bioinformatics, 2016. **17**: p. 299.
